# A Novel Method to Verify Multilevel Computational Models of Biological Systems Using Multiscale Spatio-Temporal Meta Model Checking

**DOI:** 10.1371/journal.pone.0154847

**Published:** 2016-05-17

**Authors:** Ovidiu Pârvu, David Gilbert

**Affiliations:** Department of Computer Science, College of Engineering, Design and Physical Sciences, Brunel University London, London, United Kingdom; King’s College London, UNITED KINGDOM

## Abstract

Insights gained from multilevel computational models of biological systems can be translated into real-life applications only if the model correctness has been verified first. One of the most frequently employed *in silico* techniques for computational model verification is model checking. Traditional model checking approaches only consider the evolution of numeric values, such as concentrations, over time and are appropriate for computational models of small scale systems (e.g. intracellular networks). However for gaining a systems level understanding of how biological organisms function it is essential to consider more complex large scale biological systems (e.g. organs). Verifying computational models of such systems requires capturing both how numeric values and properties of (emergent) spatial structures (e.g. area of multicellular population) change over time and across multiple levels of organization, which are not considered by existing model checking approaches. To address this limitation we have developed a novel approximate probabilistic multiscale spatio-temporal meta model checking methodology for verifying multilevel computational models relative to specifications describing the desired/expected system behaviour. The methodology is generic and supports computational models encoded using various high-level modelling formalisms because it is defined relative to time series data and not the models used to generate it. In addition, the methodology can be automatically adapted to case study specific types of spatial structures and properties using the spatio-temporal meta model checking concept. To automate the computational model verification process we have implemented the model checking approach in the software tool Mule (http://mule.modelchecking.org). Its applicability is illustrated against four systems biology computational models previously published in the literature encoding the rat cardiovascular system dynamics, the uterine contractions of labour, the *Xenopus laevis* cell cycle and the acute inflammation of the gut and lung. Our methodology and software will enable computational biologists to efficiently develop reliable multilevel computational models of biological systems.

## Introduction

Multilevel computational models of complex biological systems are abstract representations of living systems that span multiple levels of organization. They encode the hierarchical organization of biological systems explicitly, and therefore enable reasoning about how events initiated at one level of organization reflect across multiple levels of organization. In systems biology [1, 2] multilevel, also commonly referred to as multiscale [3] computational models can be employed for gaining a better understanding of the underlying mechanisms of living systems, and to generate new hypotheses for driving experimental studies. Conversely in systems medicine it is argued [4] that multilevel computational models could potentially facilitate delivering personalized treatments by providing a patient specific understanding of how diseases and their treatment reflect across multiple levels of organization [5].

However any insights gained from model simulation results can be successfully translated into real-life applications only if the correctness of the models has been verified first. Computational models of biological systems can be validated either in the *in vitro* environment by checking if the model simulation results can be reproduced experimentally, or in the *in silico* environment by verifying if the model simulation results conform to a formal specification describing the desired/expected system behaviour. An *in silico* approach that automates the process of verifying models relative to formal specifications is called model checking [6, 7]; see S1 Text for a brief description of model checking. Due to the complex, stochastic nature of biological systems only approximate probabilistic model checking approaches are considered throughout this paper.

Validating multilevel computational models in the *in vitro* environment is challenging because there is a need for experimental data from all levels of organization and the interactions between different levels, which is often not available. Moreover *in vitro* validation procedures need to account for the variability inherent in biological systems [8, 9] which can be of different orders of magnitude at different levels. Conversely, verifying multilevel computational models in the *in silico* environment is challenging because there is a lack of model checking approaches that can explicitly distinguish between different levels of organization. Existing model checking approaches can be employed to verify submodels corresponding to each level of organization individually without the possibility of referring to interactions between different levels.

In this paper we address this issue by developing a novel multiscale model checking methodology for automatically verifying multilevel computational models relative to given specifications. Our approach is generic and supports computational models encoded using various high-level modelling formalisms because it is defined relative to time series data representing the model simulation results and not the models themselves. Moreover our methodology could be potentially employed for analysing time series data recorded in the wet-lab as well. This could enable checking if a computational model correctly describes a physical system, or that a physical system correctly implements an *in silico* design, but this is beyond the scope of this paper.

Both spatial and non-spatial computational models can be verified using our approach. The specifications against which the computational models are verified can describe both how numeric values (e.g. concentration of protein X) and properties of (emergent) spatial structures, called spatial entities, (e.g. area of multicellular population) are expected to change over time and across multiple levels of organization. For instance, assuming we would like to verify a computational model describing tumour growth, the specification could state that if the concentration of protein X in a cancerous cell rises above a certain threshold level (e.g. 0.8 M), then the cell will divide and the cellular density or area of the tumour (structure) will increase.

Assuming that the computational model considered is spatial, the type of spatial entities and their properties, called spatial measures, can differ between case studies. For instance given a tumour growth computational model one could be potentially interested in how the area of the tumour structure changes over time, whereas in case of a migrating multicellular population tracking the position of the population over time could be of interest.

We defined an abstraction of our approach, called multiscale spatio-temporal *meta* model checking that enables the automatic reconfiguration of the model checking methodology according to case study specific spatial entity types and measures. The spatio-temporal *meta* model checking approach resembles the meta-programming [10] concept from computer science where an *abstract* type is defined that acts as a template for creating *specific* type instances tailored to particular applications. Our spatio-temporal meta model checking approach is not restricted to biologically relevant spatial entity types and properties, and therefore could be employed to adapt the methodology to case studies from other fields of science. However we do not illustrate this in this paper. Due to the intended general applicability of the approach, and the fact that hierarchical systems in multiple domains of science (e.g. astrophysics, energy, engineering, environmental science and materials science [11]) are commonly referred to as multiscale, our approach is called multiscale rather than multilevel spatio-temporal meta model checking.

To enable the automatic verification of multilevel computational models of biological systems relative to formal specifications we have implemented the model checking method in the software tool Mule which is made freely available online (http://mule.modelchecking.org) in binary and source code format. Moreover a Docker [12] image has been created that provides a self-contained environment for running Mule without additional setup on all major operating systems.

We illustrate the applicability of Mule by verifying the correctness of four multilevel computational models previously published in the literature. The models considered are of different complexity, have been encoded using different modelling formalisms and software, are deterministic, stochastic or hybrid, and encode space explicitly or not. The case studies corresponding to the four multilevel computational models are the rat cardiovascular system dynamics [13], the uterine contractions of labour [14], the *Xenopus laevis* cell cycle [15], and the acute inflammation of the gut and lung [16]. The formal specifications against which the models are verified were derived from the original papers introducing the models. The main reason for this is that in the following we focus on describing the model verification methodology and not on presenting novel biologically relevant results.

In brief, the main contributions of our paper are:

1. Definition of a multiscale spatio-temporal model checking methodology for verifying multilevel computational models of biological systems relative to formal specifications describing the desired/expected system behaviour.
2. Definition of the spatio-temporal meta model checking concept which enables automatically reconfiguring the methodology according to case study specific spatial entity types and measures.
3. Implementation of the multiscale spatio-temporal meta model checking approach in the freely available software Mule. Both Bayesian and frequentist model checking algorithms can be employed to verify multilevel computational models (considering user-defined error bounds).
4. Illustrative examples of how to verify multilevel computational models of biological systems using multiscale spatio-temporal meta model checking.

### Related work

In computational (systems) biology, model checking approaches have been employed for model verification [17–32], parameter estimation/synthesis [33–42], model construction (i.e. both model parameters and structure/topology) [43, 44], and robustness computation (considering various perturbations) [39, 44–47]; see recent review papers [48–50] for a more detailed description.

One common characteristic of these model checking approaches is that they only consider how numeric values (e.g. concentrations) change over time. They are appropriate for small scale systems where the spatial domain is usually not represented explicitly (e.g. cell cycle [23, 27, 32, 36, 44, 46, 51], gene expression/regulatory networks [20, 35, 39, 52, 53], signalling pathways [17, 22, 25, 28–30, 38, 46, 54–56]). These model checking approaches cannot be directly employed to verify either spatial computational models because they do not consider how spatial properties change over time, or multilevel computational models because they do not distinguish between different levels of organization.

In previous work [57] we have defined a model checking methodology which enables verifying computational models of biological systems with respect to both how numeric values and spatial properties change over time. However the main limitation of this approach is that it cannot explicitly distinguish between different levels of organization and therefore cannot be employed to verify multilevel computational models of biological systems. Moreover the types of spatial entities and measures are hardcoded in the methodology and cannot be reconfigured according to the model verification requirements of different case studies.

## Methods

**Fig 1:**
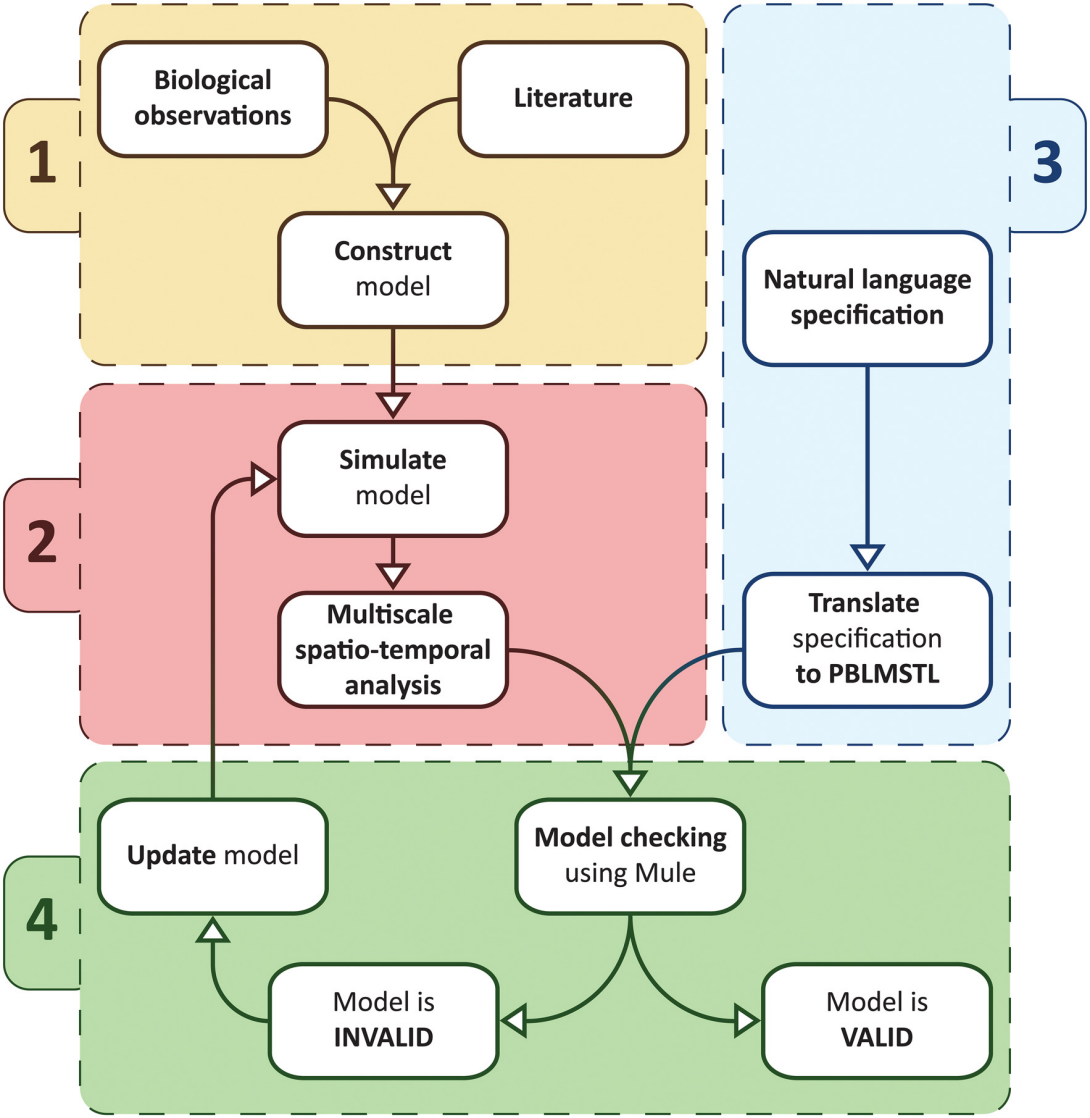
Multiscale spatio-temporal model checking workflow. The first step (1) in the workflow is using biological observations and/or information from the literature to construct the multilevel computational model of the biological system considered. Next (2) the model is simulated to produce time series data in which spatial entities from multiple scales are automatically detected and analysed using a multiscale spatio-temporal analysis module. Then (3) the specification against which the model is verified is translated from natural language to a formal multiscale spatio-temporal language called PBLMSTL. Finally (4) using the model checker Mule the model is automatically verified relative to the given PBLMSTL specification considering the processed time series data representing the modelled system behaviour. If the model is declared incorrect relative to the given specification then it is updated and the steps (2) and (4) are repeated.

Using the novel model checking approach introduced in this paper multilevel computational models of biological systems can be verified relative to formal specifications as described by the workflow depicted in Fig 1, which comprises four steps:

1. **Model construction:** Using biological observations and/or relevant references from the literature to construct the computational model.
2. **Multiscale spatio-temporal analysis:** Each time the model is simulated time series data are generated in which spatial entities from multiple scales are automatically detected and analysed.
3. **Formal specification:** The specification of the system is mapped from natural language into formal logic.
4. **Model checking:** The model checker takes as input the processed time series data (representing the behaviour of the modelled system) and the formal specification, and verifies if the model is correct relative to the specification using the model checking algorithm chosen by the user (e.g. frequentist statistical model checking). In the case that the model is incorrect it is updated and verified again.

### Model construction

The biological systems considered here are assumed to be inherently complex, stochastic, and to span multiple levels of organization [58], where different levels of organization correspond to different spatio-temporal scales. Moreover we assume in the following that biological systems which are multilevel (i.e. span multiple levels of biological organization) are inherently multiscale (i.e. span multiple spatio-temporal scales). Therefore the terms multiscale and multilevel are used interchangeably in this paper. However, since our methodology is “multiscale” instead of “multilevel” we will refer to “scales” rather than “levels” when describing it. The multiscale system representation is assumed to be hierarchical, with the most coarse-grained scales represented at the top of the hierarchy and the most fine-grained scales at the bottom. Time can be represented either in a discrete (using non-negative integer values) or continuous (using non-negative real values) manner. Whenever space is represented explicitly, we assume throughout, similarly to our previous work [57], that it is discretised and represented in pseudo-3D i.e. 2D space in which pile up is allowed, where the degree of pile up for each spatial position is computed using a density measure (e.g. representing cellular density). However adapting the methodology to other numbers of spatial dimensions requires minor changes which are described later. Furthermore we consider that the behaviour of such systems can be represented as sequences of discrete states where the system probabilistically transitions between states only when an event (e.g. a biochemical reaction) occurs.

Such systems are usually represented using high-level modelling languages (e.g. agent based models, cellular automata etc.), examples of which are given in the Results section. However, for model checking purposes, the behaviour of the computational models is usually described using an equivalent low level representation (e.g. a state transition system). The main reason for this is to enable defining the model checking algorithms relative to a single common rather than multiple different model representations.

Low level modelling formalisms often employed to encode systems that have the above mentioned properties are stochastic discrete-event systems (SDES) [59] when no constraint is imposed on the representation of time, respectively discrete-time/continuous-time Markov chains (DTMC/CTMC) when time is assumed to be discrete/continuous. One limitation of SDESs (and DTMCs/CTMCs) is that they do not explicitly distinguish between how numeric and spatial properties of the system change over time and across multiple scales. An extension of SDESs called stochastic spatial discrete-event systems (SSpDES) was defined in [57] to enable explicitly differentiating between numeric and spatial properties. However, similarly to SDESs, SSpDESs do not enable distinguishing between different scales.

In order to address this issue a multiscale extension of SSpDESs called *Multiscale Stochastic Spatial Discrete Event Systems*, or MSSpDES for short, is defined next. Formally an MSSpDES M is a 9-tuple 〈*S*, *T*, *μ*, *NSV*, *SpSV*, *NV*, *CSpV*, *MA*, *SVSS*〉 where:

- *S* = {*s*_0_, *s*_1_, …, *s*_*k*_} is the set containing all possible *states* of the system.
- *T* is the set representing *time* and it is typically equal to the set of non-negative integer numbers in case of a discrete-time representation (i.e. *T* = ℤ_+_), respectively the set of non-negative real numbers in case of a continuous-time representation (i.e. *T* = ℝ_+_).
- *μ* is a *probability measure* employed to compute the probability of the system to transition along the sequences of states described by a collection of model simulation traces. In case of biological systems it is often assumed that the Markov (memoryless) property holds i.e. the probability of the systems to transition between states depends only on the current and not on previous states. Considering this assumption, if a discrete-time representation is employed then *μ* is defined similarly as for DTMCs [60] relative to a transition probability function **P**: *S* × *S* → [0, 1] which records the probability of transitioning between any two states *s*_*i*_, *s*_*j*_ ∈ *S*. Conversely, if a continuous-time representation is employed then *μ* is defined similarly as for CTMCs [61] considering a transition rate matrix **Q**: *S* × *S* → ℝ which records the rate at which a system transitions between any two states *s*_*i*_, *s*_*j*_ ∈ *S* and from which the corresponding state transition probabilities can be derived.
- *NSV* = {*nsv*_1_, *nsv*_2_, …, *nsv*_*l*_} is the set of *numeric state variables* describing the state of the system.
- *SpSV* = {*spsv*_1_, *spsv*_2_, …, *spsv*_*m*_} is the set of *spatial state variables* describing the state of the system.
- *NV*: *S* × *NSV* → ℝ is the *numeric value assignment function* employed to compute for a given state of the system *s* ∈ *S* the value *val*_*NSV*_ ∈ ℝ of the numeric state variable *nsv* ∈ *NSV*, where *val*_*NSV*_ = *NV*(*s*, *nsv*).
- *CSpV* = {*SpV*_1_, *SpV*_2_, …, *SpV*_*n*_} is the *collection of spatial value assignment functions*, where each *spatial value assignment function* *SpV*_*i*_ ∈ *CSpV*, $Sp\;V{\;}_{i}:S\times SpSV\to \mathbb{R}{}^{m_{i}\times n_{i}}$, is employed to compute for a given state of the system *s* ∈ *S* the value $val_{SpSV}\in \mathbb{R}{}^{m_{i}\times n_{i}}$ of spatial state variable *spsv* ∈ *SpSV* that corresponds to a discretised spatial domain of size *m*_*i*_ × *n*_*i*_, where *val*_*SpSV*_ = *SpV*_*i*_(*s*, *spsv*).
- *MA* = (*V*_*MA*_, *E*_*MA*_) is the multiscale architecture graph encoding the hierarchical multiscale structure of the system under consideration.
- *SVSS*: *NSV* ∪ *SpSV* → *V*_*MA*_ is the *state variable scale and subsystem assignment function* which associates each state variable *sv* ∈ *NSV* ∪ *SpSV* with a vertex *v*_*scsubsys*_ ∈ *V*_*MA*_ encoding a particular scale and subsystem, where *v*_*scsubsys*_ = *SVSS*(*sv*).

The *multiscale architecture* graph *MA* = (*V*_*MA*_, *E*_*MA*_) is employed to formally encode the hierarchical top-down structure of multiscale systems and is represented as a rooted (directed) tree, where *V*_*MA*_ represents the set of vertices and *E*_*MA*_ the set of directed edges. The main reason for choosing the rooted directed tree representation is that its structure is inherently hierarchical and therefore similar to the organization of biological organisms. We assume throughout that vertices higher in the tree correspond to coarse-grained scales, and vertices lower in the tree correspond to fine-grained scales. Each vertex *v* ∈ *V*_*MA*_ is encoded as a tuple (*sc*, *subsys*) where *subsys* represents a particular biological subsystem (e.g. heart) and *sc* its corresponding scale (e.g. organ). Both scales and subsystems are recorded by the *MA* graph to enable distinguishing between different scales (e.g. organ and cellular), and/or different subsystems (e.g. heart and liver) corresponding to the same scale (e.g. organ). Directed edges (*v*, *v*_*i*_) ∈ *E*_*MA*_, $i=\bar{1,m}$, link the biological subsystem represented by vertex *v* to all its *m* constituent subsystems from finer-grained scales represented by vertices *v*_*i*_.

The assumption made here is that biological systems can be decomposed in a top-down manner from coarse-grained (e.g. population/organism) to fine-grained (e.g. intracellular/molecular) scales. Moreover at each scale (e.g. organ) one or multiple biological subsystems (e.g. heart and kidney) could be explicitly considered. The number and type of biological subsystems and/or scales considered differs depending on the biological question addressed. A description of how to construct the *MA* graph corresponding to a given biological system is given in S2 Text.

Considering that the *MA* graph is represented as a rooted directed tree, a strict partial order < can be defined over the set of vertices *V*_*MA*_, where *v*_1_ < *v*_2_, for all *v*_1_, *v*_2_ ∈ *V*_*MA*_, if the unique path from the root to *v*_1_ passes through *v*_2_. Similarly a non-strict partial order ≤ can be defined over *V*_*MA*_, where *v*_1_ ≤ *v*_2_ if the unique path from the root to *v*_1_ passes through *v*_2_, or *v*_1_ = *v*_2_. One of the main practical benefits of defining these partial orders is that they enable writing expressions for referring to all subsystems *v*_*i*_ of a system *v*_*j*_ (*v*_*i*_ ≤ *v*_*j*_), and all ancestor/parent systems *v*_*k*_ of a subsystem *v*_*l*_ (*v*_*l*_ < *v*_*k*_) in a concise manner. Therefore such expressions could be employed to write shorter formal specifications against which the computational models are verified.

A simple illustrative example of how to construct a (discrete-time) MSSpDES model for a biological system spanning multiple levels of organization is given below.

#### Example 1 Simple illustrative example of how to construct an MSSpDES model

Let us assume that we would like to model the movement (considering the von Neumann neighbourhood relation) of a unicellular microorganism in a fixed size environment (here a discretised rectangular grid of size 2 × 2). In order to move, the cell requires energy which it can chemically convert from an abstractly denoted nutrient *A*; the chemical reaction for converting *A* to energy is *A* → *Energy*. If nutrient *A* is available intracellularly then it can be converted directly to energy. Otherwise it has to be assimilated from the environment first; the cell can only assimilate nutrients from the position of the discretised space which it currently occupies. The probability of the cell to move is 20%, respectively 30% to convert *A* to energy and 50% to assimilate *A* from the environment.

Although the system considered in this example is much simpler than a real-life one, it suffices to illustrate the principles of abstractly representing a multiscale stochastic spatial discrete-event system. Throughout this example a discrete time representation is employed.

**Fig 2:**
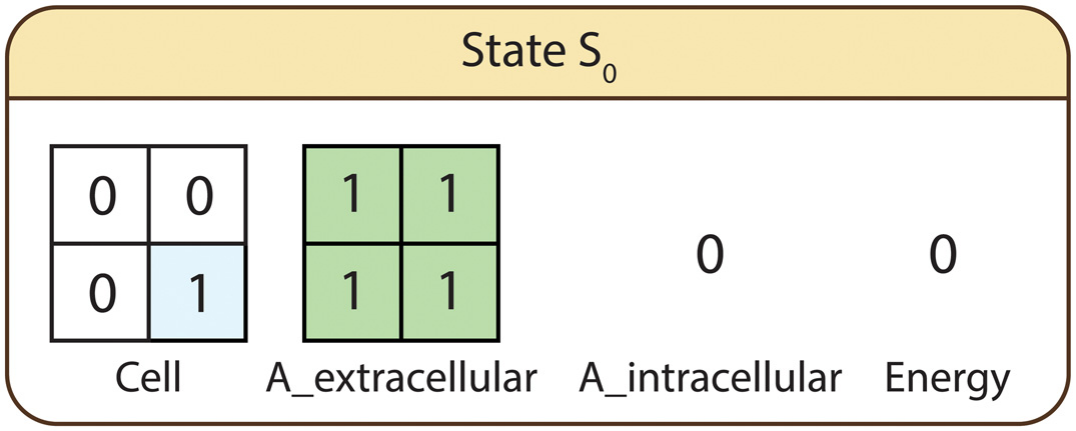
Initial state of the system. *Cell* and *A*_*extracellular* are the spatial state variables representing the position of the cell, respectively distribution of nutrient *A* in the environment. *A*_*intracellular* and *Energy* represent the intracellular availability of nutrient *A*, respectively energy.

The spatial state variables employed to describe the behaviour of the system are *Cell*—encoding the position of the cell in the discretised space, and *A*_*extracellular*—representing the distribution of nutrient *A* in the environment. Conversely the employed numeric state variables are *A*_*intracellular*—encoding the intracellular availability of nutrient *A*, and *Energy*—representing the cell’s energy supply. The considered subsystems and corresponding scales are energy production reaction network at the intracellular scale, microorganism at the cellular scale, and growth media at the environment scale. State variables associated with the energy production reaction network (intracellular scale) are *A*_*intracellular* and *Energy*, respectively *Cell* with the microorganism (cellular scale), and *A*_*extracellular* with the growth media (environment scale). In the initial state (*S*_0_) of the system, depicted in Fig 2, the cell is positioned in the lower right part of the environment, *A*_*extracellular* is uniformly distributed across the entire environment (*A*_*extracellular*[*i*, *j*] = 1, for all $i,j=\bar{1,2}$), and the initial levels of *A*_*intracellular* and *Energy* are zero.

**Fig 3:**
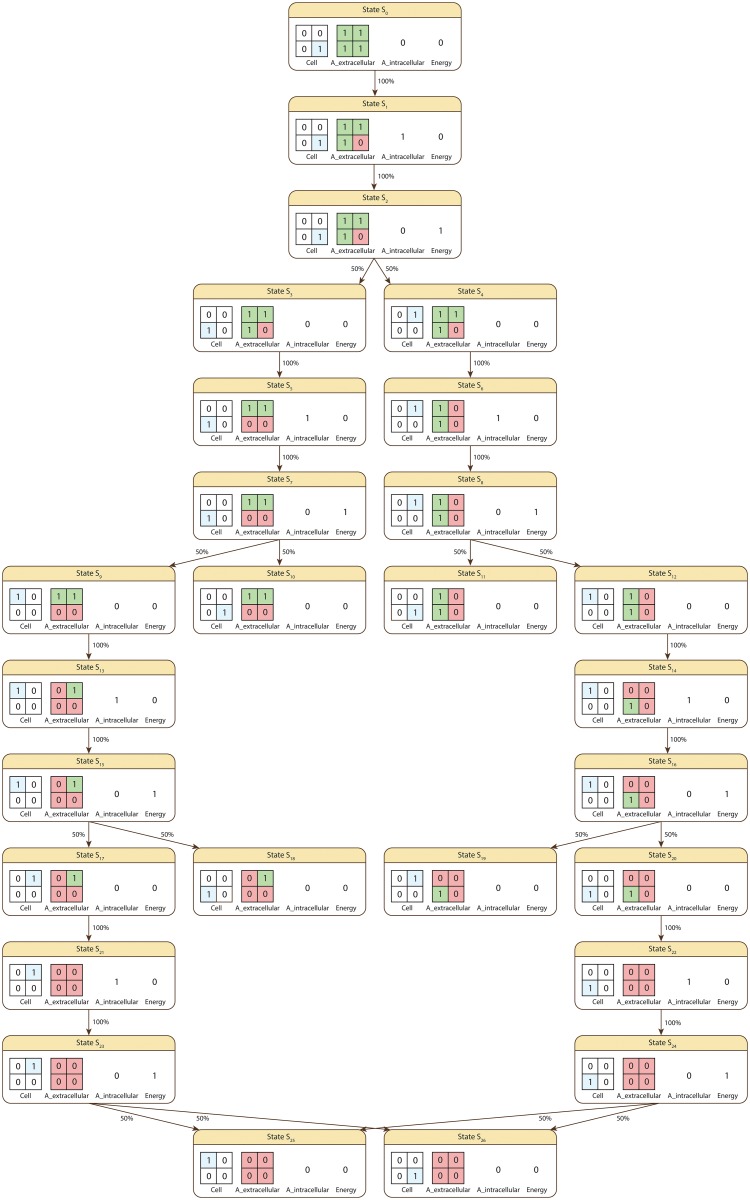
The state space of the system i.e. all possible states which can be reached from the initial state *S*_0_. *Cell* and *A*_*extracellular* are the spatial state variables representing the position of the cell, respectively distribution of nutrient *A* in the environment. *A*_*intracellular* and *Energy* represent the intracellular availability of nutrient *A*, respectively energy. The percentage associated with the arrows connecting each pair of states represents the probability of transitioning from one state to the other.

Starting from the initial state *S*_0_ the system can (in)directly transition to any of the states depicted in Fig 3.

Given that in *S*_0_ the cell has no supplies of intracellular nutrient *A* or energy, the only possible action is for it to assimilate *A* from its environment (*S*_0_ → *S*_1_, probability 100%). Since only one supply of nutrient *A* is available the only possible next action is to convert the newly gained intracellular *A* supply to energy (*S*_1_ → *S*_2_, probability 100%). Once a supply of energy is available the cell can move either above (*S*_2_ → *S*_4_) or to its left (*S*_2_ → *S*_3_). The probability of moving to either of the neighbouring positions is therefore equal to 100% / 2 = 50%. Continuing from either state *S*_3_ or *S*_4_ the cell will try to assimilate new *A* nutrient supplies, which can be converted to energy and then used to move in the environment. This process is repeated multiple times until the cell reaches a state in which it has no *A* nutrients available extracellularly/intracellularly, respectively no supplies of energy (i.e. *S*_10_, *S*_11_, *S*_18_, *S*_19_, *S*_25_, *S*_26_). In such cases the cell becomes dormant and the system reaches its final state.

**Fig 4:**
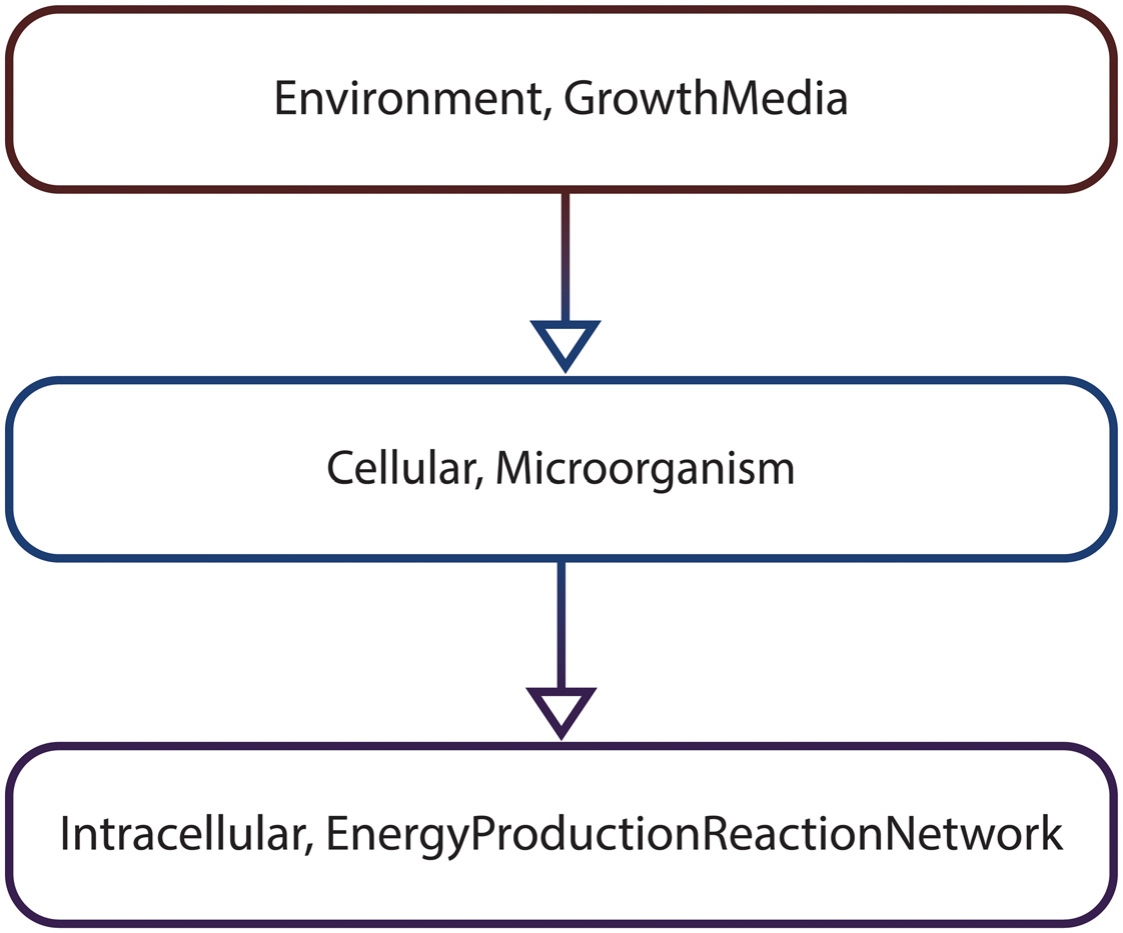
The multiscale architecture graph corresponding to the simple illustrative MSSpDES example. Each vertex in the graph (e.g. (Environment, GrowthMedia)) corresponds to a subsystem (e.g. growth media) and its associated scale (e.g. environment). Directed edges between vertices (e.g. ((Environment, GrowthMedia), (Cellular, Microorganism))) indicate how one subsystem from a coarse-grained scale (e.g. (Environment, GrowthMedia)) can be decomposed in one or multiple subsystems from more fine-grained scales (e.g. (Cellular, Microorganism)).

Using the notations above we formally define the corresponding MSSpDES model M and (state) transition probability function **P** as follows:

- M = 〈*S*, *T*, *μ*, *NSV*, *SpSV*, *NV*, *CSpV*, *MA*, *SVSS*〉, where:
  - *S* = {*S*_0_, *S*_1_, *S*_2_, *S*_3_, *S*_4_, *S*_5_, *S*_6_, *S*_7_, *S*_8_, *S*_9_, *S*_10_, *S*_11_, *S*_12_, *S*_13_, *S*_14_, *S*_15_, *S*_16_, *S*_17_, *S*_18_, *S*_19_, *S*_20_, *S*_21_, *S*_22_, *S*_23_, *S*_24_, *S*_25_, *S*_26_}.
  - *T* = ℤ_+_ is the set representing time.
  - *μ* is the function used to compute the probability associated with a set of paths *Paths*(*S*_0_) starting from *S*_0_ having a common finite prefix *σ*_*finite*_ = {*s*_0_, *s*_1_, …, *s*_*n*_}, which means that for all *σ* ∈ *Paths*(*S*_0_), $\sigma \left[i\right]=\sigma_{finite}\left[i\right]=s_{i},i=\bar{0,n}$, where *σ*[*i*] denotes the *i*-th state in *σ*. The probability value corresponding to *Paths*(*S*_0_) is computed by multiplying the probabilities of the state transitions associated with the common finite path prefix *σ*_*finite*_. For instance given the finite state sequence *σ*_*finite*_ = {*S*_0_, *S*_1_, *S*_2_, *S*_3_, *S*_5_, *S*_7_, *S*_10_}, *μ*({*σ* ∈ *Paths*(*S*_0_) | *σ*[*i*] = *σ*_*finite*_[*i*], 0 ≤ *i* ≤ 6}) = **P**(*S*_0_, *S*_1_) ⋅ **P**(*S*_1_, *S*_2_) ⋅ **P**(*S*_2_, *S*_3_) ⋅ **P**(*S*_3_, *S*_5_) ⋅ **P**(*S*_5_, *S*_7_) ⋅ **P**(*S*_7_, *S*_10_), where the probability values **P**(*S*_*i*_, *S*_*j*_) with *S*_*i*_, *S*_*j*_ ∈ *S* are recorded by the transition probability function **P** provided below.
  - *NSV* = {*A*_*intracellular*,*Energy*}, and *NV* is the function used to compute the value of *A*_*intracellular* and *Energy* in a given state of a computation path. The values of the numeric state variables for each state (e.g. *NV*(*S*_0_, *Energy*) = 0) are depicted in Fig 3 and therefore will not be explicitly restated here.
  - *SpSV* = {*Cell*, *A*_*extracellular*}, and *CSpV* = {*SpV*} is the collection containing the spatial value assignment function *SpV* used to evaluate *Cell* and *A*_*extracellular* in a given state of a computation path. The values of the spatial state variables for each state (e.g. *SpV*(*S*_0_,*Cell*) = [0, 0;0, 1]) are depicted in Fig 3 and therefore will not be explicitly restated here.
  - *MA* is the multiscale architecture graph depicted in Fig 4 encoding the hierarchical organization of the considered subsystems, namely the growth media (environment scale), the microorganism (cellular scale) and the energy production reaction network (intracellular scale).
  - *SVSS* is the state variable scale and subsystem assignment function which associates state variables to particular subsystems encoded as vertices in the *MA* graph. The values returned by *SVSS* for the considered state variables are: *SVSS*(*A*_*intracellular*) = (Intracellular, EnergyProductionReactionNetwork), *SVSS*(*Energy*) = (Intracellular, EnergyProductionReactionNetwork), *SVSS*(*Cell*) = (Cellular, Microorganism), and *SVSS*(*A*_*extracellular*) = (Environment, GrowthMedia).
- **P** is the transition probability function which records the probability of transitioning between any two states of the system *s*_*i*_, *s*_*j*_ ∈ *S*. Due to page size constraints it is not possible to represent **P** explicitly. Instead only its non-zero entries are given below: **P**(*S*_0_, *S*_1_) = 100%, **P**(*S*_1_, *S*_2_) = 100%, **P**(*S*_2_, *S*_3_) = 50%, **P**(*S*_2_, *S*_4_) = 50%, **P**(*S*_3_, *S*_5_) = 100%, **P**(*S*_4_, *S*_6_) = 100%, **P**(*S*_5_, *S*_7_) = 100%, **P**(*S*_6_, *S*_8_) = 100%, **P**(*S*_7_, *S*_9_) = 50%, **P**(*S*_7_, *S*_10_) = 50%, **P**(*S*_8_, *S*_11_) = 50%, **P**(*S*_8_, *S*_12_) = 50%, **P**(*S*_9_, *S*_13_) = 100%, **P**(*S*_12_, *S*_14_) = 100%, **P**(*S*_13_, *S*_15_) = 100%, **P**(*S*_14_, *S*_16_) = 100%, **P**(*S*_15_, *S*_17_) = 50%, **P**(*S*_15_, *S*_18_) = 50%, **P**(*S*_16_, *S*_19_) = 50%, **P**(*S*_16_, *S*_20_) = 50%, **P**(*S*_17_, *S*_21_) = 100%, **P**(*S*_20_, *S*_22_) = 100%, **P**(*S*_21_, *S*_23_) = 100%, **P**(*S*_22_, *S*_24_) = 100%, **P**(*S*_23_, *S*_25_) = 50%, **P**(*S*_23_, *S*_26_) = 50%, **P**(*S*_24_, *S*_25_) = 50%, **P**(*S*_24_, *S*_26_) = 50%.

In spite of the simplicity of the scenario described above the same model development principles apply to more complex multiscale real-life systems. However due to the inherent complexity of such systems the size of the state space is expected to be larger.

The main reason for encoding multiscale stochastic biological systems using a low-level modelling formalism such as MSSpDES is to enable our model checking approach to be employed for the general class of SDESs, which MSSpDESs extend, instead of restricting it to a particular high-level modelling formalism.

Although MSSpDES models are restricted to a two-dimensional spatial representation (see codomain of spatial value assignment functions *SpV*_*i*_ ∈ *CSpV*), extending the models from a two- to, for instance three-dimensional spatial representation, requires only replacing the codomain ${\mathbb{R}}^{m_{i}\times n_{i}}$ of each *SpV*_*i*_ ∈ *CSpV* with ${\mathbb{R}}^{m_{i}\times n_{i}\times p_{i}}$.

MSSpDESs are multiscale extensions of SSpDESs 〈*S*, *Tr*, *μ*, *NSV*, *SpSV*, *NV*, *SpV*〉, where the semantics of *S*, *μ*, *NSV*, *SpSV* and *NV* is preserved, the transition rates matrix *Tr* was replaced by the set *T* representing time and the state transition probabilities are defined by a transition probability function **P** for discrete-time systems, respectively are derived from a transition rates matrix **Q** for continuous-time systems. The single spatial value assignment function *SpV* in an SSpDES is replaced by *CSpV*, the *MA* graph is defined to explicitly encode the hierarchical representation of the systems under consideration, and *SVSS* is introduced to associate state variables with particular scales and subsystems encoded as vertices in the *MA* graph. The main advantage of defining MSSpDESs as extensions of SSpDESs is backwards compatibility. SSpDESs can be encoded as MSSpDESs where the set *T* and probability measure *μ* are defined accordingly, *CSpV* contains a single element *SpV*, and the *MA* graph contains only one vertex to which all state variables are assigned using *SVSS*. Due to this, multiple SSpDESs employing the same representation of time can be easily integrated into a single MSSpDES by defining the set *T* and probability measure *μ* accordingly, gathering all spatial value assignment functions *SpV* into a single collection, constructing a corresponding *MA* graph, mapping state variables to appropriate vertices in the graph and adding interactions between submodels.

### Multiscale spatio-temporal analysis

#### Detection and analysis of spatial entities

Let us denote execution traces (or time series data) generated by MSSpDES models as *σ* = {(*s*_0_, *t*_0_), (*s*_1_, *t*_1_), …}, where *s*_0_, *s*_1_, … represent the states of the execution trace and *t*_0_, *t*_1_, … the time durations spent in each corresponding state. Typically in case of a continuous-time representation the time durations are represented by non-negative real values *t*_0_, *t*_1_, … ∈ ℝ_+_, whereas in case of a discrete-time representation by non-negative integer values *t*_0_, *t*_1_, … ∈ ℤ_+_.

Given an execution trace *σ* = {(*s*_0_, *t*_0_), (*s*_1_, *t*_1_), …}, a numeric state variable *nsv* and a spatial state variable *spsv*, it is possible to reason about how the values of *nsv* and *spsv* change over time by evaluating them for each state in *σ* using *NV*(*s*_0_, *nsv*), *NV*(*s*_1_, *nsv*), …, respectively *SpV*(*s*_0_, *spsv*), *SpV*(*s*_1_, *spsv*), …. Although the sequence *SpV*(*s*_0_, *spsv*), *SpV*(*s*_1_, *spsv*), … describes how the entire discretised spatial domain $DSD=\mathbb{R}{}^{m_{spsv}\times n_{spsv}}$ corresponding to *spsv* changes over time, we are interested in reasoning about how emergent spatial structures, called spatial entities, identified by subsets of positions in *DSD* change over time. For instance assuming that *spsv* records the cellular density in a 2D environment *DSD* and that we would like to reason about spatial entities denoting multicellular populations, then only the subsets comprising at least *x* (e.g. *x* = 20) neighbouring positions in *DSD* having the cellular density value greater than 0 would be considered. To reason about such spatial entities there is a need for an additional processing step which automatically detects and analyses how the spatial entities change over time.

**Fig 5:**
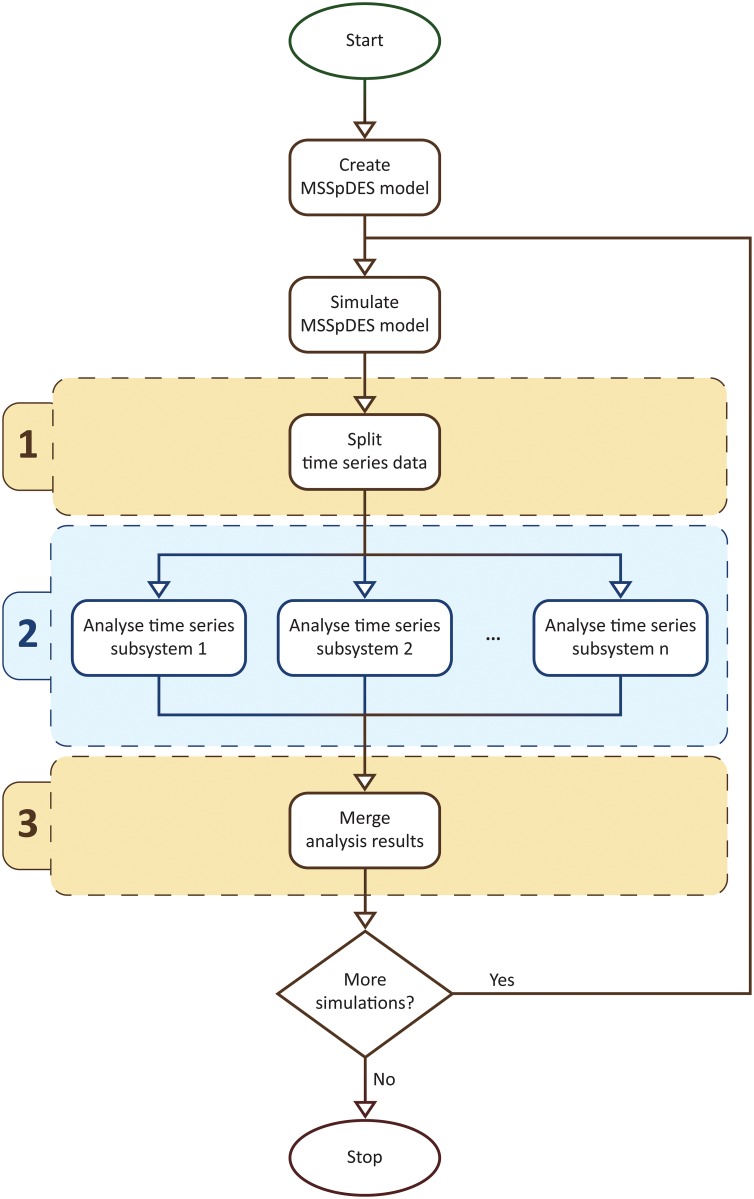
The multiscale spatio-temporal analysis workflow. An MSSpDES model of the system under consideration is constructed and simulated to generate time series data. This time series data is split up into subsets (1) such that each subset corresponds to a single subsystem and scale. The time series data subsets are passed to a uniscale spatio-temporal analysis module (2) which automatically detects, analyses and annotates spatial entities with their corresponding scale and subsystem. The results of the uniscale spatio-temporal analysis are then merged (3) such that spatial entities corresponding to the same time point are grouped together. If more simulations are required, a new time series dataset is generated, for which steps (1)–(3) are repeated.

This processing step is denoted as the multiscale spatio-temporal analysis and its associated workflow is depicted in Fig 5. The first step in the workflow is to split up the time series data corresponding to all spatial state variables such that each resulting time subseries corresponds to a single subsystem and scale. Next each time subseries is passed to a uniscale spatio-temporal analysis module which automatically detects, analyses and annotates spatial entities with their corresponding scale and subsystem. Finally, during the last step the collections of detected spatial entities are merged such that spatial entities corresponding to the same time point are grouped together.

The uniscale spatio-temporal analysis module assumes that the problem of detecting and analysing spatial entities at a given time point is transformed into an image processing problem. This transformation is possible because the spatial domain is assumed to be discretised and (the value of) each position in the discretised space can be mapped to (the intensity of) a pixel in an image. One of the main advantages of this is that existing image processing approaches for detecting and analysing objects in images can be directly reused.

We define parameterized detection and analysis modules for two generic types of spatial entities, namely *regions* and *clusters* [57].

Regions represent subsets of neighbouring positions in the discretised space (considering the Moore neighbourhood relation) with associated values (e.g. concentrations) above a user-defined threshold. For instance considering a computational model that encodes the evolution of a population of cells in a 2D environment, regions could represent patches of neighbouring cells where the cellular density is greater than a user-defined value. More formally a region *R* is defined with respect to a state *s* and spatial state variable *spsv* as a subset ${\left\{0,1\right\}}^{m_{spsv}\times n_{spsv}}$ (i.e. positions of the discretised space included in *R* are marked with 1, all others with 0) of neighbouring positions in *Sp* *V(s,spsv)* such that for all positions of the discretised space (*i*, *j*) ∈ *R* marked with 1, the corresponding value *Sp* *V(s, spsv)*[*i*, *j*] ≥ *THRESHOLD*, and the number of positions included in *R* is greater than *ϵ*_*size*_, where *THRESHOLD* ∈ ℝ, *ϵ*_*size*_ ∈ ℕ are user-defined parameters. The module for detecting and analysing regions is an implementation of Algorithm 1 in [57] using image processing functions from the open source Computer Vision library OpenCV [62].

Conversely clusters represent subsets of neighbouring regions in the discretised space where the maximum distance between two neighbouring regions is bounded above by a user-defined threshold. For instance considering again the computational model encoding the evolution of a population of cells, clusters could represent groups of patches of cells where the distance between neighbouring patches is less or equal to a user-defined threshold value. Clusters are computed using an improved version of the DBSCAN algorithm [63]. The output of this algorithm depends on the given set of regions *REG*, the pseudometric *d* used to compute the distance between any two regions in *REG*, the maximum distance *ϵ*_*distance*_ between two neighbouring regions, and the minimum number of regions *ϵ*_*size*_ neighbouring a *core* region, where a region is denoted as *core* if its number of neighbouring regions is greater or equal to *ϵ*_*size*_. The pseudometric *d* considered here is defined with respect to a set of regions *REG*, *d*: *REG* × *REG* → ℝ_+_, $d\left(A,B\right)=\sqrt{{\left(x_{B}-x_{A}\right)}^{2}+{\left(y_{B}-y_{A}\right)}^{2}}$, where (*x*_*A*_, *y*_*A*_) and (*x*_*B*_, *y*_*B*_) are the centroids of regions *A*, respectively *B*. Moreover two regions *REG*_1_, *REG*_*n*_ ∈ *REG* are called *density-reachable* if there exists a sequence of regions *REG*_1_, *REG*_2_, …, *REG*_*n*_ ∈ *REG*, where *i* ≥ 1 and *n* ≥ 2 such that for all *i* < *n*, *REG*_*i*_ is a *core* region, and *REG*_*i*+1_ is a neighbour of *REG*_*i*_. Using the notations above a cluster *C* is defined as a maximal subset ${\left\{0,1\right\}}^{m_{1}\times n_{1}}\times {\left\{0,1\right\}}^{m_{2}\times n_{2}}\times ...\times {\left\{0,1\right\}}^{m_{p}\times n_{p}}$ (i.e. regions’ positions included in *C* are marked with 1, all others with 0) of the given set of regions *REG* = {*REG*_1_, *REG*_2_, …, *REG*_*p*_} such that all regions in *C* are *density-reachable* from an arbitrary *core* region of *C* [63].

Each detected region/cluster is characterized by a set of general quantitative spatial measures that enable describing how the spatial entity changes over time. A description of the set of spatial measures considered is given in Table 1.

**Table 1:** Description of the spatial measures considered.

Name	Values	Description
clusteredness	[0, 1]	Indicates if regions contain holes (clusteredness <1) or not (clusteredness = 1), respectively measures if the average distance between all positions considered in a cluster is small (clusteredness →1) or large (clusteredness →0).
density	[0, 1]	Computes the average value associated with the discretised spatial positions defining a region/cluster.
area	ℝ_+_	Represents the number of positions in the discretised space associated with a region/cluster.
perimeter	ℝ_+_	Represents the length of the outer contour of a region, respectively the convex hull of a cluster.
distance from the origin	ℝ_+_	Computes the minimum distance between the outer contour of a region, respectively the convex hull of a cluster, and the centre point of the discretised spatial domain.
angle	[0, 360] (degrees)	Determined by the lines that pass through the discretised spatial domain’s centre point and are tangent to a region’s outer contour, respectively cluster’s convex hull.
triangle/rectangle/circle measure	[0, 1]	Indicates if the shape of the region’s outer contour, respectively cluster’s convex hull, is similar to a triangle/rectangle/circle (triangle/rectangle/circle measure →1) or not (triangle/rectangle/circle measure →0).
centroid Ox/Oy coordinate	ℝ_+_	Represents the Ox/Oy coordinate of the geometric centre of the region’s outer contour, respectively cluster’s convex hull.

Each spatial measure considered has a name (column “Name”), an associated range of valid values (column “Values”) and a corresponding description (column “Description”). In case of spatial measures which have similar semantics the table rows have been merged and the spatial measure names are separated by the “/” symbol (see last two table rows).

The spatial entity types and measures were chosen relative to the case studies considered here. Therefore depending on case study specific requirements different sets of spatial entity types and/or measures may need to be employed. For instance, extending the spatial representation from two to three dimensions requires employing appropriate types of spatial entities (e.g. 3D structure) and measures (e.g. volume), and updating the multiscale spatio-temporal analysis module (implementation) accordingly. Moreover (the value corresponding to) each position in the discretised space is mapped to (the intensity of) a voxel, rather than a pixel in an image. The model checking approach is adapted automatically to different spatial entity types and/or measures using the spatio-temporal meta model checking concept described later.

The output of the multiscale spatio-temporal analysis is time series data describing how the values of the spatial measures considered change over time for each detected spatial entity, scale and subsystem.

#### Multiscale Spatial Temporal Markup Language

The MSSpDES model simulation results are represented by time series data produced by the multiscale spatio-temporal analysis and time series data describing the evolution over time of numeric state variables values.

To represent these model simulation results in a uniform manner which facilitates exchange of data sets and integration of software tools a corresponding standard data representation format is required. To the best of our knowledge such a standard data representation format does not exist.

One of the main requirements for the data representation format is that it supports recording different numbers of values at different time points because the collection of (emergent) spatial entities considered could potentially change over time. Traditional tabular (e.g. csv) representation formats are not suitable because they assume that the number of recorded values (or columns) is constant throughout the entire time series. Moreover defining a representation format similar to csv that does not annotate numeric values with their meaning could be potentially difficult to interpret.

For portability, structuring and readability purposes an eXtensible Markup Language (XML) based standard representation format is defined called *Multiscale Spatial Temporal Markup Language* (MSTML). The rules and constraints for the structure of MSTML files are formalised in XML Schema Definition (xsd) files. The latest version of the MSTML format is made available at http://mule.modelchecking.org/standards, a description of the format is given in S3 Text, and an example of an MSTML formatted file is depicted in Listing 1.

**Listing 1.** An example MSTML file recording multiscale spatio-temporal time series data.

1 <?**xml version**=“1.0” encoding=“utf −8”?>

2 <experiment>

3  <timepoint value=“1”>

4   <spatialEntity spatialType=“cluster” scaleAndSubsystem=“Organ.Liver”>

5    <clusteredness>0.01</clusteredness>

6    <density>0.4</density>

7    <area>15</area>

8    <perimeter>28</perimeter>

9    <distanceFromOrigin>81</distanceFromOrigin>

10    <angle>10.5</angle>

11    <triangleMeasure>0.5</triangleMeasure>

12    <rectangleMeasure>1.0</rectangleMeasure>

13    <circleMeasure>0.1</circleMeasure>

14    <centroidX>703.4999</centroidX>

15    <centroidY>118.087</centroidY>

16   </spatialEntity>

17   <numericStateVariable scaleAndSubsystem=“Cellular.Hepatocyte”>

18    <name>dysfunction</name>

19    <value>0.1</value>

20   </numericStateVariable>

21  </timepoint>

22  …

23 </experiment>

For model checking purposes the number of MSTML files *#MSTML* generated for an MSSpDES model assuming fixed parameter values varies depending if the model is deterministic (*#MSTML* = 1) or stochastic (*#MSTML* ≥ 1), and if the required level of confidence for the model checking result is high (e.g. 99%) or low (e.g. 70%).

To determine the correctness of a model the model checker verifies if its behaviour captured by a corresponding set of MSTML files conforms to a given formal specification.

### Formal specification

The temporal logic employed to write the formal specification needs to enable reasoning about how values of numeric state variables and/or spatial measures, which are the state variables considered, are expected to change over time and multiple scales.

To the best of our knowledge the only formal language for reasoning about numeric and spatial properties corresponding to computational models of biological systems is called Bounded Linear Spatial Temporal Logic (BLSTL), which we have previously introduced in [57]. One of the main limitations of BLSTL is that it does not enable different scales to be explicitly distinguished. Therefore it is not possible to relate how changes at one scale reflect at another scale and vice versa.

#### Bounded Linear Multiscale Spatial Temporal Logic

To address the issue of relating changes between scales we define the *Bounded Linear Multiscale Spatial Temporal Logic* (BLMSTL) which enables explicitly distinguishing between state variables corresponding to different scales and subsystems. Throughout it is assumed that the scales and subsystems considered are the same as the ones defined in the *MA* graph of the corresponding MSSpDES model. Although MSSpDESs can be employed to represent both discrete- and continuous-time stochastic discrete-event systems, the semantics of a temporal logic usually varies with the considered representation of time. Therefore in this paper we restrict the semantics of BLMSTL to a continuous-time representation (similarly to CSL [64] and in contrast to BLSTL). However adapting BLMSTL to a discrete-time representation requires changing only the semantics of the time dependent operators, whereas the definition of all other atomic propositions (related to different scales and subsystems, numeric state variables, and spatial entities) is preserved.

BLMSTL enables reasoning about how collections, or more formally bags, of spatial measures values from one time point, and collections of numeric state variables and spatial measures values corresponding to multiple time points change over time using statistical functions. Transfer relations between state variables from the same and/or different scales are encoded using standard arithmetic functions. An informal natural language description of the most relevant BLMSTL features is given below; see S4 Text for a formal definition of the BLMSTL syntax and semantics.

Similarly to BLSTL, BLMSTL employs temporal and Boolean operators for describing how a system changes over time, respectively for composing simple logic statements into more complex ones. BLMSTL atomic propositions enable describing relations between numeric state variables and/or spatial measures associated to subsets of spatial entities.

Numeric state variables are specified by their name (e.g. heartBeat) and their associated scale and subsystem (e.g. (organ, heart)); the corresponding BLMSTL notation for specifying scales and subsystems is scale.subsystem (e.g. organ.heart). Conversely spatial measures associated with subsets of spatial entities are specified by their spatial measure type (e.g. area), associated spatial entity type (e.g. regions) and their corresponding scale and subsystem. Similarly to MSTML the sets of spatial entity types and spatial measures considered are *SET*_*considered*_ = {clusters, regions}, respectively *SM*_*considered*_ = {clusteredness, density, area, perimeter, distanceFromOrigin, angle, triangleMeasure, rectangleMeasure, circleMeasure, centroidX, centroidY}.

Instead of considering all spatial entities of a given type it is possible to select only a subset of spatial entities by imposing constraints over the spatial measure values (e.g. spatial entities with area > 10), by using subset operators \ (difference), ∩ (intersection) and ∪ (union), or specifying one or multiple scales and subsystems using the partial orders < and ≤ defined over the set of vertices *V*_*MA*_ (e.g. spatial entities whose corresponding scale and subsystem < (organ, heart)).

The resulting collection of spatial measures values corresponding to multiple spatial entities (e.g. value of the area for all detected spatial entities) can be described using unary (e.g. mean), binary (e.g. covariance) or binary quantile (e.g. percentile) statistical functions. These statistical functions can be additionally employed to reason about collections of numeric state variables and spatial measures values corresponding to multiple time points (e.g. the value of numeric state variable *X* for all time points in the time interval [0, 100]). By considering different numbers of time points for different state variables it is possible, for instance, to describe how values corresponding to one time point (and a coarse-grained scale) relate to other values corresponding to multiple time points (and a fine-grained scale), or vice versa.

Transfer functions defined over state variables from different scales can be encoded using unary (e.g. square root) and binary (e.g. add) arithmetic functions. For instance if the value of a state variable *sv*_*cg*_ from a coarse-grained scale is equal to the arithmetic mean of four state variables *sv*_*fg*_1__, *sv*_*fg*_2__, *sv*_*fg*_3__, *sv*_*fg*_4__ from a more fine-grained scale, this can be written as *sv*_*cg*_ = (*sv*_*fg*_1__+*sv*_*fg*_2__+*sv*_*fg*_3__+*sv*_*fg*_4__)/4; in BLMSTL “+” and “/” would be replaced by the arithmetic functions *add*, respectively *div*.

Illustrative examples of statements written both in natural language and BLMSTL are given below. For simplicity the number of scales and subsystems explicitly specified is two in all examples.

- **Natural language:** Always during the time interval [0, 95] if the concentration of EGFR (corresponding to scale and subsystem (Intracellular, RasERKPathway)) increases over 20 M, then the cancerous cell (corresponding to scale and subsystem (Cellular, Cancerous)) will divide i.e. the cell count will increase.
  **BLMSTL:** *G*[0, 95] (({*EGFR*}(*scaleAndSubsystem* =
  *Intracellular.RasERKPathway*) > 20) ⇒
  (*d*(*count*(*density*(*filter*(*regions*, *scaleAndSubsystem* =
  *Cellular.Cancerous*)))) > 0)).
- **Natural language:** If the concentration of drug *X* (corresponding to scale and subsystem (Organism, Human)) eventually increases during time interval [5, 10], then the area of the aorta cross section (corresponding to scale and subsystem (OrganSystem, Aorta)) will be larger during time interval [10, 30] than [0, 10].
  **BLMSTL:** (*F* [5, 10] *d*({*X*}(*scaleAndSubsystem* = *Organism.Human*)) >0) ⇒
  (*min*([10, 30] *min*(*area*(*filter*(*regions*, *scaleAndSubsystem* =
  *OrganSystem.Aorta*)))) >
  *max*([0, 10] *max*(*area*(*filter*(*regions*, *scaleAndSubsystem* =
  *OrganSystem.Aorta*))))).
- **Natural language:** Always during the time interval [0, 100] the liver dysfunction measure (corresponding to scale and subsystem (Organ, Liver)) is equal to the average density of damaged liver tissues (corresponding to scales and subsystems ≤ (Tissue, DamagedLiverTissue)). The assumption made here is that the density value represents the degree of damage suffered by the liver tissue.
  **BLMSTL:***G* [0, 100] ({*LiverDysfunction*} (*scaleAndSubsystem* =
  *Organ.Liver*) = *avg*(*density*(*filter*(*regions*, *scaleAndSubsystem* ≤
  *Tissue.DamagedLiverTissue*)))).

To enable the explicit encoding of the probability with which a BLMSTL statement is expected to hold, a probabilistic extension of BLMSTL called Probabilistic Bounded Linear Multiscale Spatial Temporal Logic is defined.

#### Probabilistic Bounded Linear Multiscale Spatial Temporal Logic

A *Probabilistic Bounded Linear Multiscale Spatial Temporal Logic* (PBLMSTL) property *ϕ* is a logic property of the form *P*_⋈*θ*_[*ψ*] where ⋈ ∈ {<, < =, > =, >}, *θ* ∈ (0, 1) and *ψ* is a BLMSTL property.

An illustrative example of a natural language probabilistic statement mapped into PBLMSTL is given below:

**Natural language:** The probability is greater than 0.99 that always during the time interval [0, 95] if the concentration of EGFR (corresponding to scale and subsystem (Intracellular, RasERKPathway)) increases over 20 M, then the cancerous cell (corresponding to scale and subsystem (Cellular, Cancerous)) will divide i.e. the cell count will increase.
  **PBLMSTL:** *P* > 0.99 [*G*[0, 95] (({*EGFR*}(*scaleAndSubsystem* = *Intracellular.RasERKPathway*) > 20) ⇒
  (*d*(*count*(*density*(*filter*(*regions*, *scaleAndSubsystem* =
  *Cellular.Cancerous*)))) > 0))].

A PBLMSTL property *ϕ* ≡ *P*_⋈*θ*_[*ψ*] holds for an MSSpDES M if and only if the probability of *ψ* to hold for a model simulation is ⋈*θ*. Therefore in order to determine the truth value of a PBLMSTL property *ϕ* the likelihood of *ψ* being true needs to be computed.

### Model checking

The *multiscale spatio-temporal model checking problem* is to automatically verify if an MSSpDES M satisfies a PBLMSTL property *ϕ*.

In order to solve the model checking problem only approximate probabilistic model checking approaches are considered throughout. As illustrated in Table 2 the approaches considered are either Bayesian or frequentist, and estimate or hypothesis testing based; a brief description of each approach was given in our previous work [57] and will not be restated here.

**Table 2:** Considered approximate probabilistic model checking approaches.

Name	Type	Input	Description	Sample size	Ref.
Chernoff-Hoeffding bounds based	FE	*ϵ*, *δ*	The absolute difference between the estimated *p* and true *p*′ probability of *ψ* to hold is greater than *ϵ* with probability less than *δ* (i.e. *P*[|*p* − *p*′| > *ϵ*] < *δ*).	$n=\frac{4}{\epsilon^{2}}\text{log}(\frac{2}{\delta })$	[65]
Improved frequentist statistical hypothesis testing	FH	*α*, *β*	Wald’s sequential probability ratio test [66] is employed to decide if the null hypothesis *H*_0_ is rejected in favour of the alternative hypothesis *H*_1_ considering the upper bounds on the probability of type I and type II errors *α*, respectively *β*.	The value of *n* is determined during the execution of the model checking approach considering *α*, *β* and the number and order of MSTML files against which *ψ* evaluates true; see ([67] [p. 21]) for an approach on how to compute an upper bound for *n*.	[59, 68]
Probabilistic black-box	FH	-	The p-value associated with the null and alternative hypotheses *H*_0_, respectively *H*_1_ is computed after evaluating the *n* MSTML files against *ψ*. The hypothesis with the lowest corresponding p-value holds.	*n* > 0	[69, 70]
Bayesian mean and variance based	BE	*α*, *β*, *T*	The probability *ρ* and variance *ν* of *ψ* to hold are estimated considering the given MSTML files and the Beta prior parameters *α* and *β*. New MSTML files are evaluated against *ψ* until the condition *ν* < *T* holds.	The value of *n* is determined during the execution of the model checking approach considering *α*, *β*, *T* and the number and order of MSTML files against which *ψ* evaluates true.	[71]
Bayesian statistical hypothesis testing	BH	*α*, *β*, *T*	A measure B of confidence in the null hypothesis *H*_0_ relative to the alternative hypothesis *H*_1_ is computed considering the Beta prior parameters *α* and *β*. New MSTML files are evaluated against *ψ* until either B>T or B<1/T.	The value of *n* is determined during the execution of the model checking approach considering *α*, *β*, *T* and the number and order of MSTML files against which *ψ* evaluates true.	[72, 73]

Each table body row corresponds to a different approximate probabilistic model checking approach. The columns from left to right record the name, type (i.e. F—Frequentist, B—Bayesian, E—Estimate, H—Hypothesis testing), input parameters (excluding *ϕ* and MSTML files), description, sample size (i.e. *n*) and reference corresponding to a model checking approach. The null (i.e. *H*_0_) and alternative (i.e. *H*_1_) hypotheses represent *ϕ* (e.g. *P*_>*θ*_[*ψ*]), respectively the opposite of *ϕ* (e.g. *P*_≤*θ*_[*ψ*]). Bayesian methods consider prior knowledge when deciding if a logic property holds. Conversely frequentist approaches assume that no prior knowledge is available. All methods except probabilistic black-box take as input a user-defined upper bound on the approximation error. They request additional model simulations until the result is sufficiently accurate. Conversely probabilistic black-box model checking takes a fixed number of model simulations as input and computes a p-value as the confidence measure of the result.

By means of approximate probabilistic model checking approaches the verification of a PBLMSTL specification against an MSSpDES model is guaranteed to terminate. Therefore the corresponding multiscale spatio-temporal model checking problem is well-defined; see S5 Text for a formal proof. Intuitively the main idea behind the proof is to show that in order to verify an MSSpDES model the number of required model simulations is finite, and that the number of time points considered for each model simulation is bounded. Therefore the PBLMSTL specification is evaluated against a finite number of time points and model simulations, which can be done in a finite number of steps.

### Spatio-temporal meta model checking

One of the main limitations of our methodology, as described up to this point, is that the evolution over time of spatial properties can be described only with respect to the predefined collections of spatial entity types *SET*_*considered*_ = {clusters, regions} and spatial measures *SM*_*considered*_ = {clusteredness, density, area, perimeter, distanceFromOrigin, angle, triangleMeasure, rectangleMeasure, circleMeasure, centroidX, centroidY}.

In order to overcome this limitation and enable automatically reconfiguring the methodology according to case study specific spatial entity types and measures, we define a generalized version of the multiscale spatio-temporal model checking methodology called multiscale spatio-temporal *meta* model checking in which *SET*_*considered*_ and *SM*_*considered*_ are replaced with meta collections of spatial entity types *SET*, and spatial measures *SM*, defined as follows:

- $\begin{array}{ll} SET= & \{sety\;|\;sety\;\text{is}\;\text{a}\;\text{spatial}\;\text{entity}\;\text{type}\;\text{for}\;\text{which}\;\text{there}\\ & \text{exists}\;\text{a}\;\text{corresponding}\;\text{spatial}\;\text{detection}\;\text{mechanism}\;f_{sety},\\ & f_{sety}:\;SpSV^{p}\to {\left\{0,1\right\}}^{m_{1}\times n_{1}}\times {\left\{0,1\right\}}^{m_{2}\times n_{2}}\times \ldots \times {\left\{0,1\right\}}^{m_{p}\times n_{p}},\\ & \text{which detects sets of spatial entities }SE\text{ of type }sety\text{ in the}\\ & \text{discretised spatial domain}\}. \end{array}$
  Considering the spatial state variable tuples *spsvt* ∈ *SpSV*^*p*^, *f*_*sety*_ computes which positions of the discretised space are occupied (1) by spatial entities or not (0); see [57] for examples of spatial detection mechanisms corresponding to the spatial entity types *clusters* and *regions*.
- *SM* = {*sm* | *sm* is a spatial measure, *sm*: *SE* → *SMV* ⊆ ℝ, where *SE* is a set of spatial entities and *SMV* is the corresponding domain of valid spatial measure values}; similarly see [57] for examples of spatial measures corresponding to the spatial entity types *clusters* and *regions*.

These collections are called meta because they provide only a description of the conditions which should hold for each spatial entity type and spatial measure but do not explicitly define instances thereof.

The multiscale spatio-temporal meta model checking methodology enables the creation of different multiscale spatio-temporal model checking methodology instances by replacing *SET* and *SM* with case study specific collections of spatial entity types and spatial measures. These instances can then be used to verify corresponding MSSpDES models. For instance, in order to verify computational models considering a 3D representation of space a corresponding model checking methodology instance could be created that replaces *SET* and *SM* with *SET*_*3D*_ = {*cuboid*, *cylinder*, *sphere*} and *SM*_*3D*_ = {*volume*, *centroidX*, *centroidY*, *centroidZ*}.

**Fig 6:**
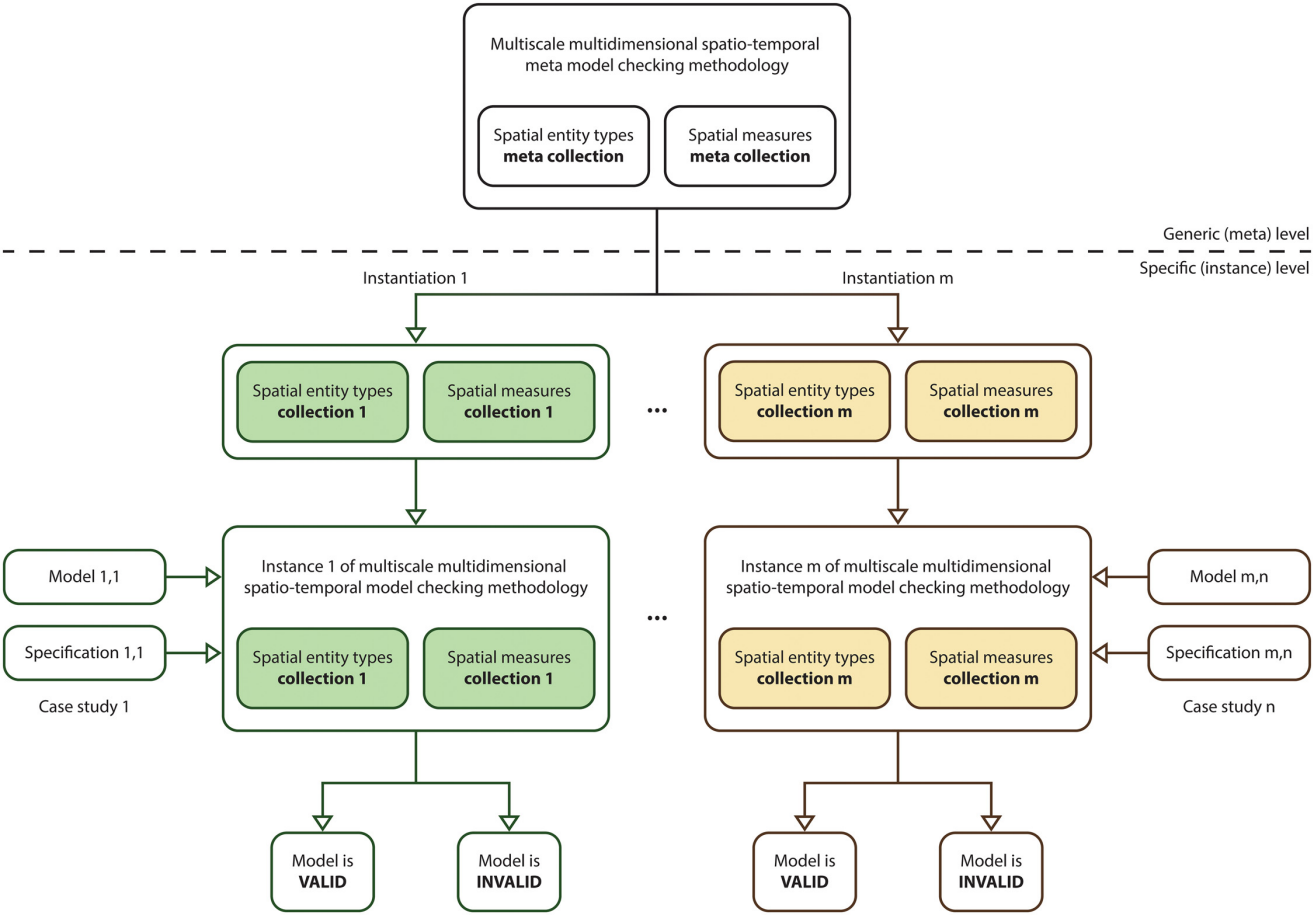
Workflow for creating multiscale spatio-temporal model checking methodology instances. The workflow comprises two levels, the upper generic (meta) level, and the lower specific (instance) level. The upper level comprises the multiscale spatio-temporal meta model checking methodology. Conversely the lower level consists of the specific collections of spatial entity types and measures employed to create multiscale spatio-temporal model checking methodology instances. For each considered pair (e.g. m) of spatial entity types and spatial measures collections a corresponding multiscale model checking methodology instance is created. The resulting methodology instances (e.g. m) can then be employed for various case studies (e.g. n) to decide if computational models (e.g. m,n) are correct relative to corresponding formal specifications (e.g. m,n) or not. Rounded rectangles and arrows having the same border/line colour correspond to the same collections of spatial entity types and spatial measures.

A graphical description of the workflow employed to create multiscale spatio-temporal model checking methodology instances is given in Fig 6. For simplicity a single multiscale model checking methodology instance is considered throughout this paper corresponding to the collections of spatial entity types and measures *SET*_*considered*_, respectively *SM*_*considered*_.

Whenever creating new multiscale model checking methodology instances there is an additional need to define corresponding image processing functions for automatically detecting and analysing spatial entities in time series data. However such functions can often be defined based on existing approaches from the image processing literature.

Finally following on from S5 Text, when verifying an MSSpDES model relative to a formal PBLMSTL specification, the number of required model simulations and the number of required state transitions for each model simulation do not depend directly on the considered collections of spatial entity types and spatial measures. Therefore regardless of the considered instances of *SET* and *SM* the multiscale spatio-temporal model checking problem is well-defined.

### Implementation

The multiscale spatio-temporal meta model checking approach was implemented in the model checking software Mule which enables automatically verifying multilevel computational models of biological systems relative to formal specifications; the model checker name is a concatenation of the first and last two letters in the word “Multiscale”. For efficiency purposes Mule was implemented in C++ and supports all approximate probabilistic model checking approaches described in Table 2.

Depending on the approximate probabilistic model checking approach employed the number of MSTML files required to verify if the computational model is valid relative to a PBLMSTL specification is computed differently. In case of Chernoff-Hoeffding bounds based and probabilistic black-box model checking approaches the number of required MSTML files can be computed before running Mule (i.e. statically). Conversely in case of the improved frequentist and Bayesian statistical hypothesis testing, and Bayesian mean and variance based model checking approaches the number of required MSTML files is determined only during the execution of Mule (i.e. dynamically). To support generating MSTML files on-demand Mule can take as input the path to a script (in our case Bash script) that simulates a computational model and stores the resulting output in MSTML files; run Mule with the command line argument —help for more execution details.

**Fig 7:**
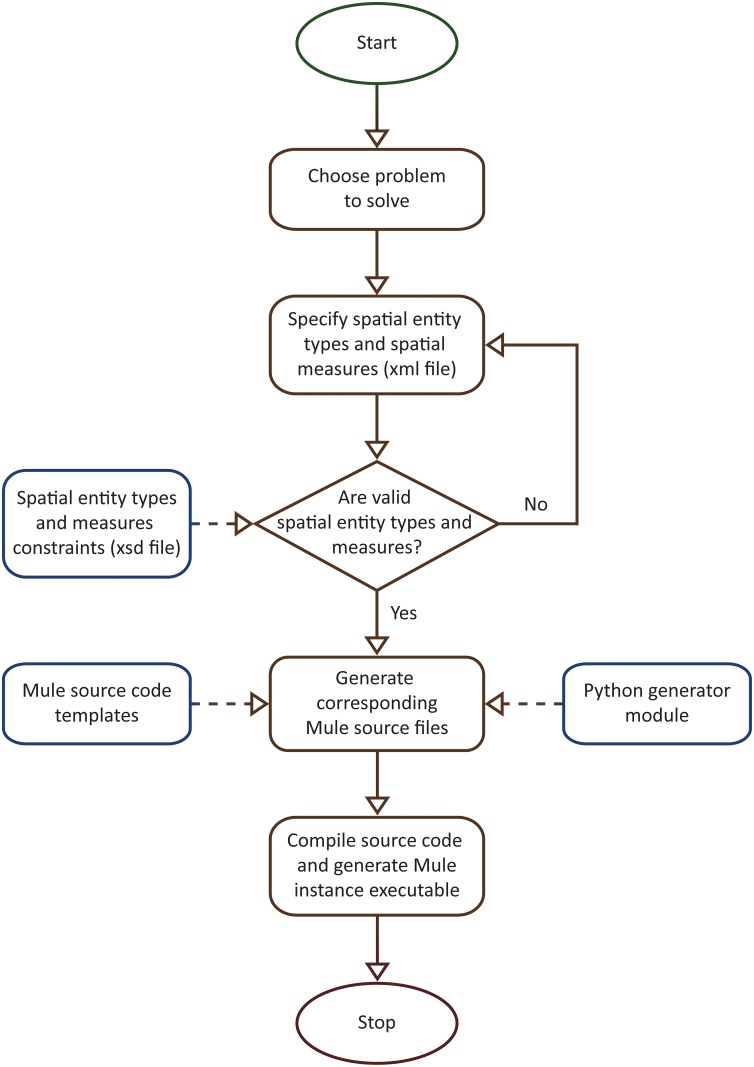
Implementation of workflow for generating multiscale spatio-temporal model checker instances according to user-defined spatial entity types and spatial measures. Starting from the problem one tries to solve, an xml file is created describing the collections of spatial entity types and spatial measures of interest. These collections are then verified with respect to relevant constraints captured by an xsd file; see http://mule.modelchecking.org/standards for the latest version of the xsd file. If the xml file verification fails then the specification of the spatial entity types and measures needs to be updated accordingly. Otherwise the xml file is employed by a C++ source code generator/translator written in Python to generate the corresponding Mule source files based on a set of predefined templates. The source files are compiled to produce an executable version of the corresponding Mule instance. This instance can then be employed to verify corresponding computational models.

The workflow for generating multiscale spatio-temporal model checker instances was implemented as described in Fig 7. The main idea behind the implementation is to use two instead of one compilation (or translation) steps. The first compilation step takes a description of the spatial entity types and measures as input and produces C++ source code as output. The second compilation step translates the generated C++ source code in binary (i.e. executable) format. Conceptually this approach is called “meta” because Mule is an abstract multiscale spatio-temporal (meta) model checker that can be instantiated according to case study specific spatial entity types and measures. From a practical point of view the user modifies only the description of the spatial entity types and measures, while the source code and the corresponding executables are automatically generated for him/her.

The main advantage of the workflow depicted in Fig 7 is that it enables the considered spatial entity types and measures to be compiled into the model checking executable instead of being (dynamically) loaded at runtime, which could negatively impact the model checker performance.

Mule was implemented as an offline model checker and takes as input model simulation traces rather than the computational models used to generate them. Using trace analysis each model simulation trace is evaluated against the PBLMSTL specification. The trace analysis results corresponding to multiple model simulation traces are used by the employed model checking approach to determine if the PBLMSTL specification holds for the model.

The main advantage of implementing Mule as an offline model checker is that it is decoupled from the specific modelling formalisms employed to encode the computational models. Consequently Mule can be employed to verify computational models encoded using various modelling formalisms provided that the corresponding computational models satisfy the constraints of an MSSpDES model without requiring the explicit translation of the computational models to MSSpDES. In addition given that Mule takes simulation traces (i.e. time series data) as input it can be employed to evaluate PBLMSTL specifications both against time series data generated *in silico* or recorded *in vitro*. Conversely the main disadvantages of Mule are that the computational models need to be constructed and simulated using external tools, and the model simulation output needs to be stored in or translated to csv format. To generate model simulations on demand Mule needs to be able to execute the model simulator from the command line.

In contrast to Mule inline approximate probabilistic model checkers (e.g. COSMOS [74], PLASMA [75], PRISM [76], UPPAAL-SMC [77], Ymer [78]) are integrated modelling and verification environments that can be employed not only to verify, but also to construct and simulate computational models. In addition inline model checkers are usually more efficient than their offline counterparts, because model simulations can be generated on-demand, in-memory and potentially stopped early (i.e. as soon as the considered logic statement is accepted/rejected). However inline model checkers typically require explicitly encoding computational models in the model checker specific modelling formalism, and they can not be employed to evaluate formal specifications against time series data recorded *in vitro*.

Both the source code and the executable corresponding to the Mule instance employed throughout this paper are made freely available online at http://mule.modelchecking.org; this Mule instance is defined with respect to the collection of spatial entity types *SET*_*considered*_ and spatial measures *SM*_*considered*_. Moreover a corresponding Docker image has been created providing a self-contained environment for executing/updating model checker instances which can be run on all major operating systems without additional setup (except installing the freely available software Docker).

## Results

We illustrate the applicability of the model checker based on four multiscale systems biology case studies published in the literature. The case studies were chosen such that the corresponding computational models are of different types (i.e. deterministic/hybrid/stochastic), span different levels of organization (e.g. cellular/organ) and are encoded using different modelling formalisms (e.g. ordinary differential equations/cellular automata) and software (e.g. Morpheus/NetLogo); see Table 3 for a brief comparison of the multilevel computational models considered.

**Table 3:** Considered multilevel systems biology computational models against which the proposed model checking methodology and implementation were validated.

	M1	M2	M3	M4
**Description**	Rat cardiovascular system dynamics	Uterine contractions of labour	*Xenopus laevis* cell cycle	Acute inflammation of the gut and lung
**Model type**	Deterministic	Deterministic	Hybrid	Stochastic
**Modelling formalism(s)**	Ordinary differential equations (ODE)	Cellular automata (CA)	ODEs + Cellular Potts model (CPM)	Agent based modelling (ABM)
**Modelling software**	JSim	Mathematica	Morpheus	NetLogo
**Explicit spatial representation**	N	Y	Y	Y
**Levels of organization**	Cellular + Organ system	Cellular + Tissue	Intracellular + Cellular	Cellular + Tissue + Organ
**Case study reference**	[13]	[14]	[15]	[16]
**Model download link**	http://virtualrat.org/sites/default/files/downloads/Workflow_Model_Files_12April2012.zip	http://s3-eu-west-1.amazonaws.com/files.figshare.com/1720626/Supporting_Information_S1	http://imc.zih.tu-dresden.de/wiki/morpheus/doku.php?id=examples:multiscale#odes_in_cpm_cellscell_cycle_and_proliferation	http://bionetgen.org/SCAI-wiki/images/7/7d/GutLungAxis2.1.nlogo

Each model (M1–M4) has an associated description and type (i.e. deterministic, stochastic or hybrid), was encoded using specific modelling formalisms and software, represents space explicitly or not (Y—Yes, N—No), spans different levels of organization, and has a corresponding reference paper and download link.

Since Mule is implemented as an offline model checker and all approximate probabilistic model checking algorithms employed here (see Table 2) are defined relative to simulation traces, the computational models M1–M4 were not explicitly translated to an MSSpDES representation. Instead the computational models encoded using high-level modelling formalisms were simulated and the simulation output was stored in MSTML files. These MSTML files were then provided as input to the model checker Mule. There are two main reasons for employing the computational models encoded in high-level modelling formalisms (as developed by their original authors) instead of MSSpDES. First of all simulating an MSSpDES computational model on a computer requires defining an MSSpDES operational semantics, which was not given here. Secondly approximations inherent to the translation of computational models between different modelling formalisms could potentially impact the outcome of the model checker execution.

In case of the deterministic continuous-state computational model M1 an alternative approach, which is not considered here, would have been to translate M1 into a stochastic discrete-state computational model. Using the approach described by Wilkinson [79] and under the assumption that the volume of the media containing the species in the model is known, concentrations can be converted into discrete numbers of molecules, and deterministic into stochastic kinetic rate constants. The main reason for not translating M1 into a stochastic model is that we want to illustrate that Mule can be employed to verify existing deterministic continuous-state computational models relative to PBLMSTL specifications without the need to initially alter the models. The probability of a PBLMSTL specification to hold for the deterministic continuous-state model M1 is either 1 (i.e. true) or 0 (i.e. false).

The natural language and corresponding formal specifications, against which the models were verified, have been derived from the original papers introducing the case studies. Quotes from the original papers have been employed to create *initial* natural language statements describing the expected system behaviour. The initial natural language statements were then rephrased to match the constructs and structure typical to formal PBLMSTL statements; the resulting statements are called *rephrased* natural language statements. Finally the rephrased natural language statements were manually mapped into corresponding PBLMSTL statements. Where insufficient information was available (e.g. probabilities) the numeric values employed in the formal specification are quantitative approximations of the corresponding natural language descriptions (e.g. with high probability ⇒ 0.9). The main purpose of the PBLMSTL statements considered is to illustrate the expressivity of the methodology and not to predict previously unknown biologically relevant properties. For reproducibility purposes the mapping between quotes from the original papers, derived natural language statements and corresponding PBLMSTL specifications is documented in the supplementary materials.

The model checking approach employed to verify the deterministic computational models (M1 and M2) was probabilistic black-box because it does not place a lower bound on the required number of model simulations and therefore is suitable for computational models which are simulated only once. Conversely for the verification of the hybrid (M3) and stochastic (M4) computational models improved frequentist statistical hypothesis testing was employed setting the values of both input parameters *α* (i.e. probability of type I errors) and *β* (i.e. probability of type II errors) to 5%. Therefore the number of model simulations considered for the verification of computational models M3 and M4 was variable and computed relative to the values of the input parameters *α* and *β*, respectively fixed and was equal to one for computational models M1 and M2.

All approximate probabilistic model checking approaches supported by Mule (see Table 2) were previously introduced by other authors and are not directly dependent on PBLMSTL. Therefore a comparison between the different model checking approaches, although interesting, goes beyond the scope of this paper.

The computational models have been simulated, analysed and verified using the same regular desktop computer (Linux x64, Intel Core i5-2500 CPU @1.6 GHz, 16 GB DDR3 RAM memory). To assess the performance of the approach execution times have been recorded for all relevant steps of the model checking workflow.

Finally, for comparison purposes, the case studies and the corresponding computational models will not be described individually but in parallel considering the steps of the model checking workflow (i.e. model construction, multiscale spatio-temporal analysis, formal specification, model checking).

### Model construction

#### Rat cardiovascular system dynamics

The cardiovascular system comprises the heart, blood and blood vessels, and is the organ system responsible for delivering oxygen and nutrients to, and removing waste products from the entire organism. Its dynamics changes in case of a transient increase of the thoracic pressure (e.g. by performing the Valsalva manoeuvre) which leads to reduced blood flow in the right atrium, reduced cardiac output and decreased aortic pressure [13].

**Fig 8:**
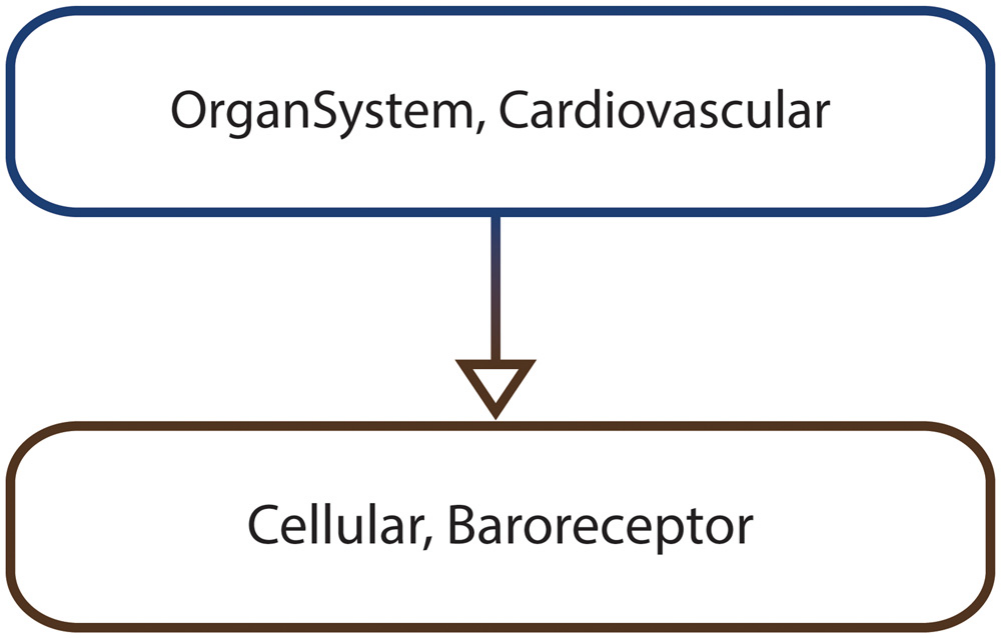
*MA* graph representing the multiscale organization of the rat cardiovascular system dynamics computational model.

In order to describe the behavioural changes of the cardiovascular system during the Valsalva manoeuvre Beard et al. built a multiscale non-spatial ODE model [13] by integrating two previously existing models. The first model is an abstract representation of the cardiovascular system [80]. Conversely the second model encodes the baroreflex mechanism [81] which is employed to maintain the blood pressure of an organism at approximately constant levels. One of the main advantages of the integrated multiscale model is that it enables relating changes at the entire cardiovascular system level with changes at the baroreflex mechanism level and vice versa, which was not possible when employing the constituent models separately. The hierarchical organization of the resulting model is encoded by the *MA* graph depicted in Fig 8.

For verification purposes the numeric state variables considered at the organ system scale are the thoracic pressure and the heart rate, and the aortic pressure at the cellular scale.

#### Uterine contractions of labour

Although it is known that usually during human labour regions across the entire uterus contract in a coordinated fashion the underlying mechanisms by which an initial local contraction propagates to the entire organ level are not fully understood [14].

One hypothesis is that a positive feedback loop is created between the tissue level contractions and the intrauterine pressure as follows: An initial tissue level contraction increases the intrauterine pressure and adds tension to the neighbouring regions, which in response start to contract, thus increasing the intrauterine pressure even further and adding tension to their corresponding neighbouring regions which also start to contract, and the entire process is repeated until all contractible regions across the entire organ are recruited.

**Fig 9:**
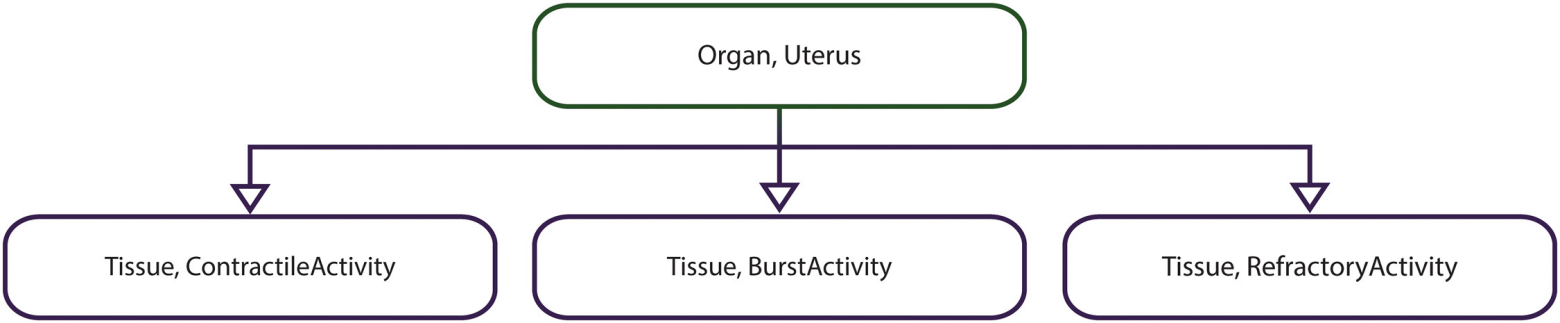
*MA* graph representing the multiscale organization of the uterine contractions of labour computational model.

In order to test this hypothesis Young and Barendse developed a corresponding predictive deterministic computational model [14]. The model was encoded as a cellular automaton in Mathematica and spans two levels of organization, the organ level for the uterus, and the tissue level for the uterine regions; see Fig 9 for the corresponding *MA* graph.

At the organ (i.e. uterus) scale the numeric state variable considered is the intrauterine pressure and space is encoded explicitly as a 4 × 4 grid, where each grid position represents a tissue (i.e. uterine region). Conversely at the tissue level there is no explicit representation of space and the recorded numeric state variables are the contractile, burst and refractory activities of the uterine regions.

#### *Xenopus laevis* cell cycle

The cell cycle is a fundamental biological process which is responsible for the replication/division of cells and is involved in the development and partial renewal of organisms. Its complexity is usually proportional to the complexity of the considered organism. Therefore it is studied in lower and less complex organisms such as the *Xenopus laevis* frog.

**Fig 10:**
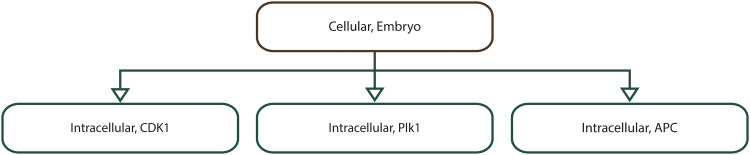
*MA* graph representing the multiscale organization of the *Xenopus laevis* cell cycle computational model.

To gain a better understanding of the *Xenopus laevis* embryonic cell cycle and how it affects cellular population growth the developers of the modelling software Morpheus [82] built a corresponding multiscale computational model [83]. The computational model describes how three proteins CDK1, Plk1 and APC regulate the cell cycle at the intracellular level using ODEs [15], and how cells divide and are displaced in 2D space at the cellular level using a CPM. The corresponding *MA* graph is depicted in Fig 10.

At the cellular level space is represented explicitly as a 52 × 52 grid recording the spatial distribution of the population of cells. Conversely at the intracellular level there is no explicit representation of space and the numeric state variables considered are the concentrations of CDK1, Plk1 and APC.

#### Acute inflammation of the gut and lung

There is no single definition of inflammation in the literature [84] but here we will interpret it as the response of a biological system to bodily damaging stimuli. Depending on the intensity of the stimulus an inflammatory response initiated in one organ can propagate to other organs and eventually lead to multiple organ failure [16].

**Fig 11:**
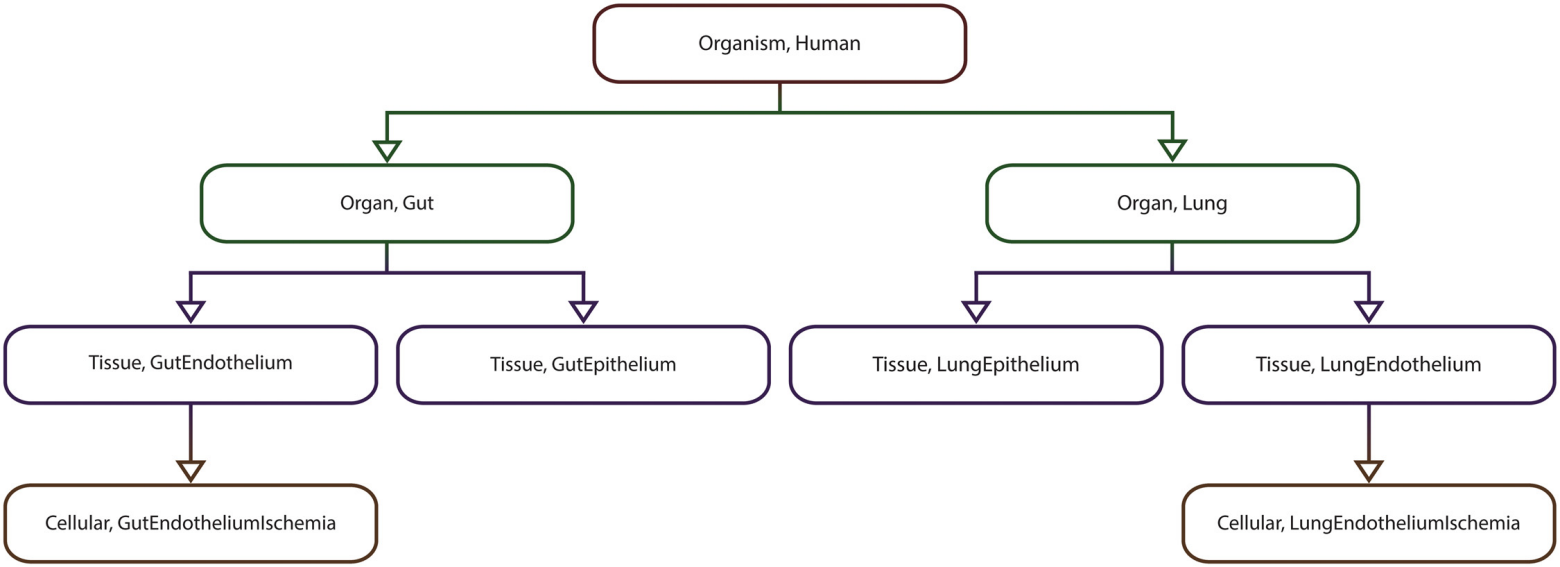
*MA* graph representing the multiscale organization of the acute inflammation of the gut and lung computational model.

To gain a better understanding of the relation between inflammatory responses and multiple organ failure, G. An [16] built a multiscale agent-based computational model using the software NetLogo which describes how the inflammation of either the gut (i.e. gut ischemia) or lung (i.e. pneumonia) could potentially lead to the failure of both organs. The levels of organization considered in the computational model are cellular (for representing endothelial and epithelial cells), tissue (for representing the organ luminal space, the blood vessel luminal space, and the endothelial and epithelial layers), and organ (for representing the gut and lung); see Fig 11 for the corresponding *MA* graph.

The organism level is not modelled explicitly and the corresponding vertex (Organism, Human) was added to the *MA* graph in Fig 11 only to ensure that its structure is tree-like. At the organ level space is not represented explicitly and the numeric state variables considered represent the amount of solute which leaked into the gut and lung. Conversely at the tissue level space is represented explicitly as a 31 × 31 grid where each grid position represents a cell. The tissue level numeric state variables considered for both gut and lung are the total concentration of cytoplasm and cell wall occludin, and the total cell damage by-product. At the cellular level the numeric state variables considered encode the level of ischemia for both gut and lung endothelial cells.

### Multiscale spatio-temporal analysis

The computational models M1–M4 were simulated and the simulation results were translated to MSTML.

The computational model simulation end time was computed as per Definition 1, S5 Text considering the PBLMSTL statements against which each computational model was verified.

The translation of the simulation results to MSTML comprises multiple steps. First of all the model simulation output is converted to csv format in order to ensure that the time series data provided as input to the multiscale spatio-temporal analysis module is represented in a uniform manner. Secondly an MSTML subfile is generated for each considered time point, numeric state variable and spatial region comprising one or multiple grid positions. In the end all subfiles are merged into a single MSTML file. The main difference between the csv and corresponding MSTML file is that for each time point the former records the values associated to entire discretised spatial domains, whereas the latter only captures the properties of the detected spatial entities. The main advantage of storing to disk the results of the csv to MSTML translation, and providing MSTML instead of csv files as input to the model checker is reusability. MSTML files can be employed for the evaluation of different PBLMSTL specifications in separate executions of the model checker without the need to run the csv to MSTML translation each time.

Execution times for the model simulation and subsequent translation steps corresponding to all computational models are given in Table 4.

**Table 4:** Model simulation and analysis execution times for the rat cardiovascular system dynamics, the uterine contractions of labour, the *Xenopus laevis* cell cycle, and the acute inflammation of the gut and lung case studies.

	Execution time (seconds)			
	M1	M2	M3	M4
**Model simulation**	37.22	1.13	1.79	329.6
**Convert simulation output to csv format**	0.33	0.02	1.31	2.62
**Generate MSTML subfiles**	25.52	25.15	12.06	64.82
**Merge subfiles into single MSTML file**	31.21	0.44	1.66	2.88

The steps considered are model simulation, conversion of the simulation output to csv format, generating an MSTML subfile for each considered time point, numeric state variable and spatial region comprising one or multiple grid positions, and merging subfiles into a single MSTML file. Depending on the computational model type (i.e. deterministic/stochastic/hybrid) and the formal specification against which it was verified, the number of considered model simulations, and time points per model simulation differed. Computational models are distinguished by their model id (i.e. M1–M4). The execution time of the deterministic computational models M1 and M2 was computed by simulating the models and analysing the resulting model simulation output one time. Conversely the execution time of the hybrid (M3) and stochastic (M4) computational models was computed as the average execution time of 1500, respectively 500 repeated runs of the model simulation and model simulation output analysis steps. The number of time points recorded for each model simulation was 30001 for computational model M1, 330 for M2, 103 for M3, and 1000 for M4. The number of time points was fixed due to two reasons. First of all the model simulation time interval considered was bounded. Secondly the model simulators recorded state changes considering a fixed user-defined simulation time step size (chosen by the original model authors).

The most time consuming step for the rat cardiovascular system dynamics (i.e. 37.22s) and the acute inflammation of the gut and lung (i.e. 329.6s) case studies was the model simulation due to the large number of time points considered (i.e. 30001), and the stochastic nature and high complexity associated with the model. Conversely the most time consuming step for the uterine contractions of labour (i.e. 25.15s) and *Xenopus laevis* cell cycle (i.e. 12.06s) case studies was generating the MSTML subfiles due to the spatial regions which have been automatically detected and analysed for each spatial state variable considered.

The least time consuming step for all case studies was converting the model simulation output to csv format.

### Formal specification

The generated MSTML files representing the behaviour of the computational models and the corresponding *MA* graphs are employed during the evaluation of the formal specifications described in natural language in Table 5. The equivalent PBLMSTL specifications for the rat cardiovascular system dynamics, the uterine contractions of labour, the *Xenopus laevis* cell cycle and the acute inflammation of the gut and lung case studies are given in S1, S2 and S3 Files, respectively S4 File.

**Table 5:** Natural language descriptions of the formal specifications employed for the rat cardiovascular system dynamics, the uterine contractions of labour, the *Xenopus laevis* cell cycle, and the acute inflammation of the gut and lung case studies.

MId	SId	Description
1	1	The probability is greater than 0.9 that after initiating the Valsava manoeuvre (time = 5000 ms) the thoracic pressure increases from the baseline value -4 to 16 for 10 seconds (time interval [5001 ms, 14999 ms]), and then drops back to the baseline value -4.
	2	The probability is greater than 0.9 that during the initial phase of the response (time interval [5001 ms, 6500 ms]) the aortic pressure increases and the heart rate decreases.
	3	The probability is less than 0.1 that after the initial response phase (time interval [5001 ms, 6500 ms]) the aortic pressure continues to increase or stay constant, respectively the heart rate continues to decrease or stay constant throughout the remainder of the Valsava interval (time interval [6501 ms, 14999 ms]).
2	4	The probability is greater than 0.9 that the intrauterine pressure increases/decreases with the contractile activity of uterine regions.
	5	The probability is less than 0.1 that the intrauterine pressure decreases when the entire uterus experiences an action potential burst.
	6	The probability is greater than 0.9 that the intrauterine pressure decreases when the entire uterus is in the refractory period.
3	7	The probability is greater than 0.9 that whenever the concentration of CDK1 reaches very high levels (in our case >96% of its maximum value) all cells will divide.
	8	The probability is greater than 0.9 that whenever the average concentration of APC increases and reaches its local maximum value no cell will divide.
	9	The probability is greater than 0.9 that the average concentrations of CDK1, Plk1 and APC increase and then decrease (i.e. oscillate) over time at least three times.
4	10	The probability is greater than 0.9 that if the level of cytoplasm occludin in the lung decreases then eventually the number of ischemic endothelial lung cells will increase.
	11	The probability is greater than 0.9 that always an increase of the cell damage by-product in the gut will lead to an increase of the cell damage by-product in the lung.
	12	The probability is greater than 0.9 that if the level of cell wall occludin in the gut decreases then eventually the amount of solute leaking in the gut lumen will increase.

Each model is identified by an id (column “MId”) and has an associated set of natural language statements. Conversely each natural language statement has a corresponding id (column “SId”) and description (column “Description”).

Throughout natural language specifications are translated to PBLMSTL such that the *i*-th natural language statement corresponds to the *i*-th PBLMSTL statement.

### Model checking

Each computational model has been verified against the relevant PBLMSTL statements 500 times, where each PBLMSTL statement was stored in a separate file. The main reason for repeating the model verification procedure 500 times for each computational model and PBLMSTL statement is to compute the variation of the model checker execution time between runs, and the variation of the number of MSTML files considered for the hybrid (M3) and stochastic (M4) computational models. Results obtained for each of the 500 model checker executions and PBLMSTL statements corresponding to the computational models M1, M2, M3 and M4 are given in S6, S7 and S8 Texts, respectively S9 Text. The output of the statistical analysis of the model checking results is summarized in Table 6.

**Table 6:** Statistical analysis of the model checking results for the rat cardiovascular system dynamics, the uterine contractions of labour, the *Xenopus laevis* cell cycle, and the acute inflammation of the gut and lung case studies.

MId	SId	% true PBLMSTL	#total MSTML		#true MSTML		#false MSTML		Execution time	
			*μ*	*σ*	*μ*	*σ*	*μ*	*σ*	*μ*	*σ*
1	1	100	1	0	1	0	0	0	17.67	0.12
	2	100	1	0	1	0	0	0	17.61	0.13
	3	100	1	0	0	0	1	0	17.8	0.36
2	4	100	1	0	1	0	0	0	0.55	0.01
	5	100	1	0	0	0	1	0	0.54	0.01
	6	100	1	0	1	0	0	0	0.54	0.01
3	7	100	28.79	2.04	28.61	1.62	0.19	0.44	35.35	2.44
	8	100	28	0	28	0	0	0	34.29	0.09
	9	100	28	0	28	0	0	0	35.36	0.99
4	10	100	28	0	28	0	0	0	87.39	0.72
	11	100	28	0	28	0	0	0	90.27	2.23
	12	100	28	0	28	0	0	0	87.03	0.65

Entries in the “MId” and “SId” columns represent the numeric identifiers associated with each computational model and its corresponding PBLMSTL statements. The “% true PBLMSTL” column describes what percentage of the 500 model checker executions concluded that the PBLMSTL statement is true. “#total MSTML” represents the total number of MSTML files evaluated for the PBLMSTL statement during a single model checker execution; columns “#true MSTML” and “#false MSTML” represent the number of MSTML files for which the PBLMSTL statement was evaluated true, respectively false, during a single model checker execution. “Execution time” records the average runtime in seconds for each model checker execution. “*μ*” and “*σ*” represent the mean and standard deviation. Due to the deterministic nature of computational models M1 and M2 only one simulation trace was employed for their verification (see table rows corresponding to MId 1 and MId 2, table column 4). Conversely the number of simulation traces considered for the verification of computational models M3 and M4 was equal to ≈28 (see table rows corresponding to MId 3 and MId 4, table column 4), and was computed as a function of the input parameters *α* and *β* of the improved statistical hypothesis testing model checking approach. The model simulation traces employed for the verification of computational models M3 and M4 were chosen randomly from the collection of 1500, respectively 500 simulation traces generated to compute the average execution times given in Table 4.

Empirical evidence shows that all computational models are correct relative to the formal specifications derived from the original papers introducing the models.

Due to the deterministic nature of computational models M1 and M2, the corresponding model checking results were obtained by considering a single MSTML file, and therefore were identical across all 500 model checker executions. The main difference between the PBLMSTL statements considered is that in case of statements 1, 2, 4 and 6 the estimated probability *p* for them to hold, computed as #true MSTML divided by #total MSTML, was *p* = (1 / 1) = 1, whereas for the PBLMSTL statements 3 and 5 it was *p* = (0 / 1) = 0. However since the associated probabilistic specification for the PBLMSTL statements 1, 2, 4 and 6 was *p* > 0.9 (i.e. 1 > 0.9), and *p* < 0.1 (i.e. 0 < 0.1) for the PBLMSTL statements 3 and 5, all PBLMSTL statements hold.

Conversely in case of the hybrid (M3) and stochastic (M4) computational models the model checking results were obtained by considering multiple MSTML files. Moreover the number of MSTML files against which the corresponding PBLMSTL statements evaluated true varied between model checker executions (e.g. see Table 6, row corresponding to SId 7). However the result of the model verification procedure was always the same (see Table 6, column 3).

The average model checker execution times corresponding to the verification of the deterministic computational models M1 and M2 were smaller than for the hybrid, respectively stochastic computational models M3 and M4. This is due to the difference in the number of MSTML files considered which was one for computational models M1 and M2, and ≊28 for computational models M3 and M4. Moreover the variation in the average model checker execution times between the computational models M1 and M2, respectively M3 and M4 is due to the difference in the number of time points considered per model simulation which was 30001 for M1 and 330 for M2, respectively 103 for M3 and 1000 for M4. Average model checker execution times corresponding to the same computational model but different PBLMSTL statements were approximately equal throughout because most of the execution time is spent on reading the MSTML file(s) from disk and not the evaluation of the PBLMSTL statements.

By storing the PBLMSTL statements corresponding to a computational model in separate files each MSTML file read by the model checker from disk is evaluated against only one rather than all PBLMSTL statements. Therefore in order to reduce the average model checker execution time all PBLMSTL statements corresponding to the same computational model could be written into a single file. A comparison between average execution times obtained for 500 model checker executions considering all PBLMSTL statements written into single, respectively multiple separate files are given in Table 7. Regardless of the computational model considered the average model checker execution time was approximately three times smaller when storing PBLMSTL statements in single rather than multiple separate files. The main reason for this is that the total number of MSTML files read from disk, which takes up most of the model checker execution time, was reduced by a factor equal to the number of PBLMSTL statements considered (i.e. 3).

**Table 7:** Comparison of average model checker execution times when PBLMSTL statements corresponding to a computational model are stored in a single, respectively multiple separate files.

MId	Execution time (seconds)	
	Single file	Separate files
1	17.9	53.07
2	0.56	1.63
3	36.3	105
4	87.51	264.68

The “MId” column records the numeric identifiers associated with each computational model. Average model checker execution times corresponding to PBLMSTL statements stored in a single, respectively multiple separate files are given in columns “Single file” and “Separate files”.

The model checker execution times given in Tables 6 and 7 were measured when providing pre-generated MSTML files as input to Mule. However Mule can be additionally employed to verify computational models by generating MSTML files on demand. In order to measure the model checker execution time when all MSTML files are generated on-demand the computational model M3 was verified 500 times relative to the corresponding PBLMSTL statements stored in a single file, without providing any pre-generated MSTML files as input. The average execution time of the 500 runs was 317.7s i.e. ≈9 times more than when providing pre-generated MSTML files as input (i.e. 36.3s). The large difference in execution time is due to the fact that when generating MSTML files on-demand Mule needs to wait for the MSTML files to be generated (i.e. for the computational model to be simulated and the model simulation output to be translated to MSTML) before evaluating the PBLMSTL specification against them. Therefore there is a model checker execution time overhead when verifying computational models using on-demand generated MSTML files. The magnitude of the execution time overhead depends on the number of MSTML files against which the PBLMSTL specification is evaluated, and the time required to generate a new model simulation and translate the model simulation output to MSTML.

**Fig 12:**
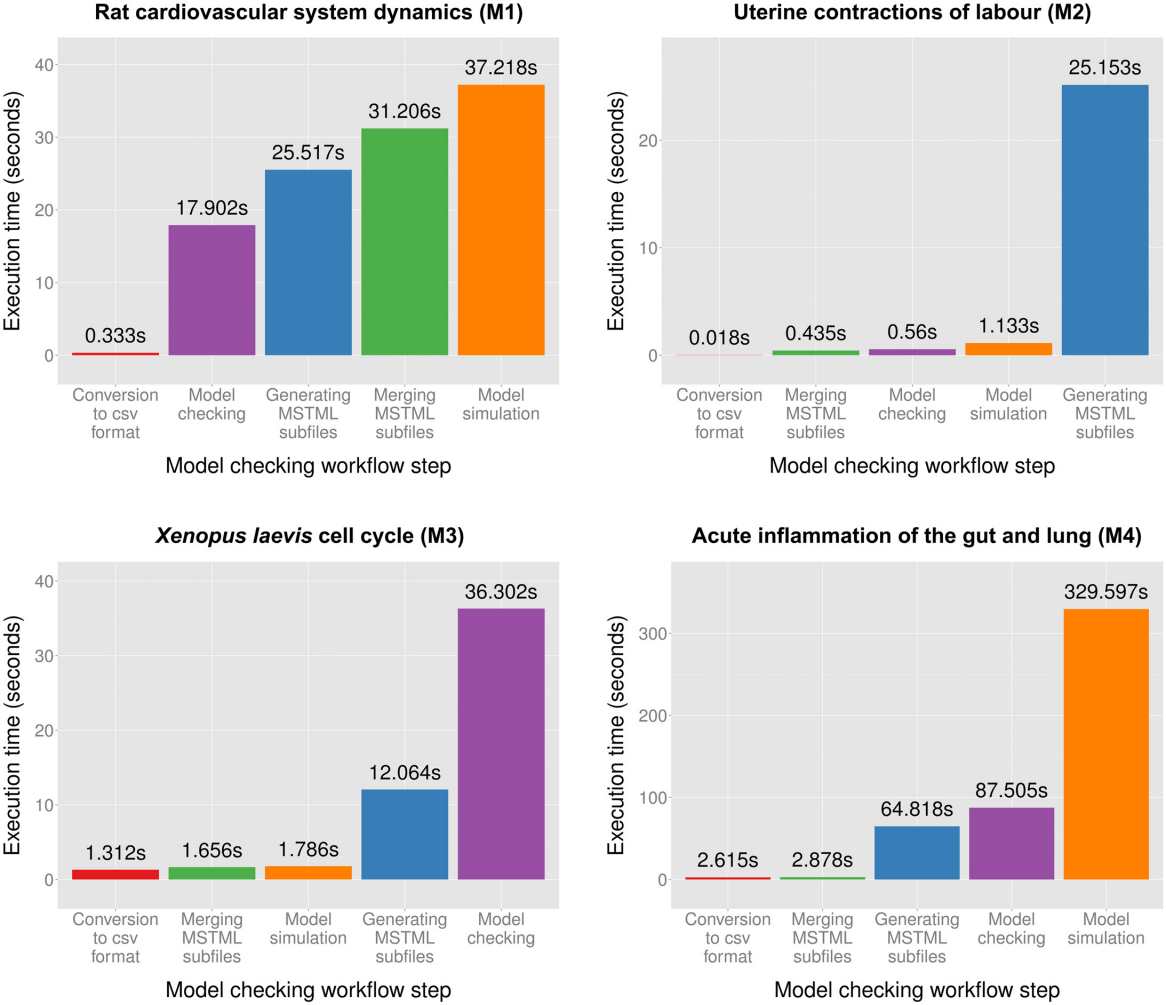
Average execution times (measured in seconds) corresponding to the verification of the rat cardiovascular system dynamics, the uterine contractions of labour, the *Xenopus laevis* cell cycle, and the acute inflammation of the gut and lung computational models. Execution times were recorded for the computational model simulation, converting the output to csv format, generating MSTML subfiles for each considered time point, numeric state variable and spatial entity, merging the subfiles into a single MSTML file, and model checking.

A comparison between the average execution times recorded for simulating the model, translating the output to MSTML and verifying it using model checking is given in Fig 12.

The most time consuming step in the model checking workflow for both the cardiovascular system dynamics and acute inflammation of the gut and lung case studies is the model simulation. This is due to the large number of time points considered in case of the former, and the high complexity associated with the stochastic computational model in case of the latter. Conversely for the uterine contractions of labour case study the most time consuming step in the model checking workflow is generating the MSTML subfiles due to the additional need to automatically detect and analyse spatial regions of three types (i.e. corresponding to the contractile, burst and refractory activities) for each simulation time point. In contrast, the most time consuming step in the model checking workflow for the *Xenopus laevis* cell cycle case study is model checking due to the need to evaluate each PBLMSTL statement against multiple MSTML files. The least time consuming step in the model checking workflow for all case studies is converting the simulation output to csv format.

For reproducibility purposes the *MA* graph, the pre-generated MSTML file(s), the formal PBLMSTL specification, and the excerpts from the referenced papers used to write the formal specification for each case study are made available as supplementary materials; see Table 8 for details. Due to file size constraints only a subset of the total number of generated MSTML files was made available for the *Xenopus laevis* cell cycle (see S3 Dataset) and the acute inflammation of the gut and lung (see S4 Dataset) case studies; the complete datasets are made freely available online at http://mule.modelchecking.org/case-studies.

**Table 8:** Availability of the *MA* graph, the generated MSTML file(s), the formal PBLMSTL specification, and the excerpts from the referenced papers used to write the formal specification for each case study.

MId	*MA* graph	MSTML file(s)	PBLMSTL specification	Excerpts from referenced papers
1	S5 File	S1 Dataset	S1 File	S10 Text
2	S6 File	S2 Dataset	S2 File	S11 Text
3	S7 File	S3 Dataset	S3 File	S12 Text
4	S8 File	S4 Dataset	S4 File	S13 Text

The “MId” column records the numeric identifiers associated with each computational model.

## Discussion

The need for reasoning about how systems evolve over multiple temporal and spatial scales has been previously emphasized in the literature. For instance Van de Weghe et al. [85] have defined a theoretical framework which enables describing and analysing how geographical phenomena observed at higher scales are reflected at lower scales and vice versa. However there is a lack of corresponding model checking approaches for computational models of such systems.

To the best of our knowledge the only related multiscale model checking approach which explicitly distinguishes between multiple spatial scales without (initially) accounting for time was introduced by Grosu et al. [86] for detecting patterns in images. The multiscale representation of space was created by recursively splitting a spatial domain in quadrants (a finite number of times) and representing the resulting hierarchy as a quadtree. A formal logic called Linear Spatial Superposition Logic (LSSL) and a corresponding model checking algorithm were introduced in order to encode specifications relative to spatial subdomains along a linear path through the quadtree. More recently both the formal logic and corresponding model checking algorithm were extended by Gol et al. [87] to account for branching paths through quadtrees (Tree Spatial Superposition Logic), and by Haghighi et al. [88] to account for the evolution of the quadtrees over time (SpaTel). Although efficient for pattern detection (and generation) these approaches could be potentially too restrictive for reasoning about general multiscale systems since only one spatial domain is considered and the relationship between consecutive levels/scales is fixed. Moreover it is not possible to describe how spatial entities potentially spanning multiple quadrants of the spatial domain, and their properties change over time.

In this paper we have introduced a novel multiscale spatio-temporal meta model checking methodology which enables automatically verifying multilevel computational models of biological systems relative to specifications describing the desired/expected system behaviour.

Our approach is generic and supports multilevel computational models of biological systems encoded using various high-level modelling formalisms (e.g. CPMs, ABMs) because it is defined relative to time series data and not the models used to produce them. This is illustrated by the four case studies which were formally encoded using ODEs (rat cardiovascular system dynamics), CAs (uterine contractions of labour), CPMs (*Xenopus laevis* cell cycle), ABMs (acute inflammation of the gut and lung) or combinations thereof.

Although the model checker is flexible regarding the modelling formalism employed to encode the computational models it requires that the model simulation output is translated to the standard MSTML format. During the translation process non-spatial state variables (e.g. concentrations) are mapped directly from their native format to MSTML. Conversely in case of spatial state variables the multiscale spatio-temporal analysis module is additionally executed for automatically detecting emergent spatial entities (e.g. clusters) and computing their properties (e.g. area).

The model checker can be adapted automatically to case study specific spatial entity types (e.g. 3D spatial structure) and/or properties (e.g. minimum distance to a fixed point) not covered by our multiscale spatio-temporal analysis module. External analysis tools can be employed to automatically detect and analyse these case study specific spatial entities, and to convert the output to the MSTML format. The corresponding instance of the multiscale spatio-temporal meta model checker can be generated automatically based on a configuration file without the need to modify the implementation by hand.

The set of MSTML files representing the model behaviour can be generated either before or during the evaluation of a PBLMSTL specification. In case of the latter the model checker must be executed with an additional parameter representing the path to an external program which runs model simulations on demand, translates the output to MSTML and stores the resulting files in a predefined location. The overhead of generating MSTML files during (i.e. on demand) rather than before the evaluation of the PBLMSTL specification depends on the number of required MSTML files and the time required to simulate the computational model and translate the output to MSTML.

We have illustrated the applicability and flexibility of the model checker Mule by verifying four systems biology computational models previously published in the literature relative to formal specifications derived from the original papers introducing the models. Although only the probabilistic black box (see rat cardiovascular system dynamics and uterine contractions of labour case studies) and frequentist statistical model checking algorithms (see *Xenopus laevis* cell cycle and acute inflammation of gut and lung case studies) were employed here, additional frequentist (i.e. based on Chernoff-Hoeffding bounds) and Bayesian (i.e. hypothesis testing, mean and variance estimate based) model checking algorithms are supported.

The scalability of the entire model verification workflow depends on the scalability of the model simulation, multiscale spatio-temporal analysis and model checking steps. The execution time of the model simulation depends on the complexity of the system under consideration. Conversely the execution times of both the multiscale spatio-temporal analysis and the model checker depend on the size of the simulation output. In addition, the model checker execution time also depends on the formal specification. Our expectation is that scaling up to more complex systems will lead to an increase of the computational model complexity but not necessarily the size of the simulation output and/or formal specification. Therefore the expected scalability bottleneck of the entire model checking workflow is the model simulation and not the model verification step. This is supported by empirical evidence obtained from the case studies; the ratio between the maximum and minimum execution times for the model simulation step was ≊290, ≊5 for the multiscale spatio-temporal analysis, and ≊156 for model checking. In addition it would be possible to speed up the model checking step by evaluating MSTML files against the formal specification in parallel rather than sequentially as it is done now.

To enable computational modellers to easily adopt our approach for the verification of multilevel computational models of biological systems the model checker Mule (source code, binary, Docker image) and relevant supplementary materials are made freely available online via the official web page http://mule.modelchecking.org.

Building on our model checking methodology we could consider the following extensions in the future. First of all it is assumed throughout that computational models are translatable to an MSSpDES representation which means that any computational model encoded using a potentially incompatible high-level modelling formalism will be translated to a corresponding MSSpDES representation subject to potential approximation errors (e.g. consider continuous computational models). Alternative representations could be employed instead. Secondly, although our methodology is automatically reconfigurable according to case study specific spatial entity types and measures, there is a need for the corresponding spatio-temporal analysis tools to be developed. The spatio-temporal analysis modules described here are currently restricted to pseudo-3D spatial entity types and measures, but could be extended in the future for other numbers of dimensions. Thirdly the efficiency of Mule could be improved by supporting on-the-fly model checking. However this means that all computational models considered would need to be explicitly translated to a common (e.g. MSSpDES) representation before being verified. Fourthly the efficacy of the methodology was tested only against *in silico* generated time series data, but our expectation is that it could be employed for analysing experimental time series data as well. Moreover since the methodology is not restricted to biological case studies, non-biological case studies could be additionally considered in order to test the limitations of the approach and potentially identify new features which could be included in forthcoming versions. Finally the efficacy of the multiscale model checking approach could be assessed in the future in the context of robustness analysis, parameter estimation/synthesis, and model construction problems.

## Conclusions

In this paper we have defined a multiscale spatio-temporal meta model checking methodology which enables the automatic verification of multilevel computational models with respect to how both numeric (e.g. concentrations) and spatial (e.g. area) properties change over time considering multiple levels of organization.

The approach was implemented in our model checking software Mule which is made freely available online. To encourage potential contributions (e.g. extensions) the source code is hosted in a public GitHub repository. For flexibility purposes Mule supports both frequentist and Bayesian, estimate and statistical hypothesis testing based model checking approaches.

We have illustrated the applicability of the model verification approach using four representative systems biology case studies published in the literature, namely the rat cardiovascular system dynamics, the uterine contractions of labour, the *Xenopus laevis* cell cycle and the acute inflammation of the gut and lung.

Our approach enables computational modellers to construct reliable multilevel computational models of biological systems in a faster manner than it is done currently. These computational models could then be potentially translated into systems medicine to provide patient specific predictions on the evolution of diseases and their treatment across multiple levels of organization.

## Supporting Information

S1 Text Brief description of the *in silico* computational model verification approach called model checking. (PDF)

S2 Text Description of how to construct the *MA* graph corresponding to a given biological system. (PDF)

S3 Text Description of the Multiscale Spatial Temporal Markup Language. (PDF)

S4 Text Formal definition of BLMSTL syntax and semantics. (PDF)

S5 Text Proof that the multiscale spatio-temporal model checking problem is well-defined. (PDF)

S6 Text Model checking results for the rat cardiovascular system dynamics case study. (PDF)

S7 Text Model checking results for the uterine contractions of labour case study. (PDF)

S8 Text Model checking results for the *Xenopus laevis* cell cycle case study. (PDF)

S9 Text Model checking results for the acute inflammation of the gut and lung case study. (PDF)

S10 Text Excerpts from the literature employed to write the formal specification for the rat cardiovascular system dynamics case study. (PDF)

S11 Text Excerpts from the literature employed to write the formal specification for the uterine contractions of labour case study. (PDF)

S12 Text Excerpts from the literature employed to write the formal specification for the *Xenopus laevis* cell cycle case study. (PDF)

S13 Text Excerpts from the literature employed to write the formal specification for the acute inflammation of the gut and lung case study. (PDF)

S1 File Formal PBLMSTL specification for the rat cardiovascular system dynamics case study. (IN)

S2 File Formal PBLMSTL specification for the uterine contractions of labour case study. (IN)

S3 File Formal PBLMSTL specification for the *Xenopus laevis* cell cycle case study. (IN)

S4 File Formal PBLMSTL specification for the acute inflammation of the gut and lung case study. (IN)

S5 File Multiscale architecture graph for the rat cardiovascular system dynamics case study. (XML)

S6 File Multiscale architecture graph for the uterine contractions of labour case study. (XML)

S7 File Multiscale architecture graph for the *Xenopus laevis* cell cycle case study. (XML)

S8 File Multiscale architecture graph for the acute inflammation of the gut and lung case study. (XML)

S1 Dataset Dataset of MSTML files generated for the rat cardiovascular system dynamics case study. (ZIP)

S2 Dataset Dataset of MSTML files generated for the uterine contractions of labour case study. (ZIP)

S3 Dataset Dataset of MSTML files generated for the *Xenopus laevis* cell cycle case study. (ZIP)

S4 Dataset Dataset of MSTML files generated for the acute inflammation of the gut and lung case study. (ZIP)
