# Supplementary material for: A Novel Method to Verify Multilevel Computational Models of Biological Systems Using Multiscale Spatio-Temporal Meta Model Checking
Source: PLoS One. 2016 May 17;11(5):e0154847. doi: 10.1371/journal.pone.0154847 (PMC4871515; doi:10.1371/journal.pone.0154847)
Supplement: S1 Text — (PDF) [file pone.0154847.s001.pdf]

Formal verification using model checking

Model checking [1, 8] is a formal method for verifying if an abstract representation of a system (i.e. a model) is correct relative to a formal specification describing the desired/expected system behaviour. The general model checking workflow comprises the following steps:

1. **Model construction**: Creating an abstract representation of the system (e.g. a computational model);
2. **Formal specification**: Encoding the formal specification describing the desired/expected system behaviour;
3. **Model checking**: Automatically verifying the correctness of the model relative to the formal specification.

Model construction

Computational models of biological systems are usually encoded using high level modelling formalisms such as (ordinary) differential equations [3, 5], Petri nets [2, 11, 14], process algebras (e.g. Bio-PEPA [7, 13]), software-specific (e.g. BIOCHAM [6], BioNetGen [9, 12]) rule-based modelling languages, or timed automata [4, 18]. However for model checking purposes these computational models are usually translated to a single common low level (probabilistic) labelled state transition (LSTS) [1, Chapters 2 and 10] representation. The main reason for this is that the model checking algorithms can then be defined only once relative to a single rather than multiple modelling formalisms.

Executions or simulations of LSTSs are represented as discrete sequences of states where transitions between states are triggered by events and are executed instantaneously. Relevant system properties are encoded by state variables whose values may change between states. Therefore the behaviour of the system is usually defined by time series data describing how the values of the state variables change over time.

Formal specification

The specifications against which the models are verified comprise statements which describe what is the expected system behaviour, respectively how the state variable values are expected to change over time. One class of formal languages which enable reasoning about system changes over time, and which we consider here, are called temporal logics. Depending on the underlying structure of time the temporal logic can be either linear or branching; here only linear temporal logics are considered.

One of the most commonly employed temporal logics which assumes a linear representation of time is called Linear Temporal Logic (LTL) [10, 19]. Statements written in this language can be decomposed into three types of propositions, namely atomic, Boolean and temporal.

Atomic propositions are Boolean expressions defined over (state) variables (e.g. concentration of a protein $[X]$), constants (e.g. real number 2.3) and predicate symbols (e.g. comparison predicate $>$), which cannot be divided into simpler logic statements.

Conversely a Boolean proposition is a compound statement comprising a Boolean operator and logic propositions (denoted here by ϕ):

- $\sim \phi$ (not): The **negation** of logic proposition ϕ is true i.e. ϕ is false;
- $\phi_1 \wedge \phi_2$ (and): Logic proposition ϕ_1 is true **and** logic proposition ϕ_2 is true;
- $\phi_1 \vee \phi_2$ (or): Logic proposition ϕ_1 is true **or** logic proposition ϕ_2 is true;
- $\phi_1 \Rightarrow \phi_2$ (implication): Logic proposition ϕ_1 is true **implies** logic proposition ϕ_2 is true;
- $\phi_1 \Leftrightarrow \phi_2$ (equivalence): Logic proposition ϕ_1 is true **equivalent to** logic proposition ϕ_2 is true,

where $\sim$ is a unary Boolean operator, respectively $\wedge$, $\vee$, $\Rightarrow$, $\Leftrightarrow$ are binary Boolean operators.

Finally temporal propositions are used to reason about how a system changes over time. They comprise a temporal operator and logic propositions (similarly denoted by ϕ):

- $F\phi$ (**F**uture): **Eventually** logic proposition ϕ holds;
- $G\phi$ (**G**lobally): Logic proposition ϕ holds **always**;
- $\phi_1 U \phi_2$ (**U**ntil): Logic proposition ϕ_1 holds **until** logic proposition ϕ_2 holds;
- $X\phi$ (ne**X**t): Logic proposition ϕ holds in the **next** time point,

where F, G, X are unary temporal operators, and U is a binary temporal operator.

One of the limitations of LTL is that it does not enable writing logic properties relative to finite sequences of states (e.g. the first 10 states) in a given computation path. Therefore, in case of complex systems whose behaviour yields an infinite sequence of states the evaluation of LTL logic properties could be potentially intractable. In such cases logic properties considering finite subsequences of states, called bounded logic properties, can be employed instead.

To enable writing such bounded logic properties, various extensions of LTL were developed. One of these extensions is called Bounded Linear Temporal Logic (BLTL). As indicated by Jha et al. [17] BLTL augments classic LTL temporal operators F, G and U with an upper bound $t \in \mathbb{Q}_{\geq 0}$:

- $F^t\ \phi$: Eventually logic proposition ϕ holds within the time interval $[0, t]$;
- $G^t\ \phi$: Logic proposition ϕ holds always within the time interval $[0, t]$;
- $\phi_1\ U^t\ \phi_2$: Logic proposition ϕ_1 holds until logic proposition ϕ_2 holds within the time interval $[0, t]$.

Moreover as suggested later by Jha and Ramanathan [16] it is possible to additionally augment the temporal operators F, G and U with intervals $[t_1, t_2]$, $t_1, t_2 \in \mathbb{Q}_{\geq 0}$, such that logic propositions are evaluated against bounded time intervals which start at time point $t_1 \neq 0$.

Model checking

Model checking algorithms take a model and a formal specification as input and decide if the model behaviour conforms to the given specification, respectively if the values of the state variables change over time as expected. Such algorithms are usually implemented in software tools called model checkers which automate the entire verification process.

Depending if the model under consideration and corresponding formal specification are probabilistic or not, the employed model checking algorithms can be either exhaustive/approximate probabilistic or exhaustive non-probabilistic.

Exhaustive (non-)probabilistic model checking algorithms explore the entire system state space in order to decide if the model is correct relative to the given specification. The main advantage of such approaches is that they guarantee the (in)correctness of the model because all possible system states are potentially considered. Conversely one of the main disadvantages is that the computational complexity of exhaustive algorithms is proportional to the number of states considered. Therefore they can only be employed for models whose state space can be explored in reasonable time.

In contrast approximate probabilistic model checking algorithms explore the system state space only partially by considering a finite number of finite model simulations. One of the main disadvantages of such approaches is that the result of the model checking procedure is only an approximation and is not guaranteed to be correct. However several approximate probabilistic algorithms (e.g. [15, 20, 21]) enable the user to place an upper bound on the approximation error and thus ensure that the model checking result is provided with a certain degree of confidence (e.g. 95%). Conversely one of the main advantages of such approaches is that their complexity does not depend on the number of possible system states and therefore can be employed for systems with both small and large, potentially infinite, state spaces.

References

[1] Christel Baier and Joost-Pieter Katoen. *Principles of Model Checking.* The MIT Press, June 2008.

[2] Paolo Ballarini, Emmanuelle Gallet, Pascale Le Gall, and Matthieu Manceny. Formal Analysis of the Wnt/β-catenin through Statistical Model Checking. In Tiziana Margaria and Bernhard Steffen, editors, *Leveraging Applications of Formal Methods, Verification and Validation. Specialized Techniques and Applications*, number 8803 in Lecture Notes in Computer Science, pages 193–207. Springer Berlin Heidelberg, October 2014.

[3] Jiří Barnat, Luboš Brim, Ivana Černá, Sven Dražan, Jana Fabriková, Jan Láník, David Šafránek, and Hongwu Ma. : A Framework for Parallel Analysis of Biological Models. *Electronic Proceedings in Theoretical Computer Science*, 6:31–45, October 2009. arXiv: 0910.0928.

[4] Ezio Bartocci, Flavio Corradini, Emanuela Merelli, and Luca Tesei. Detecting synchronisation of biological oscillators by model checking. *Theoretical Computer Science*, 411(20):1999–2018, April 2010.

[5] Grégory Batt, Calin Belta, and Ron Weiss. Model Checking Genetic Regulatory Networks with Parameter Uncertainty. In Alberto Bemporad, Antonio Bicchi, and Giorgio Buttazzo, editors, *Hybrid Systems: Computation and Control*, number 4416 in Lecture Notes in Computer Science, pages 61–75. Springer Berlin Heidelberg, January 2007.

[6] Laurence Calzone, Nathalie Chabrier-Rivier, François Fages, and Sylvain Soliman. Machine Learning Biochemical Networks from Temporal Logic Properties. In Corrado Priami and Gordon Plotkin, editors, *Transactions on Computational Systems Biology VI*, number 4220 in Lecture Notes in Computer Science, pages 68–94. Springer Berlin Heidelberg, 2006.

[7] Federica Ciocchetta, Stephen Gilmore, Maria Luisa Guerriero, and Jane Hillston. Integrated Simulation and Model-Checking for the Analysis of Biochemical Systems. *Electronic Notes in Theoretical Computer Science*, 232:17–38, March 2009.

[8] E. M. Clarke, Orna Grumberg, and Doron Peled. *Model Checking*. MIT Press, 1999.

[9] Edmund M. Clarke, James R. Faeder, Christopher J. Langmead, Leonard A. Harris, Sumit Kumar Jha, and Axel Legay. Statistical Model Checking in BioLab: Applications to the Automated Analysis of T-Cell Receptor Signaling Pathway. In Monika Heiner and Adelinde M. Uhrmacher, editors, *Computational Methods in Systems Biology*, number 5307 in Lecture Notes in Computer Science, pages 231–250. Springer Berlin Heidelberg, 2008.

[10] Bernd Finkbeiner and Henny Sipma. Checking finite traces using alternating automata. *Electronic Notes in Theoretical Computer Science*, 55(2):147–163, October 2001.

[11] David Gilbert, Monika Heiner, and Sebastian Lehrack. A Unifying Framework for Modelling and Analysing Biochemical Pathways Using Petri Nets. In Muffy Calder and Stephen Gilmore, editors, *Computational Methods in Systems Biology*, number 4695 in Lecture Notes in Computer Science, pages 200–216. Springer Berlin Heidelberg, 2007.

[12] Haijun Gong, Paolo Zuliani, Anvesh Komuravelli, James R. Faeder, and Edmund M. Clarke. Computational Modeling and Verification of Signaling Pathways in Cancer. In Katsuhisa Horimoto, Masahiko Nakatsui, and Nikolaj Popov, editors, *Algebraic and Numeric Biology*, number 6479 in Lecture Notes in Computer Science, pages 117–135. Springer Berlin Heidelberg, 2012.

[13] Maria Luisa Guerriero. Qualitative and Quantitative Analysis of a Bio-PEPA Model of the Gp130/JAK/STAT Signalling Pathway. In Corrado Priami, Ralph-Johan Back, and Ion Petre, editors, *Transactions on Computational Systems Biology XI*, number 5750 in Lecture Notes in Computer Science, pages 90–115. Springer Berlin Heidelberg, 2009.

[14] Monika Heiner, David Gilbert, and Robin Donaldson. Petri Nets for Systems and Synthetic Biology. In Marco Bernardo, Pierpaolo Degano, and

Gianluigi Zavattaro, editors, *Formal Methods for Computational Systems Biology*, number 5016 in Lecture Notes in Computer Science, pages 215–264. Springer Berlin Heidelberg, 2008.

[15] Thomas Hérault, Richard Lassaigne, Frédéric Magniette, and Sylvain Peyronnet. Approximate probabilistic model checking. In Bernhard Steffen and Giorgio Levi, editors, *Verification, Model Checking, and Abstract Interpretation*, number 2937 in Lecture Notes in Computer Science, pages 73–84. Springer Berlin Heidelberg, January 2004.

[16] S.K. Jha and A. Ramanathan. Quantifying uncertainty in epidemiological models. In *BioMedical Computing (BioMedCom), 2012 ASE/IEEE International Conference on*, pages 80–85, Washington, DC, Dec 2012. IEEE.

[17] Sumit K. Jha, Edmund M. Clarke, Christopher J. Langmead, Axel Legay, André Platzer, and Paolo Zuliani. A bayesian approach to model checking biological systems. In Pierpaolo Degano and Roberto Gorrieri, editors, *Computational Methods in Systems Biology*, number 5688 in Lecture Notes in Computer Science, pages 218–234. Springer Berlin Heidelberg, January 2009.

[18] Bing Liu, Soonho Kong, Sicun Gao, Paolo Zuliani, and Edmund M. Clarke. Parameter Synthesis for Cardiac Cell Hybrid Models Using δ-Decisions. In Pedro Mendes, Joseph O. Dada, and Kieran Smallbone, editors, *Computational Methods in Systems Biology*, number 8859 in Lecture Notes in Computer Science, pages 99–113. Springer International Publishing, November 2014.

[19] Amir Pnueli. The temporal logic of programs. In *Foundations of Computer Science, 1977., 18th Annual Symposium on*, pages 46–57, Providence, RI, USA, Oct 1977. IEEE.

[20] Håkan L. S. Younes. *Verification and Planning for Stochastic Processes with Asynchronous Events.* Doctor of philosophy, Carnegie Mellon, Pittsburgh, 2005.

[21] Håkan L. S. Younes, Marta Kwiatkowska, Gethin Norman, and David Parker. Numerical vs. statistical probabilistic model checking. *International Journal on Software Tools for Technology Transfer*, 8(3):216–228, June 2006.
