# Supplementary material for: A Novel Method to Verify Multilevel Computational Models of Biological Systems Using Multiscale Spatio-Temporal Meta Model Checking
Source: PLoS One. 2016 May 17;11(5):e0154847. doi: 10.1371/journal.pone.0154847 (PMC4871515; doi:10.1371/journal.pone.0154847)
Supplement: S2 Text — (PDF) [file pone.0154847.s002.pdf]

# Description of how to construct the $MA$ graph corresponding to a biological system

Given a biological system $BS$ the corresponding $MA$ graph is constructed as follows; see Fig. 1 for an illustrative example. First of all both $BS$ and its corresponding subsystems relevant to the addressed biological question, together with their associated scale, are encoded as a set of vertices $V_{MA}$ (see Fig. 1.1). Secondly starting from the vertices at the most coarse-grained scales directed edges are added towards their constituent subsystems. This step is repeated for all considered finer-grained scales until the entire hierarchical structure of $BS$ is explicitly represented. Depending on the hierarchical representation of $BS$, vertices at the same depth in $MA$ could correspond to different scales (see Fig. 1.2). The resulting $MA$ graph should be a directed connected rooted acyclic graph, which means there exists a unique path from the root to every vertex, respectively $MA$ should not contain any cycles.

However for some systems it is possible that the resulting $MA$ graph contains vertices that have multiple incoming directed edges, which means they are part of more than one biological subsystem. For instance in Fig. 1.2 the vertex (Tissue, CardiacMuscle) has two parent vertices, namely (OrganSystem, Cardiovascular) and (OrganSystem, Musculoskeletal). The main disadvantage of allowing multiple incoming directed edges for the same vertex is that in order to differentiate between the context in which the biological subsystem is described, the corresponding parent vertex needs to be explicitly mentioned. Moreover multiple incoming directed edges are not allowed in rooted directed trees because the path to the corresponding vertex from the root is no longer unique. To overcome this issue we assume that every vertex with $n$ incoming directed edges is replicated $n-1$ times such that each instance of the vertex has only one incoming directed edge. In order to distinguish between the $n$ vertex instances either the associated scale or subsystem label needs to be renamed accordingly (see Fig. 1.3).

Finally it may happen that in the updated $MA$ graph multiple vertices $v_1, v_2, ..., v_p$ do not have any incoming directed edge and should therefore be labelled as root vertices (see Fig. 1.3). However in a rooted directed tree there can be only one root vertex. To address this issue an artificial root vertex $v_{root}$ can be created and added to $V_{MA}$, whose corresponding scale is higher than that of $v_1, v_2, ..., v_p$, and which will be connected via a directed outgoing edge with each vertex $v_i$, $i = \overline{1,p}$. Although the vertex $v_{root}$ will not be explicitly considered in the model it ensures that the structure of $MA$ is tree-like (see Fig. 1.4).

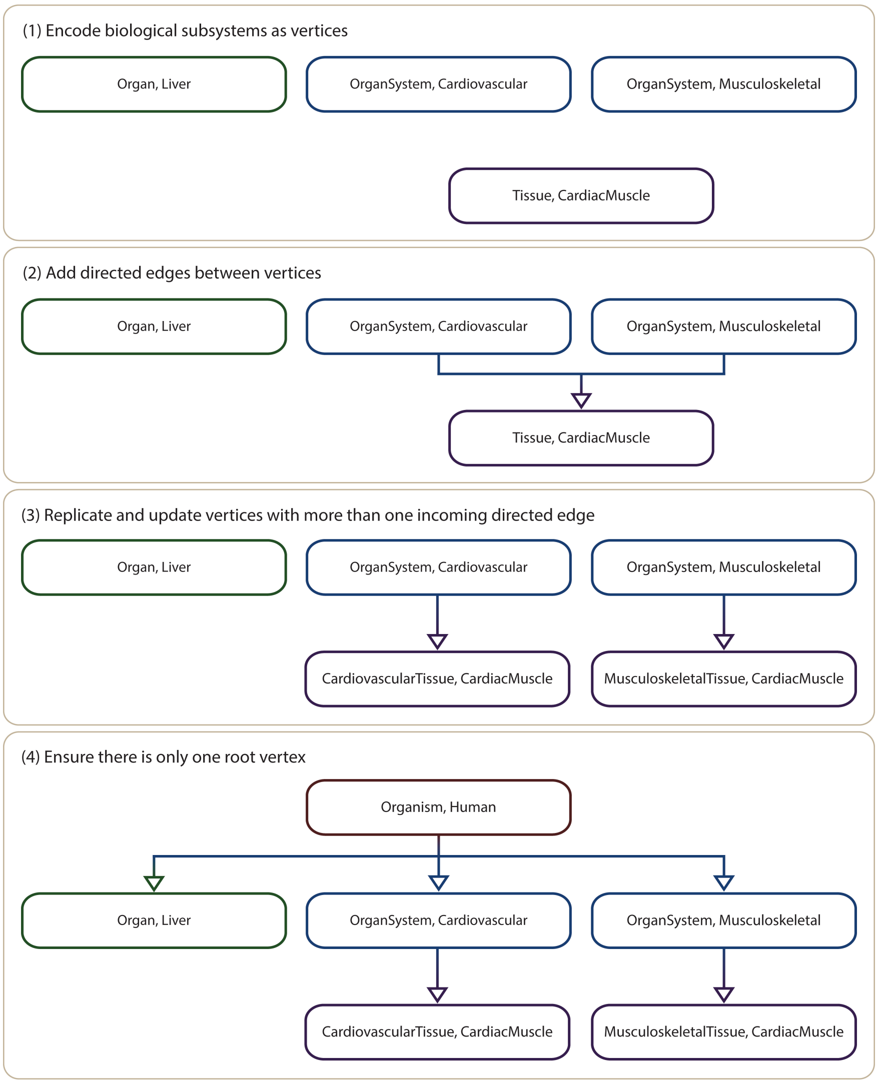


Figure 1: **Illustrative example on how to construct a multiscale architecture graph.** Let us assume that the biological subsystems under consideration are the human liver, the cardiac muscle tissue, and the cardiovascular and musculoskeletal organ systems. The first step (1) in constructing the $MA$ graph is to encode the biological subsystems and the associated scales as vertices (OrganSystem, Cardiovascular), (OrganSystem, Musculoskeletal), (Organ, Liver) and (Tissue, CardiacMuscle). Next (2) directed edges are added between each system and its constituent subsystems. The considered directed edges are ((OrganSystem, Cardiovascular), (Tissue, CardiacMuscle)) and ((OrganSystem, Musculoskeletal), (Tissue, CardiacMuscle)). Since vertices with multiple incoming directed edges are not allowed in rooted directed trees, the next step (3) is to duplicate the vertex (Tissue, CardiacMuscle) and rename each resulting instance according to its corresponding parent vertex. Therefore vertex (Tissue, CardiacMuscle) is replaced by the vertices (CardiovascularTissue, CardiacMuscle) and (MusculoskeletalTissue, CardiacMuscle), and the directed edges are updated accordingly. The last step (4) is to ensure that the $MA$ graph's root vertex is unique. For this purpose the (Organism, Human) vertex and the corresponding directed edges ((Organism, Human), (OrganSystem, Cardiovascular)), ((Organism, Human), (OrganSystem, Musculoskeletal)) and ((Organism, Human), (Organ, Liver)) are added to $MA$.
