# Supplementary material for: A Novel Method to Verify Multilevel Computational Models of Biological Systems Using Multiscale Spatio-Temporal Meta Model Checking
Source: PLoS One. 2016 May 17;11(5):e0154847. doi: 10.1371/journal.pone.0154847 (PMC4871515; doi:10.1371/journal.pone.0154847)
Supplement: S3 Text — (PDF) [file pone.0154847.s003.pdf]

# Description of the Multiscale Spatial Temporal Markup Language (MSTML)

The root element of an MSTML file is `experiment` and is used to record the time series data corresponding to an *in silico* model simulation, respectively *in vitro*/*in vivo* wet-lab experiment.

Irrespective of the nature of the experiment measurements are taken at one or multiple moments in time. Consequently the `experiment` root element contains one or more `timepoint` elements. Each `timepoint` element contains an optional *value* attribute which indicates the moment in time when the measurement was taken. The value of this attribute $value_{t_i}$ corresponding to time point $t_i$ is computed using the following formula:

$$value_{t_i} = \begin{cases} val, & \text{if the value } val \text{ was predefined for } t_i \\ 0, & \text{if no value was predefined for } t_i \text{ and } i = 0 \\ value_{t_{i-1}} + 1, & \text{otherwise} \end{cases}$$

The information stored in `timepoint` elements is a list of zero or more unique spatial entities (i.e. `spatialEntity` elements), respectively numeric state variables (i.e. `numericStateVariable` elements).

Both `spatialEntity` and `numericStateVariable` elements contain an optional attribute *scaleAndSubsystem* used to encode the scale and subsystem to which the spatial entity, respectively numeric state variable correspond.

However `spatialEntity` elements are additionally described by a required attribute *spatialType* whose value is either region or cluster. Information describing the state of the spatial entity at a given time point is recorded by the following child elements corresponding to the spatial measures considered during the multiscale spatio-temporal analysis:

- *clusteredness*, *density*, *triangleMeasure*, *rectangleMeasure* and *circleMeasure* - real non-negative values between 0 and 1;
- *angle* - a real non-negative value between 0 and 360;
- *area*, *perimeter*, *distanceFromOrigin*, *centroidX* and *centroidY* - real non-negative values.

Conversely `numericStateVariable` elements contain a `name` and a `value` child element where the name is a string and the value a real number.
