# Supplementary material for: A Novel Method to Verify Multilevel Computational Models of Biological Systems Using Multiscale Spatio-Temporal Meta Model Checking
Source: PLoS One. 2016 May 17;11(5):e0154847. doi: 10.1371/journal.pone.0154847 (PMC4871515; doi:10.1371/journal.pone.0154847)
Supplement: S4 Text — (PDF) [file pone.0154847.s004.pdf]

Formal definition of Bounded Linear Multiscale Spatial Temporal Logic (BLMSTL)

Syntax

The syntax of BLMSTL will be defined by a context-free grammar using the Backus-Naur Form (BNF) notation. A definition of a non-terminal symbol (element) in such grammars has the following form:

⟨*defined-element*⟩ ::= ⟨*element1*⟩
    | ⟨*element2*⟩
    | ...

where ::= introduces a new definition and | represents an alternative. In natural language this reads <defined−element> is either an <element1> or <element2> or (...).

Definition. *The* syntax *of BLMSTL is given by the following grammar expressed in BNF:*

⟨*logic-property*⟩ *::=* ⟨*temporal-numeric-measure*⟩ ⟨*comparator*⟩
    ⟨*temporal-numeric-measure*⟩
    | ⟨*change-measure*⟩*(*⟨*temporal-numeric-measure*⟩*)* ⟨*comparator*⟩
    ⟨*temporal-numeric-measure*⟩
    | ∼ ⟨*logic-property*⟩
    | ⟨*logic-property*⟩ ∧ ⟨*logic-property*⟩
    | ⟨*logic-property*⟩ ∨ ⟨*logic-property*⟩
    | ⟨*logic-property*⟩ ⇒ ⟨*logic-property*⟩
    | ⟨*logic-property*⟩ ⇔ ⟨*logic-property*⟩
    | ⟨*logic-property*⟩ *U[unsigned-real-number, unsigned-real-number]*
    ⟨*logic-property*⟩
    | *F[unsigned-real-number, unsigned-real-number]* ⟨*logic-property*⟩
    | *G[unsigned-real-number, unsigned-real-number]* ⟨*logic-property*⟩
    | *X* ⟨*logic-property*⟩
    | *X [*⟨*natural-number*⟩*]* ⟨*logic-property*⟩
    | *(*⟨*logic-property*⟩*)*

⟨*temporal-numeric-measure*⟩ *::=* ⟨*real-number*⟩
    | ⟨*numeric-state-variable*⟩
    | ⟨*numeric-statistical-measure*⟩
    | ⟨*unary-numeric-measure*⟩*(*⟨*temporal-numeric-measure*⟩*)*
    | ⟨*binary-numeric-measure*⟩*(*⟨*temporal-numeric-measure*⟩*,*
    ⟨*temporal-numeric-measure*⟩*)*

⟨*numeric-statistical-measure*⟩ *::=* ⟨*unary-statistical-measure*⟩*(*
    ⟨*numeric-measure-collection*⟩*)*
    | ⟨*binary-statistical-measure*⟩*(*⟨*numeric-measure-collection*⟩*,*
    ⟨*numeric-measure-collection*⟩*)*
    | ⟨*binary-statistical-quantile-measure*⟩*(*⟨*numeric-measure-collection*⟩*,*
    ⟨*real-number*⟩*)*

⟨*numeric-measure-collection*⟩ *::=* ⟨*spatial-measure-collection*⟩
    | *[unsigned-real-number,unsigned-real-number]* ⟨*numeric-measure*⟩

⟨*numeric-measure*⟩ *::=* ⟨*primary-numeric-measure*⟩
    | ⟨*unary-numeric-measure*⟩*(*⟨*numeric-measure*⟩*)*
    | ⟨*binary-numeric-measure*⟩*(*⟨*numeric-measure*⟩*,*⟨*numeric-measure*⟩*)*

⟨*primary-numeric-measure*⟩ *::=* ⟨*numeric-spatial-measure*⟩
    | ⟨*real-number*⟩
    | ⟨*numeric-state-variable*⟩

⟨*numeric-spatial-measure*⟩ *::=* ⟨*unary-statistical-measure*⟩*(*
        ⟨*spatial-measure-collection*⟩*)*
    | ⟨*binary-statistical-measure*⟩*(*⟨*spatial-measure-collection*⟩*,*
        ⟨*spatial-measure-collection*⟩*)*
    | ⟨*binary-statistical-quantile-measure*⟩*(*⟨*spatial-measure-collection*⟩*,*
        ⟨*real-number*⟩*)*

⟨*unary-statistical-measure*⟩ *::=* *avg*
    | *count*
    | *geomean*
    | *harmean*
    | *kurt*
    | *max*
    | *median*
    | *min*
    | *mode*
    | *product*
    | *skew*
    | *stdev*
    | *sum*
    | *var*

⟨*binary-statistical-measure*⟩ *::=* *covar*

⟨*binary-statistical-quantile-measure*⟩ *::=* *percentile*
    | *quartile*

⟨*unary-numeric-measure*⟩ *::=* *abs*
    | *ceil*
    | *floor*
    | *round*
    | *sign*
    | *sqrt*
    | *trunc*

⟨*binary-numeric-measure*⟩ *::=* *add*
    | *div*
    | *log*
    | *mod*
    | *multiply*

| *power*
| *subtract*

⟨*spatial-measure-collection*⟩ ::= ⟨*spatial-measure*⟩(⟨*subset*⟩)
| ⟨*unary-numeric-measure*⟩(⟨*spatial-measure-collection*⟩)
| ⟨*binary-numeric-measure*⟩(⟨*spatial-measure-collection*⟩, ⟨*spatial-measure-collection*⟩)

⟨*spatial-measure*⟩ ::= *clusteredness*
| *density*
| *area*
| *perimeter*
| *distanceFromOrigin*
| *angle*
| *triangleMeasure*
| *rectangleMeasure*
| *circleMeasure*
| *centroidX*
| *centroidY*

⟨*subset*⟩ ::= ⟨*subset-specific*⟩
| *filter*(⟨*subset-specific*⟩,⟨*constraint*⟩)
| ⟨*subset-operation*⟩(⟨*subset*⟩,⟨*subset*⟩)

⟨*subset-specific*⟩ ::= *regions*
| *clusters*

⟨*constraint*⟩ ::= *scaleAndSubsystem* ⟨*comparator*⟩ ⟨*scale-and-subsystem*⟩
| ⟨*spatial-measure*⟩ ⟨*comparator*⟩ ⟨*filter-numeric-measure*⟩
| $\sim$ ⟨*constraint*⟩
| ⟨*constraint*⟩ $\wedge$ ⟨*constraint*⟩
| ⟨*constraint*⟩ $\vee$ ⟨*constraint*⟩
| ⟨*constraint*⟩ $\Rightarrow$ ⟨*constraint*⟩
| ⟨*constraint*⟩ $\Leftrightarrow$ ⟨*constraint*⟩
| (⟨*constraint*⟩)

⟨*filter-numeric-measure*⟩ ::= ⟨*primary-numeric-measure*⟩
| ⟨*spatial-measure*⟩
| ⟨*unary-numeric-measure*⟩(⟨*filter-numeric-measure*⟩)
| ⟨*binary-numeric-measure*⟩(⟨*filter-numeric-measure*⟩, ⟨*filter-numeric-measure*⟩)

⟨*subset-operation*⟩ ::= *difference*
| *intersection*
| *union*

⟨*change-measure*⟩ ::= *d*
| *r*

⟨*real-number*⟩ ::= ⟨*unsigned-real-number*⟩
| ⟨*sign*⟩ ⟨*unsigned-real-number*⟩

⟨*unsigned-real-number*⟩ *::=* ⟨*fractional-part*⟩
| ⟨*fractional-part*⟩ ⟨*exponent-part*⟩

⟨*fractional-part*⟩ *::=* ⟨*digit-sequence*⟩ *.* ⟨*digit-sequence*⟩
| *.* ⟨*digit-sequence*⟩
| ⟨*digit-sequence*⟩ *.*
| ⟨*digit-sequence*⟩

⟨*digit-sequence*⟩ *::=* ⟨*digit*⟩
| ⟨*digit*⟩ ⟨*digit-sequence*⟩

⟨*digit*⟩ *::=* *0*
| *1*
| *2*
| *3*
| *4*
| *5*
| *6*
| *7*
| *8*
| *9*

⟨*natural-number*⟩ *::=* ⟨*digit-sequence*⟩
| *+* ⟨*digit-sequence*⟩

⟨*exponent-part*⟩ *::=* *e* ⟨*digit-sequence*⟩
| *E* ⟨*digit-sequence*⟩
| *e* ⟨*sign*⟩ ⟨*digit-sequence*⟩
| *E* ⟨*sign*⟩ ⟨*digit-sequence*⟩

⟨*sign*⟩ *::=* *+*
| *-*

⟨*comparator*⟩ *::=* *<*
| *<=*
| *=*
| *>=*
| *>*

⟨*numeric-state-variable*⟩ *::=*
⟨*state-variable*⟩ ⟨*state-variable-scale-and-subsystem*⟩

⟨*state-variable*⟩ *::=* {⟨*string*⟩}

⟨*state-variable-scale-and-subsystem*⟩ *::=* ϵ
| *(scaleAndSubsystem =* ⟨*scale-and-subsystem*⟩*)*

⟨*string*⟩ *::=* ⟨*character*⟩ | ⟨*character*⟩ ⟨*string*⟩

⟨*character*⟩ *::=* *based on the Unicode character set except "{" and "}"*

⟨*scale-and-subsystem*⟩ *::=* ⟨*primary-scale-and-subsystem*⟩ *.*
⟨*primary-scale-and-subsystem*⟩

⟨*primary-scale-and-subsystem*⟩ *::=* ⟨*scale-and-subsystem-character*⟩
| ⟨*scale-and-subsystem-character*⟩ ⟨*primary-scale-and-subsystem*⟩

⟨*scale-and-subsystem-character*⟩ *::=* ⟨*basic-latin-script-character*⟩
| ⟨*digit*⟩

⟨*basic-latin-script-character*⟩ *::=* *a* | *b* | *c* | *d* | *e* | *f* | *g* | *h* | *i* | *j* | *k* | *l* | *m* | *n* | *o* | *p* | *q* | *r* | *s* | *t* | *u* | *v* | *w* | *x* | *y* | *z* | *A* | *B* | *C* | *D* | *E* | *F* | *G* | *H* | *I* | *J* | *K* | *L* | *M* | *N* | *O* | *P* | *Q* | *R* | *S* | *T* | *U* | *V* | *W* | *X* | *Y* | *Z*

The order of precedence of the operators is given by the definition of the BLMSTL syntax. In the absence of parentheses the logic expressions are evaluated from left to right.

Semantics

The semantics of BLMSTL is defined with respect to executions of an MSSpDES $\mathcal{M}$

$$\sigma = \{(s_0, t_0), (s_1, t_1), ...\}$$

where s_0, s_1, ... represent the states of the execution and t_0, t_1, ... $\in \mathbb{R}_+$ the time durations spent in each corresponding state. Throughout it is assumed that execution traces are time-divergent which means that only a finite number of transitions can occur in a finite amount of time. Given an execution trace $\sigma = \{(s_0, t_0), (s_1, t_1), ...\}$, a time value $t \in \mathbb{R}_+$ and a natural number i, the length of (or the number of states in) the execution trace is denoted by $|\sigma|$, the i-th state of the execution trace by $\sigma[i]$, the execution trace suffix starting at the i-th state by σ^i, the execution trace suffix starting after t time by $\sigma(t) = \sigma^i$, where $i \in \mathbb{N}$ is the minimum index such that $t \leq \sum_{j=0}^{i} t_j$, and the fact that the execution σ satisfies a property ϕ by $\sigma \models \phi$. For an execution trace σ at state s the value of a numeric state variable nsv is given by $NV(s, nsv)$ and its associated scale and subsystem by $SVSS(nsv)$, respectively the value of a spatial state variable $spsv$ is given by $SpV(s, spsv)$ and its associated scale and subsystem by $SVSS(spsv)$.

In order to have a compact and easy to follow semantics description the full symbol names specific to BLMSTL were replaced with shorter abbreviations as described in Table 1.

Definition. *Let* $\mathcal{M} = \langle S,\ T,\ \mu,\ NSV,\ SpSV,\ NV,\ CSpV,\ MA,\ SVSS \rangle$ *be an MSSpDES and* σ *an execution of* $\mathcal{M}$*. The* semantics *of BLMSTL for* σ *is defined as follows:*

- $\sigma \models tnm_1 \asymp tnm_2$ **if and only if** $tnm_1(\sigma) \asymp tnm_2(\sigma)$, *where* $tnm_1(\sigma)$ *and* $tnm_2(\sigma) \in \mathbb{R}$, *and* $\asymp \in \{<, <=, =, >=, >\}$;
- $\sigma \models cm(tnm_1) \asymp tnm_2$ **if and only if** $|\sigma| > 1$ *and* $cm(tnm_1, \sigma) \asymp tnm_2(\sigma)$, *where* $cm(tnm_1, \sigma)$, $tnm_2(\sigma) \in \mathbb{R}$, *and* $\asymp \in \{<, <=, =, >=, >\}$;

Table 1: Translation of full BLMSTL symbol names to abbreviated forms. The left column contains the full BLMSTL symbol name. The right column contains the corresponding abbreviated form.

Full BLMSTL symbol name	Abbreviated BLMSTL symbol name
<logic-property>	ψ
<temporal-numeric-measure>	*tnm*
<numeric-statistical-measure>	*nstm*
<numeric-measure-collection>	*nmc*
<numeric-measure>	*nm*
<primary-numeric-measure>	*pnm*
<numeric-spatial-measure>	*nsm*
<unary-statistical-measure>	*usm*
<binary-statistical-measure>	*bsm*
<binary-statistical-quantile-measure>	*bsqm*
<unary-numeric-measure>	*unm*
<binary-numeric-measure>	*bnm*
<spatial-measure-collection>	*smc*
<spatial-measure>	*sm*
<subset>	*ss*
<filter-numeric-measure>	*fnm*
<comparator>	$\asymp$
<change-measure>	*cm*
<real-number>	*re*
<scale-and-subsystem>	*scsubsys*
<state-variable>	*sv*
<numeric-state-variable>	*nsv*
<spatial-state-variable>	*spsv*

- $\sigma \models \sim \psi$ **if and only if** $\sigma \not\models \psi$*;*
- $\sigma \models \psi_1 \wedge \psi_2$ **if and only if** $\sigma \models \psi_1$ *and* $\sigma \models \psi_2$*;*
- $\sigma \models \psi_1 \vee \psi_2$ **if and only if** $\sigma \models \psi_1$ *or* $\sigma \models \psi_2$*;*
- $\sigma \models \psi_1 \Rightarrow \psi_2$ **if and only if** $\sigma \models \sim \psi_1$ *or* $\sigma \models \psi_2$*;*
- $\sigma \models \psi_1 \Leftrightarrow \psi_2$ **if and only if** $\sigma \models \psi_1 \Rightarrow \psi_2$ *and* $\sigma \models \psi_2 \Rightarrow \psi_1$*;*
- $\sigma \models \psi_1\ U[a,b]\ \psi_2$ **if and only if** *there exists* i*,* $i \in [a,b]$*, such that* $\sigma(i) \models \psi_2$*, and for all* j*,* $j \in [a,i)$*, it holds that* $\sigma(j) \models \psi_1$ *with* $a, b \in \mathbb{R}_+$*;*
- $\sigma \models F[a,b]\ \psi$ **if and only if** *there exists* i*,* $i \in [a,b]$*, such that* $\sigma(i) \models \psi$ *with* $a, b \in \mathbb{R}_+$*;*
- $\sigma \models G[a,b]\ \psi$ **if and only if** *for all* i*,* $i \in [a,b]$*, it holds that* $\sigma(i) \models \psi$ *with* $a, b \in \mathbb{R}_+$*;*
- $\sigma \models X\ \psi$ **if and only if** $|\sigma| > 1$ *and* $\sigma^1 \models \psi$*;*
- $\sigma \models X[k]\ \psi$ **if and only if** $|\sigma| > k$ *and* $\sigma^k \models \psi$ *with* $k \in \mathbb{N}$*;*

- $\sigma \models (\psi)$ **if and only if** $\sigma \models \psi$.

An illustrative example of how to employ logic properties to encode the expected behaviour of a system is given below both in natural language and BLMSTL:

> **Natural language:** Throughout the time interval [0, 100] the concentration of protein A is expected to always increase;
> **BLMSTL:** G [0, 100] $(d(\{A\}) > 0)$.

The *tnm* symbol represents the category of temporal numeric measures. Considering a model execution σ, $tnm(\sigma)$ is evaluated to a real number based on one of the definitions below:

- **Real number:** if $tnm = re$, then $tnm(\sigma) = re \in \mathbb{R}$;
- **Numeric state variable:** if $tnm = nsv$, then $tnm(\sigma) = nsv(\sigma)$;
- **Numeric statistical measure:** if $tnm = nstm$, then $tnm(\sigma) = nstm(\sigma)$;
- **Unary numeric measure:** if $tnm = unm(tnm')$, where unm is a unary numeric measure and tnm' is a temporal numeric measure, then $tnm(\sigma) = unm(tnm'(\sigma))$ such that $tnm'(\sigma) \in \mathbb{R}$;
- **Binary numeric measure:** if $tnm = bnm(tnm', tnm'')$, where bnm is a binary numeric measure, and tnm' and tnm'' are temporal numeric measures, then $tnm(\sigma) = bnm(tnm'(\sigma), tnm''(\sigma))$ such that $tnm'(\sigma), tnm''(\sigma) \in \mathbb{R}$.

The values of the *unary* (i.e. unm) and *binary* (i.e. bnm) *numeric measures* considering one (i.e. $unm(re)$), respectively two real numbers (i.e. $bnm(re', re'')$) are computed as described in Appendix A (Tables 2, respectively 3).

An illustrative example of how to employ temporal numeric measures, unary numeric measures and binary numeric measures to encode the expected behaviour of a system is given below both in natural language and BLMSTL:

> **Natural language:** At some point within the time interval [0, 500] the (absolute value of the) difference between the concentration of chemical X and chemical Y is less than 20 M;
> **BLMSTL:** F [0, 500] $(abs(subtract(\{X\}, \{Y\})) < 20)$.

The category of numeric statistical measures is represented by the *nstm* symbol. For a given model execution σ, $nstm(\sigma)$ is evaluated to a real number considering one of the definitions below:

- **Unary statistical numeric measure:** if $nstm = usm(nmc)$, where usm is a unary statistical measure and nmc is a numeric measure collection, then $nstm(\sigma) = usm(nmc(\sigma))$ such that $nmc(\sigma)$ represents a collection of real values;
- **Binary statistical numeric measure:** if $nstm = bsm(nmc', nmc'')$, where bsm is a binary statistical measure, and nmc' and nmc'' are numeric measure collections, then $nstm(\sigma) = bsm(nmc'(\sigma), nmc''(\sigma))$ such that $nmc'(\sigma)$ and $nmc''(\sigma)$ represent collections of real values;

- **Binary statistical quantile numeric measure:** if $nstm = bsqm(nmc, re)$, where $bsqm$ is a binary statistical quantile measure, nmc is a numeric measure collection and re is a real number, then $nstm(\sigma) = bsqm(nmc(\sigma), re)$ such that $nmc(\sigma)$ represents a collection of real values.

The value of *unary statistical* (i.e. usm), *binary statistical* (i.e. bsm) and *binary statistical quantile* (i.e. $bsqm$) *measures* considering a collection of real values (i.e. $usm(nmc)$), respectively two collections of real values (i.e. $bsm(nmc', nmc'')$) or a collection of real values and a real number (i.e. $bsqm(nmc, re)$) is computed as described in Appendix B (Tables 4, 5, respectively 6). In case the numeric measure collections considered contain insufficient elements the corresponding numeric statistical measures are evaluated to zero.

The nmc symbol represents numeric measure collections. Given a model simulation σ, $nmc(\sigma)$ is evaluated to a collection of real values according to one of the following definitions:

- **Spatial measure collection:** if $nmc = smc$, then $nmc(\sigma) = smc(\sigma)$;
- **Temporal numeric measure collection:** if $nmc = [a, b]\ nm$, where nm is a numeric measure, and a and b are real numbers, then $nmc(\sigma) = \{nm(\sigma(i)) \mid nm(\sigma(i)) \in \mathbb{R}$ is the result of evaluating the numeric measure nm against the subtrace $\sigma(i)$, for all $i \in [a, b]$ with $a, b \in \mathbb{R}_+\}$.

An illustrative example of how to employ numeric measure collections and unary statistical measures to encode the expected behaviour of a system is given below both in natural language and BLMSTL:

> **Natural language:** Considering the time interval [100, 200] the maximum upper value of the blood pressure corresponding to scale organism and subsystem human is less than 150;
> **BLMSTL:** $max([100, 200]\ \{bloodPressureUpperValue\}$ $(scaleAndSubsystem = Organism.Human)) < 150$.

The nm symbol represents the category of real-valued numeric measures. Considering a given execution σ, $nm(\sigma)$ is evaluated to a real number according to one of the definitions described below:

- **Primary numeric measure:** if $nm = pnm$, then $nm(\sigma) = pnm(\sigma)$;
- **Unary numeric measure:** if $nm = unm(nm')$, where unm is a unary numeric measure and nm' is a numeric measure, then $nm(\sigma) = unm(nm'(\sigma))$ such that $nm'(\sigma) \in \mathbb{R}$;
- **Binary numeric measure:** if $nm = bnm(nm', nm'')$, where bnm is a binary numeric measure, and nm' and nm'' are numeric measures, then $nm(\sigma) = bnm(nm'(\sigma), nm''(\sigma))$ such that $nm'(\sigma), nm''(\sigma) \in \mathbb{R}$.

The pnm symbol corresponds to primary numeric (real-valued) measures. Considering a given execution σ, $pnm(\sigma)$ is evaluated to a real number according to one of the following definitions:

- **Numeric spatial measure:** if $nm = nsm$, then $nm(\sigma) = nsm(\sigma)$;
- **Real number:** if $nm = re$, then $nm(\sigma) = re \in \mathbb{R}$;

- **Numeric state variable:** if $nm = nsv$, then $nm(\sigma) = nsv(\sigma)$.

The nsm symbol represents the category of numeric (real-valued) spatial measures. Considering a given execution σ, $nsm(\sigma)$ is evaluated to a real number according to one of the definitions described below:

- **Unary statistical spatial measure:** if $nsm = usm(smc)$, where usm is a unary statistical measure and smc is a spatial measure collection, then $nsm(\sigma) = usm(smc(\sigma))$ such that $smc(\sigma)$ represents a collection of real values;

- **Binary statistical spatial measure:** if $nsm = bsm(smc', smc'')$, where bsm is a binary statistical measure, and smc' and smc'' are spatial measure collections, then $nsm(\sigma) = bsm(smc'(\sigma), smc''(\sigma))$ such that $smc'(\sigma)$ and $smc''(\sigma)$ represent collections of real values;

- **Binary statistical quantile spatial measure:** if $nsm = bsqm(smc, re)$, where $bsqm$ is a binary statistical quantile measure, smc is a spatial measure collection and re is a real number, then $nsm(\sigma) = bsqm(smc(\sigma), re)$ such that $smc(\sigma)$ represents a collection of real values.

The smc symbol represents spatial measure collections. Considering a model execution σ, $smc(\sigma)$ is evaluated to a collection of real values using one of the definitions below:

- **Primary spatial measure collection:** if $smc = sm(ss)$, where sm is a spatial measure and ss is the subset of considered spatial entities, then $smc(\sigma) = \{sm_{value} \mid sm_{value} \in \mathbb{R}$ is the result of evaluating the spatial measure sm for all spatial entities in $ss(\sigma)\}$;

- **Unary numeric spatial measure collection:** if $smc = unm(smc')$, where unm is a unary numeric measure and smc' is a spatial measure collection, then $smc(\sigma) = unm(smc'(\sigma))$, where $unm(smc'(\sigma)) = \{value$ $\mid value = unm(smc_{value})$ for all $smc_{value} \in smc'(\sigma)\}$;

- **Binary numeric spatial measure collection:** if $smc = bnm(smc',$ $smc'')$, where bnm is a binary numeric measure, and smc' and smc'' are spatial measure collections, then $smc(\sigma) = bnm(smc'(\sigma), smc''(\sigma))$, where $bnm(smc'(\sigma), smc''(\sigma)) = \{value \mid value = bnm(smc'_{value}, smc''_{value})$ where smc'_{value} and smc''_{value} are the i-th values in $smc'(\sigma)$, respectively $smc''(\sigma)$ for all i, $1 \leq i \leq \min(|smc'(\sigma)|, |smc''(\sigma)|)\}$.

Spatial measures sm are defined over the set {clusteredness, density, area, perimeter, distanceFromOrigin, angle, triangleMeasure, rectangleMeasure, circleMeasure, centroidX, centroidY} which is identical to the set of spatial measures computed for each detected region/cluster during the multiscale spatio-temporal analysis step of the model checking workflow.

Subsets of spatial entities are represented by the ss symbol. Considering a given execution σ, $ss(\sigma)$ is evaluated to a collection of spatial entities according to one of the definitions described below:

- **Specific subset:** if $ss = specificSubset$, then $ss(\sigma) = specificSubset(\sigma)$, where $specificSubset(\sigma)$ represents the collection of all clusters/regions corresponding to $\sigma[0]$;

- **Filtered specific subset:** if $ss = \mathit{filter}(\mathit{specificSubset}, \mathit{constraint})$, where *specificSubset* has the semantics defined above and *constraint* is a logic property for encoding constraints on spatial measure values (e.g. area > 10), then $ss(\sigma) = \mathit{filter}(\mathit{specificSubset}(\sigma), \mathit{constraint})$;

- **Subset operation result:** if $ss = \mathit{subsetOperation}(ss', ss'')$, where ss' and ss'' are subsets of spatial entities, and *subsetOperation* is a subset operation, then $ss(\sigma) = \mathit{subsetOperation}(ss'(\sigma),\ ss''(\sigma))$ such that $ss'(\sigma)$ and $ss''(\sigma)$ represent collections of spatial entities.

Given an execution σ the value of $\mathit{specificSubset}(\sigma)$ is computed using one of the definitions described below:

- **Regions:** if $\mathit{specificSubset} = \mathit{regions}$, then $\mathit{specificSubset}(\sigma) = \bigcup_{spsv \in SpSV}$ $\{\mathit{region} \mid \mathit{region} \in \text{regionsDetectionMechanism}(\mathit{SpV}(\sigma[0],\ \mathit{spsv}))$ such that $\mathit{SpV} \in \mathit{CSpV}$ is defined for state $\sigma[0]$ and spatial state variable $\mathit{spsv}\}$;

- **Clusters:** if $\mathit{specificSubset} = \mathit{clusters}$, then $\mathit{specificSubset}(\sigma) = \text{clustersDetectionMechanism}(\mathit{reg})$, where $\mathit{reg} = \bigcup_{spsv \in SpSV} \{\mathit{region} \mid$ $\mathit{region} \in \text{regionDetectionMechanism}(\mathit{SpV}(\sigma[0],\ \mathit{spsv}))$ such that $\mathit{SpV} \in \mathit{CSpV}$ is defined for state $\sigma[0]$ and spatial state variable $\mathit{spsv}\}$.

An illustrative example of how to employ specific subsets of spatial entities and unary statistical measures to encode the expected behaviour of a system is given below both in natural language and BLMSTL:

> **Natural language:** During the time interval [50.3, 112.7] the average area of the regions corresponding to scale tissue and subsystem cancerous liver tissue is increasing until it reaches a value greater than 2400;
> **BLMSTL:** $(d(\mathit{avg}(\mathit{area}(\mathit{filter}(\mathit{regions},\ \mathit{scaleAndSubsystem} =$
> $\mathit{Tissue.CancerousLiverTissue})))) > 0)\ U\ [50.3,\ 112.7]$
> $(\mathit{avg}(\mathit{area}(\mathit{filter}(\mathit{regions},\ \mathit{scaleAndSubsystem} =$
> $\mathit{Tissue.CancerousLiverTissue}))) > 2400)$.

Subsets of spatial entities collections recorded by *specificSubset* can be computed using the *filter* predicate. Considering an execution σ, a collection of spatial entities *specificSubset* and a constraint on spatial measure values *constraint*, $\mathit{filter}(\mathit{specificSubset}(\sigma), \mathit{constraint})$ is evaluated to a collection of spatial entities using the definition described below:

$$\mathit{filter}(\mathit{specificSubset}(\sigma), \mathit{constraint}) = \{se \mid se \text{ is a spatial entity in } \mathit{specificSubset}(\sigma) \text{ which satisfies } \mathit{constraint} \text{ i.e. } se \models \mathit{constraint}\}.$$

The semantics of the constraint satisfaction problem considering an execution σ, a spatial entity *se* and a constraint *constraint* is defined as follows:

- if $\mathit{constraint} = \mathit{scaleAndSubsystem} \asymp \mathit{scsubsys}$, then $se \models \mathit{scaleAndSubsystem} \asymp \mathit{scsubsys}$ **if and only if** there exists a vertex $v_{scsubsys} \in V_{MA}$ encoding the scale and subsystem *scsubsys*, *se* was annotated with a scale and subsystem $se_{scaleAndSubsystem}$ which has a corresponding vertex $v_{se_{scaleAndSubsystem}} \in V_{MA}$, and $v_{se_{scaleAndSubsystem}} \asymp$

$v_{scsubsys}$, where V_{MA} is the set of vertices in the multiscale architecture graph MA, and $\asymp \in \{<, <=, =, >=, >\}$. To determine the truth value of expressions of the form $v_{se_{scaleAndSubsystem}} \asymp v_{scsubsys}$ the partial orders $<$ and $\leq$ defined over the set of vertices V_{MA} are considered. Therefore $v_{se_{scaleAndSubsystem}} < v_{scsubsys}$ holds if the path from the root of MA to $v_{se_{scaleAndSubsystem}}$ passes through $v_{scsubsys}$. Conversely $v_{se_{scaleAndSubsystem}} > v_{scsubsys}$ holds if the path from the root of MA to $v_{scsubsys}$ passes through $v_{se_{scaleAndSubsystem}}$. Expressions $v_{se_{scaleAndSubsystem}} \leq v_{scsubsys}$ and $v_{se_{scaleAndSubsystem}} \geq v_{scsubsys}$ hold if $v_{se_{scaleAndSubsystem}} < v_{scsubsys}$ or $v_{se_{scaleAndSubsystem}} = v_{scsubsys}$, respectively if $v_{se_{scaleAndSubsystem}} > v_{scsubsys}$ or $v_{se_{scaleAndSubsystem}} = v_{scsubsys}$.

- if $constraint = sm \asymp fnm$, then $se \models sm \asymp fnm$ **if and only if** $sm(se) \asymp fnm(se, \sigma)$, where $sm(se)$ evaluates the spatial measure sm for the given spatial entity se, $sm \in$ {clusteredness, density, area, perimeter, distanceFromOrigin, angle, triangleMeasure, rectangleMeasure, circleMeasure, centroidX, centroidY}, $fnm(se, \sigma) \in \mathbb{R}$ represents a real number obtained by evaluating the filter numeric measure fnm against se and σ, and $\asymp \in \{<, <=, =, >=, >\}$.

- if $constraint = \sim constraint_1$, then $se \models \sim constraint_1$ **if and only if** $se \not\models constraint_1$.

- if $constraint = constraint_1 \wedge constraint_2$, then $se \models constraint_1 \wedge constraint_2$ **if and only if** $se \models constraint_1$ and $se \models constraint_2$.

- if $constraint = constraint_1 \vee constraint_2$, then $se \models constraint_1 \vee constraint_2$ **if and only if** $se \models constraint_1$ or $se \models constraint_2$.

- if $constraint = constraint_1 \Rightarrow constraint_2$, then $se \models constraint_1 \Rightarrow constraint_2$ **if and only if** $se \models \sim constraint_1$ or $se \models constraint_2$.

- if $constraint = constraint_1 \Leftrightarrow constraint_2$, then $se \models constraint_1 \Leftrightarrow constraint_2$ **if and only if** $se \models constraint_1 \Rightarrow constraint_2$ and $se \models constraint_2 \Rightarrow constraint_1$.

- if $constraint = (constraint)$, then $se \models (constraint)$ **if and only if** $se \models constraint$.

The *fnm* symbol represents the (real-valued) numeric measure computed for the *filter* predicate. Given a spatial entity *se* and an execution σ, $fnm(se, \sigma)$ is evaluated to a real number using one of the definitions given below:

- **Primary numeric measure:** if $fnm = pnm$, then $fnm(se, \sigma) = pnm(\sigma)$;

- **Spatial measure:** if $fnm = sm$, where $sm \in$ {clusteredness, density, area, perimeter, distanceFromOrigin, angle, triangleMeasure, rectangleMeasure, circleMeasure, centroidX, centroidY}, then $fnm(se, \sigma) = sm(se)$;

- **Unary filter numeric measure:** if $fnm = unm(fnm')$, where unm is a unary numeric measure and fnm' is a filter numeric measure, then $fnm(se, \sigma) = unm(fnm'(se, \sigma))$ such that $fnm'(se, \sigma) \in \mathbb{R}$;

- **Binary filter numeric measure:** if $fnm = bnm(fnm', fnm'')$, where bnm is a binary numeric measure, and fnm' and fnm'' are filter numeric measures, then $fnm(se,\sigma) = bnm(fnm'(se,\sigma), fnm''(se,\sigma))$ such that $fnm'(se,\sigma), fnm''(se,\sigma) \in \mathbb{R}$.

The $subsetOperation \in \{\text{difference, intersection, union}\}$ symbol represents the collection of operations defined over spatial entities subsets. Given two spatial entities subsets ss' and ss'', $subsetOperation(ss', ss'')$ is evaluated to a collection of spatial entities using one of the following definitions:

- **Difference:** if $subsetOperation = difference$, then $subsetOperation(ss',\, ss'') = \{se \mid se \in ss' \text{ and } se \notin ss''\}$;
- **Intersection:** if $subsetOperation = intersection$, then $subsetOperation(ss',\, ss'') = \{se \mid se \in ss' \text{ and } se \in ss''\}$;
- **Union:** if $subsetOperation = union$, then $subsetOperation(ss',\, ss'') = \{se \mid se \in ss' \text{ or } se \in ss''\}$.

An illustrative example of how to employ subset operations and multiple filtered subsets of spatial entities to encode the expected behaviour of a system is given below both in natural language and BLMSTL:

> **Natural language:** The number of clusters of cells having the area greater than 20 or cellular regions having the area smaller than 50 is greater than 0;
> **BLMSTL:** $count(area(union(filter(clusters,\, area > 20), filter(regions,\, area < 50)))) > 0$.

The $cm \in \{d,\, r\}$ symbol represents the collection of measures which compute the rate at which the value of a temporal numeric measure tnm changes from the current state to the next. Considering a temporal numeric measure tnm and a given execution σ, such that $|\sigma| > 1$, $cm(tnm, \sigma)$ is evaluated to a real number according to one of the definitions below:

- **Derivative**: if $cm = d$, then $cm(tnm,\sigma) = d(tnm,\sigma)$, where

$$d(tnm,\sigma) = \frac{tnm(\sigma^1) - tnm(\sigma^0)}{t_0} \;;$$

- **Ratio**: if $cm = r$, then $cm(tnm,\sigma) = r(tnm,\sigma)$, where

$$r(tnm,\sigma) = \frac{\frac{tnm(\sigma^1)}{tnm(\sigma^0)}}{t_0} \;,$$

where t_0 represents the time duration spent in state $\sigma[0]$.

Finally the symbol nsv represents real-valued numeric state variables. Given an execution σ, $nsv(\sigma)$ is evaluated to a real number according to one of the following definitions:

- **Numeric state variable without an associated scale and subsystem:** if $nsv = sv$, where sv is the identifier of a numeric state variable having no associated scale and subsystem (i.e. $SVSS(sv)$ is not defined), then $nsv(\sigma) = NV(\sigma[0], sv)$;

- **Numeric state variable with an associated scale and subsystem:** if $nsv = sv(scaleAndSubsystem = scsubsys)$, where sv is the identifier of a numeric state variable having the associated scale and subsystem $scsubsys$, then $nsv(\sigma) = NV(\sigma[0], sv)$ and $SVSS(sv) = v_{scsubsys}$ such that $v_{scsubsys} \in V_{MA}$ corresponds to the scale and subsystem $scsubsys$.

An illustrative example of how to employ change measures and numeric state variables to encode the expected behaviour of a system is given below both in natural language and BLMSTL:

> **Natural language:** Throughout the time interval [20.1, 20.5] the concentration of calcium doubles from one time point to the next relative to the duration spent in each state;
> **BLMSTL:** G [20.1, 20.5] $(r(\{Calcium\}) = 2)$.

Appendices

A Numeric measures

Table 2: Name, description and semantics of unary numeric measures

Name	Description	Semantics
abs	Returns the absolute value of a number n	$abs(n) = \|n\|$
ceil	Rounds the number n upward, returning the smallest integral value that is not less than n	$ceil(n) = \lceil n \rceil$
floor	Rounds the number n downward, returning the largest integral value that is not greater than n	$floor(n) = \lfloor n \rfloor$
round	Returns the integral value that is nearest to number n, with halfway cases rounded away from zero	$round(n) = \begin{cases} \lfloor n + 0.5 \rfloor, & \text{if } n \geq 0 \\ \lceil n - 0.5 \rceil, & \text{otherwise} \end{cases}$
sign	Returns the sign of a number n	$sign(n) = \begin{cases} 1, & \text{if } n > 0 \\ 0, & \text{if } n = 0 \\ -1, & \text{otherwise} \end{cases}$
sqrt	Returns the square root of a number n	$sqrt(n) = \sqrt{n}$
trunc	Rounds number n toward zero	$trunc(n) = sign(n) \lfloor \|n\| \rfloor$

Table 3: Name, description and semantics of binary numeric measures

Name	Description	Semantics
add	Returns the sum of two numbers n_1 and n_2	$add(n_1, n_2) = n_1 + n_2$
div	Returns the integer part of the division n_1/n_2	$div(n_1, n_2) = \lfloor n_1/n_2 \rfloor$
log	Returns the logarithm of a number n in the given base b	$log(n, b) = \log_b (\|n\|)$, $n > 0$, $b > 0$, $b \neq 1$

mod	Returns the remainder of the division n_1/n_2	$mod(n_1, n_2) = n_1 - (n_2 \times div(n_1, n_2))$
multiply	Returns the multiplication of two numbers n_1 and n_2	$multiply(n_1, n_2) = n_1 \times n_2$
power	Returns the base b raised at the power e	$pow(b, e) = b^e$
subtract	Returns the difference between two numbers n_1 and n_2	$subtract(n_1, n_2) = n_1 - n_2$

B Statistical measures

Table 4: Name, description and semantics of unary statistical measures

**Name**	**Description**	**Semantics**
avg	Returns the arithmetic mean for the considered collection of *values*	$avg(values) = \frac{1}{n}\sum_{i=1}^{n}(values_i)$, where $n = \|values\|$
count	Returns the number of elements in the considered collection of *values*	$count(values) = \|values\|$
geomean	Returns the geometric mean for the considered collection of *values*	$geomean(values) = \left(\prod_{i=1}^{n}(values_i)\right)^{\frac{1}{n}}$, where $n = \|values\|$
harmean	Returns the harmonic mean for the considered collection of *values*	$harmean(values) = \frac{n}{\sum_{i=1}^{n}\frac{1}{(values_i)}}$, where $n = \|values\|$
kurt	Returns the kurtosis for the considered collection of *values*	$kurt(values) = \frac{n(n+1)}{(n-1)(n-2)(n-3)} \sum_{i=1}^{n}\left(\frac{(values_i)-avg(values)}{stdev(values)}\right)^4 - \frac{3(n-1)^2}{(n-2)(n-3)}$, where $n = \|values\|$ and $n \geq 4$
max	Returns the maximum for the considered collection of *values*	$max(values) = \max_{i=\overline{1,n}}(values_i)$, where $n = \|values\|$

median	Returns the median for the considered collection of *values*	$median(values)$ = middle element from position $\lfloor \lvert values \rvert / 2 \rfloor$ (0-based indexing) in the ordered list of *values*
min	Returns the minimum for the considered collection of *values*	$min(values) = \min_{i=\overline{1,n}} (values_i)$, where $n = \lvert values \rvert$
mode	Returns the mode for the considered collection of *values*	$mode(values)$ = element which appears most often in the list of *values*
product	Returns the product for the considered collection of *values*	$product(values) = \prod_{i=1}^{n} (values_i)$, where $n = \lvert values \rvert$
skew	Returns the skewness for the considered collection of *values*	$skew(values) = \frac{n}{(n-1)(n-2)} \sum_{i=1}^{n} \left(\frac{(values_i) - avg(values)}{stdev(values)} \right)^3$, where $n = \lvert values \rvert$
stdev	Returns the standard deviation for the considered collection of *values*	$stdev(values) = \sqrt{\frac{\sum_{i=1}^{n} ((values_i) - avg(values))^2}{(n-1)}}$, where $n = \lvert values \rvert$
sum	Returns the sum for the considered collection of *values*	$sum(values) = \sum_{i=1}^{n} (values_i)$, where $n = \lvert values \rvert$
var	Returns the variance for the considered collection of *values*	$var(values) = \frac{\sum_{i=1}^{n} ((values_i) - avg(values))^2}{(n-1)}$, where $n = \lvert values \rvert$

Table 5: Name, description and semantics of binary statistical measures

**Name**	**Description**	**Semantics**
covar	Returns the covariance for the considered collections of values $values_1$ and $values_2$	$covar(values_1, values_2) = \frac{1}{n-1} \sum_{i=1}^{n} (((values_{1_i})$ - $avg(values_1))$ $((values_{2_i})$ - $avg(values_2)))$, where $n = \min(\|values_1\|, \|values_2\|)$

Table 6: Name, description and semantics of binary statistical quantile measures

**Name**	**Description**	**Semantics**
percentile	Returns the pc-th $(0 \leq pc \leq 100)$ percentile for the considered collection of $values$	$percentile(values, pc) = i$-th value in the ordered list of $values$, where $i = \lfloor \frac{pc}{100} \times n + \frac{1}{2} \rfloor$, and $n = \|values\|$
quartile	Returns the i-th $(i \in \{25, 50, 75\})$ quartile for the considered collection of $values$	Let v be the ordered list of $values$, $m = median(values)$, L the sublist of values in v smaller than m, and U the sublist of values in v greater than m. $quartile(values) = median(L)$ if $i = 25$, m if $i = 50$, and $median(U)$ if $i = 75$.
