# Supplementary material for: A Novel Method to Verify Multilevel Computational Models of Biological Systems Using Multiscale Spatio-Temporal Meta Model Checking
Source: PLoS One. 2016 May 17;11(5):e0154847. doi: 10.1371/journal.pone.0154847 (PMC4871515; doi:10.1371/journal.pone.0154847)
Supplement: S5 Text — (PDF) [file pone.0154847.s005.pdf]

Proof that the multiscale spatio-temporal model checking problem is well-defined

To show that the multiscale spatio-temporal model checking problem is *well-defined* we will first prove that the number of required model simulations and state transitions within each simulation are finite.

Finite number of required simulations

Considering the approximate probabilistic model checking approaches described in Table 1, a finite number of model simulations is sufficient to determine if a logic property holds.

Table 1: Classification of considered approximate probabilistic model checking approaches. Bayesian methods consider prior knowledge when deciding if a logic property holds. Conversely frequentist approaches assume no prior knowledge is available. All methods except probabilistic black-box take as input a user-defined upper bound on the approximation error. They request additional model executions until the result is sufficiently accurate. Probabilistic black-box model checking takes a fixed number of model simulations as input and computes a p-value as the confidence level of the result.

	**Frequentist**	**Bayesian**
**Estimate**	Chernoff-Hoeffding bounds based [1]	Bayesian mean and variance based [5]
**Hypothesis testing**	Improved frequentist statistical hypothesis testing [4, 8]<br>Probabilistic black-box [6, 7]	Bayesian statistical hypothesis testing [2, 3]

Probabilistic black-box model checking is the only considered approach which can provide an answer regardless of the number of model simulations executed. The other considered model checking approaches require at least a certain number of model simulation traces to be available. Depending on the considered approach the minimum number of required model simulations can be determined at the beginning of the model verification process (for the Chernoff-Hoeffding bounds based approach) or not (for the improved frequentist and Bayesian statistical hypothesis testing, and Bayesian mean and variance estimate based approaches). Although all model checking methods require a finite number of model simulations, the execution time varies with the temporal bounds employed (see Definition 1 below), the value of the user-defined probability θ (for the probabilistic black-box, improved frequentist and Bayesian statistical hypothesis testing approaches), and the true probability p of the considered PBLMSTL specification to hold.

For practical applications users could potentially want to set an upper bound on the execution time before an answer is provided. Therefore the wrapper Algorithm 1 is employed to execute each model checking algorithm specified

in Table 1. If an answer can be provided using the requested approach within the specified execution time interval then it is reported to the user. Otherwise probabilistic black-box model checking is employed to report the answer based on the model simulations generated and evaluated so far.

In the beginning of Algorithm 1 `areMoreModelSimulationsRequired`, the boolean flag indicating if more model simulations are required, is set to true. Afterwards the collection of valid model `simulations` is initialised based on the given `simulationsInputSet`. The model checker of type `modelCheckingType` is then executed to verify if the logic property `logicProperty` holds considering the available `simulations` and set of `modelCheckingParameters`. While the number of elapsed minutes is less than `extraEvaluationTime` and the number of available model simulations is insufficient to evaluate `logicProperty` (i.e. `areMoreModelSimulationsRequired` is true) the loop comprising the following steps is executed:

1. Run `extraEvaluationProgram` to generate new simulations;
2. The collection of `simulations` is updated considering valid and previously unevaluated simulation input files;
3. The `modelCheckingType` model checker execution is resumed considering the additional `simulations`.

The loop is exited when either `extraEvaluationTime` minutes elapsed or enough model simulations have been provided (i.e. `areMoreModelSimulationsRequired` is false). In the former case the probabilistic black-box model checker is executed to provide a `result` considering the provided `multiscaleArchitectureGraph`. Otherwise the `result` is computed using the `modelCheckingType` model checker. In the end both `result` and `confidence` level are reported to the user; see Table 2 for a description of the input parameters, `result` and `confidence` level for each approximate probabilistic model checking approach considered. The `result` is encoded as a boolean value (i.e. true/false). Conversely the `confidence` level is encoded as one or more real values. For all model checking approaches considered the `confidence` level is equal to the input parameters, except probabilistic black-box where the `confidence` level represents p-values.

The main advantages of Algorithm 1 are:

- The model checking execution time and number of generated and evaluated simulations is finite. Depending on the parameters of the model checker, the distribution of the data and the number of required simulations, the answer will be provided using the desired model checker type or the default probabilistic black-box model checker.
- In contrast to traditional model checking methods in our approach the model checking task is decoupled from a specific model and model simulation environment. The path to an external program responsible for simulating the model and generating corresponding MSTML files is provided as input to the model checker. Whenever additional model simulations are

Algorithm 1 The wrapper algorithm employed to call specific model checking algorithms (see Table 1 for the considered approaches). If sufficient model simulations are generated and evaluated within *extraEvaluationTime* minutes, then the model checking algorithm selected by the user is employed to provide an answer. Otherwise the user is informed that the maximum evaluation time threshold was reached and the answer is provided using the probabilistic black-box model checking approach. Model simulations are generated and stored in an input set *simulationsInputSet* using the external model simulation program *extraEvaluationProgram*. The logic property to be verified is stored in the variable *logicProperty*.

Require: *modelCheckingType* is the specific model checking approach, *modelCheckingParameters* is the collection of parameters required by the chosen *modelCheckingType*, *extraEvaluationTime* is the maximum number of minutes allowed for generating and evaluating additional model simulations, *extraEvaluationProgram* is the model simulation program which is called whenever new simulations are required, *simulationsInputSet* is the set containing the simulations, and *logicProperty* is the PBLMSTL logic property to be verified

Ensure: A true/false answer together with a confidence level is provided

```
areMoreModelSimulationsRequired ← true;
simulations ← GetSimulations(simulationsInputSet);
RunModelChecker(modelCheckingType, modelCheckingParameters,
                simulations, logicProperty,
                multiscaleArchitectureGraph, result,
                confidence, areMoreModelSimulationsRequired);
while (number of minutes elapsed < extraEvaluationTime) AND
      (areMoreModelSimulationsRequired == true) do
    GenerateModelSimulations(extraEvaluationProgram);
    UpdateCollectionOfSimulations(simulations, simulationsInputSet);
    RunModelChecker(modelCheckingType, modelCheckingParameters,
                    simulations, logicProperty,
                    multiscaleArchitectureGraph, result,
                    confidence, areMoreModelSimulationsRequired);
end while
if (areMoreModelSimulationsRequired == true) then
    RunProbBlackBoxModelChecker(simulations, logicProperty,
                                multiscaleArchitectureGraph,
                                result, confidence);
end if
Output result and confidence;
```

required this external program is executed. For the algorithm implemen-

Table 2: Mapping between input parameters, `result` and `confidence` level for the considered approximate probabilistic model checking approaches. Columns from left to right record the name, input parameters (excluding ϕ and MSTML files), result and confidence level corresponding to each approximate probabilistic model checking approach, where $\phi \equiv P_{\bowtie\theta}[\psi]$ denotes the PBLM-STL statement against which the model is verified. The description of the input parameters was previously given in Table 2 in the main paper and will not be restated here.

**Name**	**Input**	**`result`**	**`confidence` level**
Chernoff-Hoeffding bounds based	ϵ, δ	True if $p \bowtie \theta$, where p is the estimated probability of ψ to hold, and false otherwise.	ϵ, δ
Improved frequentist statistical hypothesis testing	α, β	True, if we fail to reject the null hypothesis (corresponding to ϕ), and false otherwise.	α, β
Probabilistic black-box	-	True, if the p-value computed for the null hypothesis (corresponding to ϕ) is smaller than the p-value corresponding to the alternative hypothesis (corresponding to the opposite of ϕ), and false otherwise.	p-values computed for the null and the alternative hypotheses
Bayesian mean and variance based	α, β, T	True if $p \bowtie \theta$, where p is the estimated probability of ψ to hold, and false otherwise.	α, β, T
Bayesian statistical hypothesis testing	α, β, T	True, if we fail to reject the null hypothesis (corresponding to ϕ), and false otherwise.	α, β, T

tation our recommendation is that the employed external program could be a script (e.g. Bash [UNIX], Batch [Windows]) which calls the model simulator, processes the output and stores the resulting MSTML files into a predefined location.

Finite number of state transitions

Bounded temporal logic (including BLMSTL) properties can be evaluated against model simulations which cover only a finite interval of time. The upper bound of this interval can be computed based on the temporal operators/functions contained by the evaluated logic properties. Let us denote the upper bound corresponding to a generic BLMSTL logic property ψ by $\lceil\psi\rceil$.

Definition 1. *The* upper bound $\lceil\psi\rceil \in \mathbb{R}_+$ *corresponding to a BLMSTL logic property* ψ *considering an execution* σ *and the abbreviations introduced in S5 Text, Table 1 is defined recursively on the structure of the logic property as follows:*

- $\lceil tnm_1 \asymp tnm_2\rceil = \max(\lceil tnm_1\rceil, \lceil tnm_2\rceil)$;
- $\lceil cm(tnm_1) \asymp tnm_2\rceil = \max(1 + \lceil tnm_1\rceil, \lceil tnm_2\rceil) \leq 1 + \max(\lceil tnm_1\rceil, \lceil tnm_2\rceil)$ *because the value of* tnm_1 *is computed considering both* σ^0 *and* σ^1;
- $\lceil\sim\psi\rceil = \lceil\psi\rceil$;
- $\lceil\psi_1 \wedge \psi_2\rceil = \max(\lceil\psi_1\rceil, \lceil\psi_2\rceil)$;
- $\lceil\psi_1 \vee \psi_2\rceil = \max(\lceil\psi_1\rceil, \lceil\psi_2\rceil)$;
- $\lceil\psi_1 \Rightarrow \psi_2\rceil = \max(\lceil\psi_1\rceil, \lceil\psi_2\rceil)$;
- $\lceil\psi_1 \Leftrightarrow \psi_2\rceil = \max(\lceil\psi_1\rceil, \lceil\psi_2\rceil)$;
- $\lceil\psi_1\ U[a,b]\ \psi_2\rceil = b + \max(\lceil\psi_1\rceil, \lceil\psi_2\rceil)$;
- $\lceil F[a,b]\ \psi\rceil = b + \lceil\psi\rceil$;
- $\lceil G[a,b]\ \psi\rceil = b + \lceil\psi\rceil$;
- $\lceil X\ \psi\rceil = 1 + \lceil\psi\rceil$;
- $\lceil X[k]\ \psi\rceil = k + \lceil\psi\rceil$;
- $\lceil(\psi)\rceil = \lceil\psi\rceil$.
- *The upper bound* $\lceil tnm\rceil$ *corresponding to the temporal numeric measure* tnm *is defined recursively on the structure of the temporal numeric measure as follows:*
  - $\lceil re\rceil = 0$, *because the value of* re *is employed directly;*
  - $\lceil nsv\rceil = 0$, *because the value of* nsv *is computed considering only* $\sigma[0]$;
  - $\lceil nstm\rceil$ *which is computed as described below;*
  - $\lceil unm(tnm)\rceil = \lceil tnm\rceil$;
  - $\lceil bnm(tnm_1, tnm_2)\rceil = \max(\lceil tnm_1\rceil, \lceil tnm_2\rceil)$.
- *The upper bound* $\lceil nstm\rceil$ *corresponding to the numeric statistical measure* $nstm$ *is defined recursively on the structure of the numeric statistical measure as follows:*

– $\lceil usm(nmc) \rceil = \lceil nmc \rceil$;
– $\lceil bsm(nmc_1, nmc_2) \rceil = \max(\lceil nmc_1 \rceil, \lceil nmc_2 \rceil)$;
– $\lceil bsqm(nmc, re) \rceil = \lceil nmc \rceil$.

- *The upper bound $\lceil nmc \rceil$ corresponding to the numeric measure collection nmc is defined recursively on the structure of the numeric measure collection as follows:*
  – $\lceil [a,b]nm \rceil = b + \lceil nm \rceil$;
  – $\lceil smc \rceil = 0$, *because the value of smc is computed considering only* $\sigma[0]$.
- *The upper bound $\lceil nm \rceil$ corresponding to the numeric measure nm is defined recursively on the structure of the numeric measure as follows:*
  – $\lceil pnm \rceil = 0$, *because the value of pnm is computed considering only* $\sigma[0]$;
  – $\lceil unm(nm) \rceil = \lceil nm \rceil$;
  – $\lceil bnm(nm_1, nm_2) \rceil = \max(\lceil nm_1 \rceil, \lceil nm_2 \rceil)$.

Thus the minimum upper bound for the simulation time interval to be covered by model executions when verifying a BLMSTL logic property ψ is $\lceil \psi \rceil$.

Lemma 1. *Let us assume that a BLMSTL logic property ψ is verified against an infinite execution $\sigma = \{(s_0, t_0), (s_1, t_1), (s_2, t_2), ...\}$. Moreover let us denote a finite prefix of σ by $\hat{\sigma} = \{(\hat{s_0}, \hat{t_0}), (\hat{s_1}, \hat{t_1}), ..., (\hat{s_m}, \hat{t_m})\}$, where*

$$\hat{s_i} = s_i \text{ and } \hat{t_i} = t_i, \forall i = \overline{0,m} \text{ with } \sum_{i=0}^{m} t_i \geq \lceil \psi \rceil \text{ and } \sum_{i=0}^{m-1} t_i < \lceil \psi \rceil.$$

Then $\sigma \models \psi$ **if and only if** *$\hat{\sigma} \models \psi$.*

Proof. Trivial. □

Lemma 2. *The number of state transitions required to verify if a BLMSTL logic property holds is* finite.

Proof. Trivial. □

Well-defined model checking problem

Theorem 1. *The multiscale spatio-temporal model checking problem is* well-defined.

Proof. In Subsection "Finite number of required simulations" it was shown that the number of model simulations required to verify if a PBLMSTL logic property ϕ holds is finite. Moreover according to Lemmas 1 and 2 only a finite prefix and a finite number of state transitions has to be considered for each model simulation. Thus the evaluation of ϕ is reduced to the problem of evaluating atomic properties over a finite number of states for each model simulation which is decidable. Hence the model checking problem is well-defined. □

References

[1] Thomas Hérault, Richard Lassaigne, Frédéric Magniette, and Sylvain Peyronnet. Approximate probabilistic model checking. In Bernhard Steffen and Giorgio Levi, editors, *Verification, Model Checking, and Abstract Interpretation*, number 2937 in Lecture Notes in Computer Science, pages 73–84. Springer Berlin Heidelberg, January 2004.

[2] Sumit K. Jha, Edmund M. Clarke, Christopher J. Langmead, Axel Legay, André Platzer, and Paolo Zuliani. A bayesian approach to model checking biological systems. In Pierpaolo Degano and Roberto Gorrieri, editors, *Computational Methods in Systems Biology*, number 5688 in Lecture Notes in Computer Science, pages 218–234. Springer Berlin Heidelberg, January 2009.

[3] Sumit Kumar Jha, Edmund M Clarke, Christopher J Langmead, Axel Legay, Andre Platzer, and Paolo Zuliani. Statistical model checking for complex stochastic models in systems biology. Technical report, Carnegie Mellon University, 2009.

[4] Chuan Hock Koh, Sucheendra K. Palaniappan, P. S. Thiagarajan, and Limsoon Wong. Improved statistical model checking methods for pathway analysis. *BMC Bioinformatics*, 13(Suppl 17):S15, December 2012. PMID: 23282174.

[5] C.J. Langmead. Generalized Queries and Bayesian Statistical Model Checking in Dynamic Bayesian Networks: Application to Personalized Medicine. In *Proc. of the 8th International Conference on Computational Systems Bioinformatics (CSB)*, pages 201–212, California, August 2009. Life Sciences Society.

[6] Koushik Sen, Mahesh Viswanathan, and Gul Agha. Statistical model checking of black-box probabilistic systems. In Rajeev Alur and Doron A. Peled, editors, *Computer Aided Verification*, number 3114 in Lecture Notes in Computer Science, pages 202–215. Springer Berlin Heidelberg, January 2004.

[7] Håkan L. S. Younes. Probabilistic verification for "Black-Box" systems. In Kousha Etessami and Sriram K. Rajamani, editors, *Computer Aided Verification*, number 3576 in Lecture Notes in Computer Science, pages 253–265. Springer Berlin Heidelberg, January 2005.

[8] Håkan L.S. Younes and Reid G. Simmons. Statistical probabilistic model checking with a focus on time-bounded properties. *Information and Computation*, 204(9):1368–1409, September 2006.
