# Supplementary material for: A Novel Method to Verify Multilevel Computational Models of Biological Systems Using Multiscale Spatio-Temporal Meta Model Checking
Source: PLoS One. 2016 May 17;11(5):e0154847. doi: 10.1371/journal.pone.0154847 (PMC4871515; doi:10.1371/journal.pone.0154847)
Supplement: S7 Text — (PDF) [file pone.0154847.s007.pdf]

Model checking results for the uterine contractions of labour case study

For the convenience of the reader the set of PBLMSTL statements considered for the uterine contractions of labour case study will be restated below:

$$P > 0.9\ [G\ [1, 329]\ (((d(\{Pressure\}(scaleAndSubsystem = Organ.Uterus)) > 0)\ \wedge ((d(sum(density(filter(regions, scaleAndSubsystem = Tissue.ContractileActivity)))) > 0))) \vee ((d(\{Pressure\}(scaleAndSubsystem = Organ.Uterus)) < 0)\ \wedge ((d(sum(density(filter(regions, scaleAndSubsystem = Tissue.ContractileActivity)))) < 0))) \vee (d(\{Pressure\}(scaleAndSubsystem = Organ.Uterus)) = 0))] \tag{4}$$

$$P < 0.1\ [F\ [1, 329]\ ((d(\{Pressure\}(scaleAndSubsystem = Organ.Uterus)) < 0) \wedge (min(area(filter(regions, scaleAndSubsystem = Tissue.BurstActivity))) = 16))] \tag{5}$$

$$P > 0.9\ [G\ [1, 329]\ (((max(area(filter(regions, scaleAndSubsystem = Tissue.RefractoryActivity))) = 0))\ \Rightarrow ((d(\{Pressure\}(scaleAndSubsystem = Organ.Uterus)) < 0)\ \vee ((d(\{Pressure\}(scaleAndSubsystem = Organ.Uterus)) = 0) \wedge (\{Pressure\}(scaleAndSubsystem = Organ.Uterus) = 0.5))))] \tag{6}$$

Each table describes the results corresponding to one of the PBLMSTL statements. The first column of each row represents the identifier of the model checking execution. The second column represents the evaluation result (T = true, F = false) of the PBLMSTL statement for that particular model checker execution. The number of MSTML files against which the PBLMSTL statement was executed, respectively how many of them evaluated true and how many evaluated false is provided in columns three, four and five. Finally column six presents the execution time (minutes:seconds format) corresponding to each model checker run. All executions of the model checker employed the probabilistic black-box model checking approach.

Table 1: Model checking results corresponding to PBLMSTL statement 4

Id	Result	#total	#true	#false	Execution time (min:sec)
1	TRUE	1	1	0	0:00.56
2	TRUE	1	1	0	0:00.54
3	TRUE	1	1	0	0:00.54
4	TRUE	1	1	0	0:00.54
5	TRUE	1	1	0	0:00.55
6	TRUE	1	1	0	0:00.54
7	TRUE	1	1	0	0:00.54
8	TRUE	1	1	0	0:00.54
9	TRUE	1	1	0	0:00.54
10	TRUE	1	1	0	0:00.54
11	TRUE	1	1	0	0:00.54
12	TRUE	1	1	0	0:00.54
13	TRUE	1	1	0	0:00.54
14	TRUE	1	1	0	0:00.54
15	TRUE	1	1	0	0:00.54
16	TRUE	1	1	0	0:00.54
17	TRUE	1	1	0	0:00.54
18	TRUE	1	1	0	0:00.54
19	TRUE	1	1	0	0:00.54
20	TRUE	1	1	0	0:00.55
21	TRUE	1	1	0	0:00.54
22	TRUE	1	1	0	0:00.54
23	TRUE	1	1	0	0:00.54
24	TRUE	1	1	0	0:00.55
25	TRUE	1	1	0	0:00.54
26	TRUE	1	1	0	0:00.54
27	TRUE	1	1	0	0:00.55
28	TRUE	1	1	0	0:00.54
29	TRUE	1	1	0	0:00.54
30	TRUE	1	1	0	0:00.54
31	TRUE	1	1	0	0:00.54
32	TRUE	1	1	0	0:00.53
33	TRUE	1	1	0	0:00.54
34	TRUE	1	1	0	0:00.54
35	TRUE	1	1	0	0:00.54
36	TRUE	1	1	0	0:00.54
37	TRUE	1	1	0	0:00.54
38	TRUE	1	1	0	0:00.54
39	TRUE	1	1	0	0:00.54
40	TRUE	1	1	0	0:00.54
41	TRUE	1	1	0	0:00.54
42	TRUE	1	1	0	0:00.55
43	TRUE	1	1	0	0:00.55
44	TRUE	1	1	0	0:00.54
45	TRUE	1	1	0	0:00.54
46	TRUE	1	1	0	0:00.54

Id	Result	#total	#true	#false	Execution time (min:sec)
47	TRUE	1	1	0	0:00.53
48	TRUE	1	1	0	0:00.55
49	TRUE	1	1	0	0:00.54
50	TRUE	1	1	0	0:00.54
51	TRUE	1	1	0	0:00.54
52	TRUE	1	1	0	0:00.54
53	TRUE	1	1	0	0:00.54
54	TRUE	1	1	0	0:00.55
55	TRUE	1	1	0	0:00.54
56	TRUE	1	1	0	0:00.54
57	TRUE	1	1	0	0:00.54
58	TRUE	1	1	0	0:00.54
59	TRUE	1	1	0	0:00.54
60	TRUE	1	1	0	0:00.53
61	TRUE	1	1	0	0:00.54
62	TRUE	1	1	0	0:00.53
63	TRUE	1	1	0	0:00.56
64	TRUE	1	1	0	0:00.54
65	TRUE	1	1	0	0:00.54
66	TRUE	1	1	0	0:00.54
67	TRUE	1	1	0	0:00.54
68	TRUE	1	1	0	0:00.56
69	TRUE	1	1	0	0:00.52
70	TRUE	1	1	0	0:00.54
71	TRUE	1	1	0	0:00.55
72	TRUE	1	1	0	0:00.54
73	TRUE	1	1	0	0:00.53
74	TRUE	1	1	0	0:00.54
75	TRUE	1	1	0	0:00.54
76	TRUE	1	1	0	0:00.54
77	TRUE	1	1	0	0:00.54
78	TRUE	1	1	0	0:00.53
79	TRUE	1	1	0	0:00.54
80	TRUE	1	1	0	0:00.53
81	TRUE	1	1	0	0:00.53
82	TRUE	1	1	0	0:00.54
83	TRUE	1	1	0	0:00.54
84	TRUE	1	1	0	0:00.55
85	TRUE	1	1	0	0:00.54
86	TRUE	1	1	0	0:00.54
87	TRUE	1	1	0	0:00.54
88	TRUE	1	1	0	0:00.55
89	TRUE	1	1	0	0:00.53
90	TRUE	1	1	0	0:00.54
91	TRUE	1	1	0	0:00.54
92	TRUE	1	1	0	0:00.54
93	TRUE	1	1	0	0:00.53
94	TRUE	1	1	0	0:00.55

Id	Result	#total	#true	#false	Execution time (min:sec)
95	TRUE	1	1	0	0:00.53
96	TRUE	1	1	0	0:00.54
97	TRUE	1	1	0	0:00.54
98	TRUE	1	1	0	0:00.54
99	TRUE	1	1	0	0:00.52
100	TRUE	1	1	0	0:00.54
101	TRUE	1	1	0	0:00.55
102	TRUE	1	1	0	0:00.54
103	TRUE	1	1	0	0:00.53
104	TRUE	1	1	0	0:00.55
105	TRUE	1	1	0	0:00.54
106	TRUE	1	1	0	0:00.55
107	TRUE	1	1	0	0:00.54
108	TRUE	1	1	0	0:00.53
109	TRUE	1	1	0	0:00.53
110	TRUE	1	1	0	0:00.53
111	TRUE	1	1	0	0:00.54
112	TRUE	1	1	0	0:00.54
113	TRUE	1	1	0	0:00.54
114	TRUE	1	1	0	0:00.54
115	TRUE	1	1	0	0:00.54
116	TRUE	1	1	0	0:00.54
117	TRUE	1	1	0	0:00.55
118	TRUE	1	1	0	0:00.54
119	TRUE	1	1	0	0:00.54
120	TRUE	1	1	0	0:00.54
121	TRUE	1	1	0	0:00.54
122	TRUE	1	1	0	0:00.53
123	TRUE	1	1	0	0:00.54
124	TRUE	1	1	0	0:00.53
125	TRUE	1	1	0	0:00.54
126	TRUE	1	1	0	0:00.54
127	TRUE	1	1	0	0:00.54
128	TRUE	1	1	0	0:00.55
129	TRUE	1	1	0	0:00.54
130	TRUE	1	1	0	0:00.55
131	TRUE	1	1	0	0:00.55
132	TRUE	1	1	0	0:00.54
133	TRUE	1	1	0	0:00.54
134	TRUE	1	1	0	0:00.54
135	TRUE	1	1	0	0:00.53
136	TRUE	1	1	0	0:00.54
137	TRUE	1	1	0	0:00.54
138	TRUE	1	1	0	0:00.56
139	TRUE	1	1	0	0:00.54
140	TRUE	1	1	0	0:00.54
141	TRUE	1	1	0	0:00.53
142	TRUE	1	1	0	0:00.54

Id	Result	#total	#true	#false	Execution time (min:sec)
143	TRUE	1	1	0	0:00.54
144	TRUE	1	1	0	0:00.55
145	TRUE	1	1	0	0:00.54
146	TRUE	1	1	0	0:00.54
147	TRUE	1	1	0	0:00.54
148	TRUE	1	1	0	0:00.54
149	TRUE	1	1	0	0:00.54
150	TRUE	1	1	0	0:00.54
151	TRUE	1	1	0	0:00.54
152	TRUE	1	1	0	0:00.55
153	TRUE	1	1	0	0:00.54
154	TRUE	1	1	0	0:00.54
155	TRUE	1	1	0	0:00.54
156	TRUE	1	1	0	0:00.54
157	TRUE	1	1	0	0:00.54
158	TRUE	1	1	0	0:00.54
159	TRUE	1	1	0	0:00.54
160	TRUE	1	1	0	0:00.55
161	TRUE	1	1	0	0:00.55
162	TRUE	1	1	0	0:00.53
163	TRUE	1	1	0	0:00.53
164	TRUE	1	1	0	0:00.54
165	TRUE	1	1	0	0:00.54
166	TRUE	1	1	0	0:00.54
167	TRUE	1	1	0	0:00.54
168	TRUE	1	1	0	0:00.54
169	TRUE	1	1	0	0:00.54
170	TRUE	1	1	0	0:00.55
171	TRUE	1	1	0	0:00.54
172	TRUE	1	1	0	0:00.54
173	TRUE	1	1	0	0:00.54
174	TRUE	1	1	0	0:00.54
175	TRUE	1	1	0	0:00.54
176	TRUE	1	1	0	0:00.54
177	TRUE	1	1	0	0:00.54
178	TRUE	1	1	0	0:00.55
179	TRUE	1	1	0	0:00.56
180	TRUE	1	1	0	0:00.54
181	TRUE	1	1	0	0:00.55
182	TRUE	1	1	0	0:00.54
183	TRUE	1	1	0	0:00.55
184	TRUE	1	1	0	0:00.54
185	TRUE	1	1	0	0:00.54
186	TRUE	1	1	0	0:00.53
187	TRUE	1	1	0	0:00.55
188	TRUE	1	1	0	0:00.53
189	TRUE	1	1	0	0:00.54
190	TRUE	1	1	0	0:00.54

Id	Result	#total	#true	#false	Execution time (min:sec)
191	TRUE	1	1	0	0:00.54
192	TRUE	1	1	0	0:00.54
193	TRUE	1	1	0	0:00.54
194	TRUE	1	1	0	0:00.54
195	TRUE	1	1	0	0:00.55
196	TRUE	1	1	0	0:00.54
197	TRUE	1	1	0	0:00.52
198	TRUE	1	1	0	0:00.54
199	TRUE	1	1	0	0:00.54
200	TRUE	1	1	0	0:00.54
201	TRUE	1	1	0	0:00.54
202	TRUE	1	1	0	0:00.53
203	TRUE	1	1	0	0:00.54
204	TRUE	1	1	0	0:00.53
205	TRUE	1	1	0	0:00.54
206	TRUE	1	1	0	0:00.54
207	TRUE	1	1	0	0:00.54
208	TRUE	1	1	0	0:00.54
209	TRUE	1	1	0	0:00.53
210	TRUE	1	1	0	0:00.54
211	TRUE	1	1	0	0:00.53
212	TRUE	1	1	0	0:00.54
213	TRUE	1	1	0	0:00.54
214	TRUE	1	1	0	0:00.53
215	TRUE	1	1	0	0:00.54
216	TRUE	1	1	0	0:00.54
217	TRUE	1	1	0	0:00.53
218	TRUE	1	1	0	0:00.54
219	TRUE	1	1	0	0:00.54
220	TRUE	1	1	0	0:00.54
221	TRUE	1	1	0	0:00.54
222	TRUE	1	1	0	0:00.55
223	TRUE	1	1	0	0:00.55
224	TRUE	1	1	0	0:00.54
225	TRUE	1	1	0	0:00.54
226	TRUE	1	1	0	0:00.54
227	TRUE	1	1	0	0:00.55
228	TRUE	1	1	0	0:00.54
229	TRUE	1	1	0	0:00.55
230	TRUE	1	1	0	0:00.54
231	TRUE	1	1	0	0:00.54
232	TRUE	1	1	0	0:00.53
233	TRUE	1	1	0	0:00.54
234	TRUE	1	1	0	0:00.54
235	TRUE	1	1	0	0:00.54
236	TRUE	1	1	0	0:00.54
237	TRUE	1	1	0	0:00.54
238	TRUE	1	1	0	0:00.53

Id	Result	#total	#true	#false	Execution time (min:sec)
239	TRUE	1	1	0	0:00.54
240	TRUE	1	1	0	0:00.54
241	TRUE	1	1	0	0:00.54
242	TRUE	1	1	0	0:00.54
243	TRUE	1	1	0	0:00.53
244	TRUE	1	1	0	0:00.54
245	TRUE	1	1	0	0:00.54
246	TRUE	1	1	0	0:00.54
247	TRUE	1	1	0	0:00.54
248	TRUE	1	1	0	0:00.54
249	TRUE	1	1	0	0:00.54
250	TRUE	1	1	0	0:00.54
251	TRUE	1	1	0	0:00.54
252	TRUE	1	1	0	0:00.54
253	TRUE	1	1	0	0:00.54
254	TRUE	1	1	0	0:00.54
255	TRUE	1	1	0	0:00.54
256	TRUE	1	1	0	0:00.53
257	TRUE	1	1	0	0:00.54
258	TRUE	1	1	0	0:00.53
259	TRUE	1	1	0	0:00.54
260	TRUE	1	1	0	0:00.55
261	TRUE	1	1	0	0:00.55
262	TRUE	1	1	0	0:00.54
263	TRUE	1	1	0	0:00.55
264	TRUE	1	1	0	0:00.55
265	TRUE	1	1	0	0:00.54
266	TRUE	1	1	0	0:00.55
267	TRUE	1	1	0	0:00.54
268	TRUE	1	1	0	0:00.52
269	TRUE	1	1	0	0:00.54
270	TRUE	1	1	0	0:00.55
271	TRUE	1	1	0	0:00.54
272	TRUE	1	1	0	0:00.54
273	TRUE	1	1	0	0:00.55
274	TRUE	1	1	0	0:00.54
275	TRUE	1	1	0	0:00.55
276	TRUE	1	1	0	0:00.53
277	TRUE	1	1	0	0:00.54
278	TRUE	1	1	0	0:00.55
279	TRUE	1	1	0	0:00.54
280	TRUE	1	1	0	0:00.54
281	TRUE	1	1	0	0:00.54
282	TRUE	1	1	0	0:00.54
283	TRUE	1	1	0	0:00.53
284	TRUE	1	1	0	0:00.54
285	TRUE	1	1	0	0:00.54
286	TRUE	1	1	0	0:00.52

Id	Result	#total	#true	#false	Execution time (min:sec)
287	TRUE	1	1	0	0:00.54
288	TRUE	1	1	0	0:00.54
289	TRUE	1	1	0	0:00.53
290	TRUE	1	1	0	0:00.54
291	TRUE	1	1	0	0:00.54
292	TRUE	1	1	0	0:00.54
293	TRUE	1	1	0	0:00.54
294	TRUE	1	1	0	0:00.54
295	TRUE	1	1	0	0:00.54
296	TRUE	1	1	0	0:00.54
297	TRUE	1	1	0	0:00.54
298	TRUE	1	1	0	0:00.54
299	TRUE	1	1	0	0:00.54
300	TRUE	1	1	0	0:00.54
301	TRUE	1	1	0	0:00.54
302	TRUE	1	1	0	0:00.54
303	TRUE	1	1	0	0:00.54
304	TRUE	1	1	0	0:00.54
305	TRUE	1	1	0	0:00.54
306	TRUE	1	1	0	0:00.54
307	TRUE	1	1	0	0:00.54
308	TRUE	1	1	0	0:00.54
309	TRUE	1	1	0	0:00.54
310	TRUE	1	1	0	0:00.54
311	TRUE	1	1	0	0:00.55
312	TRUE	1	1	0	0:00.54
313	TRUE	1	1	0	0:00.55
314	TRUE	1	1	0	0:00.54
315	TRUE	1	1	0	0:00.54
316	TRUE	1	1	0	0:00.55
317	TRUE	1	1	0	0:00.55
318	TRUE	1	1	0	0:00.54
319	TRUE	1	1	0	0:00.53
320	TRUE	1	1	0	0:00.54
321	TRUE	1	1	0	0:00.54
322	TRUE	1	1	0	0:00.53
323	TRUE	1	1	0	0:00.55
324	TRUE	1	1	0	0:00.54
325	TRUE	1	1	0	0:00.54
326	TRUE	1	1	0	0:00.54
327	TRUE	1	1	0	0:00.54
328	TRUE	1	1	0	0:00.54
329	TRUE	1	1	0	0:00.55
330	TRUE	1	1	0	0:00.55
331	TRUE	1	1	0	0:00.54
332	TRUE	1	1	0	0:00.54
333	TRUE	1	1	0	0:00.53
334	TRUE	1	1	0	0:00.55

Id	Result	#total	#true	#false	Execution time (min:sec)
335	TRUE	1	1	0	0:00.52
336	TRUE	1	1	0	0:00.54
337	TRUE	1	1	0	0:00.53
338	TRUE	1	1	0	0:00.54
339	TRUE	1	1	0	0:00.55
340	TRUE	1	1	0	0:00.52
341	TRUE	1	1	0	0:00.55
342	TRUE	1	1	0	0:00.55
343	TRUE	1	1	0	0:00.55
344	TRUE	1	1	0	0:00.54
345	TRUE	1	1	0	0:00.54
346	TRUE	1	1	0	0:00.53
347	TRUE	1	1	0	0:00.54
348	TRUE	1	1	0	0:00.54
349	TRUE	1	1	0	0:00.55
350	TRUE	1	1	0	0:00.55
351	TRUE	1	1	0	0:00.54
352	TRUE	1	1	0	0:00.54
353	TRUE	1	1	0	0:00.55
354	TRUE	1	1	0	0:00.54
355	TRUE	1	1	0	0:00.54
356	TRUE	1	1	0	0:00.53
357	TRUE	1	1	0	0:00.54
358	TRUE	1	1	0	0:00.53
359	TRUE	1	1	0	0:00.55
360	TRUE	1	1	0	0:00.54
361	TRUE	1	1	0	0:00.54
362	TRUE	1	1	0	0:00.53
363	TRUE	1	1	0	0:00.55
364	TRUE	1	1	0	0:00.54
365	TRUE	1	1	0	0:00.55
366	TRUE	1	1	0	0:00.54
367	TRUE	1	1	0	0:00.53
368	TRUE	1	1	0	0:00.55
369	TRUE	1	1	0	0:00.54
370	TRUE	1	1	0	0:00.54
371	TRUE	1	1	0	0:00.54
372	TRUE	1	1	0	0:00.55
373	TRUE	1	1	0	0:00.54
374	TRUE	1	1	0	0:00.55
375	TRUE	1	1	0	0:00.53
376	TRUE	1	1	0	0:00.53
377	TRUE	1	1	0	0:00.54
378	TRUE	1	1	0	0:00.54
379	TRUE	1	1	0	0:00.54
380	TRUE	1	1	0	0:00.54
381	TRUE	1	1	0	0:00.54
382	TRUE	1	1	0	0:00.54

Id	Result	#total	#true	#false	Execution time (min:sec)
383	TRUE	1	1	0	0:00.55
384	TRUE	1	1	0	0:00.53
385	TRUE	1	1	0	0:00.53
386	TRUE	1	1	0	0:00.53
387	TRUE	1	1	0	0:00.53
388	TRUE	1	1	0	0:00.54
389	TRUE	1	1	0	0:00.54
390	TRUE	1	1	0	0:00.54
391	TRUE	1	1	0	0:00.54
392	TRUE	1	1	0	0:00.54
393	TRUE	1	1	0	0:00.54
394	TRUE	1	1	0	0:00.53
395	TRUE	1	1	0	0:00.53
396	TRUE	1	1	0	0:00.54
397	TRUE	1	1	0	0:00.55
398	TRUE	1	1	0	0:00.55
399	TRUE	1	1	0	0:00.54
400	TRUE	1	1	0	0:00.55
401	TRUE	1	1	0	0:00.55
402	TRUE	1	1	0	0:00.54
403	TRUE	1	1	0	0:00.53
404	TRUE	1	1	0	0:00.54
405	TRUE	1	1	0	0:00.57
406	TRUE	1	1	0	0:00.55
407	TRUE	1	1	0	0:00.53
408	TRUE	1	1	0	0:00.54
409	TRUE	1	1	0	0:00.54
410	TRUE	1	1	0	0:00.57
411	TRUE	1	1	0	0:00.54
412	TRUE	1	1	0	0:00.53
413	TRUE	1	1	0	0:00.54
414	TRUE	1	1	0	0:00.54
415	TRUE	1	1	0	0:00.54
416	TRUE	1	1	0	0:00.54
417	TRUE	1	1	0	0:00.53
418	TRUE	1	1	0	0:00.53
419	TRUE	1	1	0	0:00.54
420	TRUE	1	1	0	0:00.54
421	TRUE	1	1	0	0:00.53
422	TRUE	1	1	0	0:00.54
423	TRUE	1	1	0	0:00.53
424	TRUE	1	1	0	0:00.54
425	TRUE	1	1	0	0:00.54
426	TRUE	1	1	0	0:00.54
427	TRUE	1	1	0	0:00.52
428	TRUE	1	1	0	0:00.54
429	TRUE	1	1	0	0:00.54
430	TRUE	1	1	0	0:00.54

Id	Result	#total	#true	#false	Execution time (min:sec)
431	TRUE	1	1	0	0:00.53
432	TRUE	1	1	0	0:00.53
433	TRUE	1	1	0	0:00.54
434	TRUE	1	1	0	0:00.54
435	TRUE	1	1	0	0:00.54
436	TRUE	1	1	0	0:00.54
437	TRUE	1	1	0	0:00.55
438	TRUE	1	1	0	0:00.53
439	TRUE	1	1	0	0:00.54
440	TRUE	1	1	0	0:00.54
441	TRUE	1	1	0	0:00.54
442	TRUE	1	1	0	0:00.54
443	TRUE	1	1	0	0:00.54
444	TRUE	1	1	0	0:00.55
445	TRUE	1	1	0	0:00.53
446	TRUE	1	1	0	0:00.55
447	TRUE	1	1	0	0:00.54
448	TRUE	1	1	0	0:00.54
449	TRUE	1	1	0	0:00.56
450	TRUE	1	1	0	0:00.54
451	TRUE	1	1	0	0:00.54
452	TRUE	1	1	0	0:00.54
453	TRUE	1	1	0	0:00.54
454	TRUE	1	1	0	0:00.54
455	TRUE	1	1	0	0:00.52
456	TRUE	1	1	0	0:00.54
457	TRUE	1	1	0	0:00.54
458	TRUE	1	1	0	0:00.54
459	TRUE	1	1	0	0:00.54
460	TRUE	1	1	0	0:00.55
461	TRUE	1	1	0	0:00.54
462	TRUE	1	1	0	0:00.54
463	TRUE	1	1	0	0:00.54
464	TRUE	1	1	0	0:00.54
465	TRUE	1	1	0	0:00.55
466	TRUE	1	1	0	0:00.54
467	TRUE	1	1	0	0:00.54
468	TRUE	1	1	0	0:00.54
469	TRUE	1	1	0	0:00.54
470	TRUE	1	1	0	0:00.54
471	TRUE	1	1	0	0:00.54
472	TRUE	1	1	0	0:00.54
473	TRUE	1	1	0	0:00.53
474	TRUE	1	1	0	0:00.54
475	TRUE	1	1	0	0:00.54
476	TRUE	1	1	0	0:00.54
477	TRUE	1	1	0	0:00.54
478	TRUE	1	1	0	0:00.54

Id	Result	#total	#true	#false	Execution time (min:sec)
479	TRUE	1	1	0	0:00.54
480	TRUE	1	1	0	0:00.55
481	TRUE	1	1	0	0:00.54
482	TRUE	1	1	0	0:00.55
483	TRUE	1	1	0	0:00.53
484	TRUE	1	1	0	0:00.54
485	TRUE	1	1	0	0:00.54
486	TRUE	1	1	0	0:00.54
487	TRUE	1	1	0	0:00.54
488	TRUE	1	1	0	0:00.54
489	TRUE	1	1	0	0:00.54
490	TRUE	1	1	0	0:00.56
491	TRUE	1	1	0	0:00.54
492	TRUE	1	1	0	0:00.54
493	TRUE	1	1	0	0:00.54
494	TRUE	1	1	0	0:00.55
495	TRUE	1	1	0	0:00.54
496	TRUE	1	1	0	0:00.54
497	TRUE	1	1	0	0:00.54
498	TRUE	1	1	0	0:00.54
499	TRUE	1	1	0	0:00.55
500	TRUE	1	1	0	0:00.54

Table 2: Model checking results corresponding to PBLMSTL statement 5

Id	Result	#total	#true	#false	Execution time (min:sec)
1	TRUE	1	0	1	0:00.53
2	TRUE	1	0	1	0:00.53
3	TRUE	1	0	1	0:00.54
4	TRUE	1	0	1	0:00.53
5	TRUE	1	0	1	0:00.54
6	TRUE	1	0	1	0:00.54
7	TRUE	1	0	1	0:00.54
8	TRUE	1	0	1	0:00.54
9	TRUE	1	0	1	0:00.55
10	TRUE	1	0	1	0:00.54
11	TRUE	1	0	1	0:00.53
12	TRUE	1	0	1	0:00.54
13	TRUE	1	0	1	0:00.54
14	TRUE	1	0	1	0:00.54
15	TRUE	1	0	1	0:00.53
16	TRUE	1	0	1	0:00.54
17	TRUE	1	0	1	0:00.54
18	TRUE	1	0	1	0:00.53
19	TRUE	1	0	1	0:00.52

Id	Result	#total	#true	#false	Execution time (min:sec)
20	TRUE	1	0	1	0:00.53
21	TRUE	1	0	1	0:00.54
22	TRUE	1	0	1	0:00.52
23	TRUE	1	0	1	0:00.53
24	TRUE	1	0	1	0:00.54
25	TRUE	1	0	1	0:00.53
26	TRUE	1	0	1	0:00.53
27	TRUE	1	0	1	0:00.54
28	TRUE	1	0	1	0:00.52
29	TRUE	1	0	1	0:00.54
30	TRUE	1	0	1	0:00.53
31	TRUE	1	0	1	0:00.54
32	TRUE	1	0	1	0:00.52
33	TRUE	1	0	1	0:00.53
34	TRUE	1	0	1	0:00.53
35	TRUE	1	0	1	0:00.53
36	TRUE	1	0	1	0:00.54
37	TRUE	1	0	1	0:00.53
38	TRUE	1	0	1	0:00.54
39	TRUE	1	0	1	0:00.54
40	TRUE	1	0	1	0:00.54
41	TRUE	1	0	1	0:00.55
42	TRUE	1	0	1	0:00.54
43	TRUE	1	0	1	0:00.53
44	TRUE	1	0	1	0:00.54
45	TRUE	1	0	1	0:00.53
46	TRUE	1	0	1	0:00.54
47	TRUE	1	0	1	0:00.54
48	TRUE	1	0	1	0:00.53
49	TRUE	1	0	1	0:00.55
50	TRUE	1	0	1	0:00.54
51	TRUE	1	0	1	0:00.54
52	TRUE	1	0	1	0:00.54
53	TRUE	1	0	1	0:00.54
54	TRUE	1	0	1	0:00.54
55	TRUE	1	0	1	0:00.54
56	TRUE	1	0	1	0:00.54
57	TRUE	1	0	1	0:00.53
58	TRUE	1	0	1	0:00.54
59	TRUE	1	0	1	0:00.54
60	TRUE	1	0	1	0:00.54
61	TRUE	1	0	1	0:00.54
62	TRUE	1	0	1	0:00.54
63	TRUE	1	0	1	0:00.54
64	TRUE	1	0	1	0:00.53
65	TRUE	1	0	1	0:00.53
66	TRUE	1	0	1	0:00.54
67	TRUE	1	0	1	0:00.54

Id	Result	#total	#true	#false	Execution time (min:sec)
68	TRUE	1	0	1	0:00.54
69	TRUE	1	0	1	0:00.53
70	TRUE	1	0	1	0:00.54
71	TRUE	1	0	1	0:00.55
72	TRUE	1	0	1	0:00.54
73	TRUE	1	0	1	0:00.54
74	TRUE	1	0	1	0:00.54
75	TRUE	1	0	1	0:00.54
76	TRUE	1	0	1	0:00.55
77	TRUE	1	0	1	0:00.54
78	TRUE	1	0	1	0:00.54
79	TRUE	1	0	1	0:00.54
80	TRUE	1	0	1	0:00.52
81	TRUE	1	0	1	0:00.53
82	TRUE	1	0	1	0:00.53
83	TRUE	1	0	1	0:00.54
84	TRUE	1	0	1	0:00.54
85	TRUE	1	0	1	0:00.54
86	TRUE	1	0	1	0:00.54
87	TRUE	1	0	1	0:00.54
88	TRUE	1	0	1	0:00.54
89	TRUE	1	0	1	0:00.54
90	TRUE	1	0	1	0:00.54
91	TRUE	1	0	1	0:00.54
92	TRUE	1	0	1	0:00.53
93	TRUE	1	0	1	0:00.53
94	TRUE	1	0	1	0:00.54
95	TRUE	1	0	1	0:00.54
96	TRUE	1	0	1	0:00.54
97	TRUE	1	0	1	0:00.54
98	TRUE	1	0	1	0:00.54
99	TRUE	1	0	1	0:00.55
100	TRUE	1	0	1	0:00.54
101	TRUE	1	0	1	0:00.54
102	TRUE	1	0	1	0:00.53
103	TRUE	1	0	1	0:00.55
104	TRUE	1	0	1	0:00.54
105	TRUE	1	0	1	0:00.54
106	TRUE	1	0	1	0:00.54
107	TRUE	1	0	1	0:00.54
108	TRUE	1	0	1	0:00.54
109	TRUE	1	0	1	0:00.54
110	TRUE	1	0	1	0:00.54
111	TRUE	1	0	1	0:00.54
112	TRUE	1	0	1	0:00.54
113	TRUE	1	0	1	0:00.54
114	TRUE	1	0	1	0:00.54
115	TRUE	1	0	1	0:00.53

Id	Result	#total	#true	#false	Execution time (min:sec)
116	TRUE	1	0	1	0:00.54
117	TRUE	1	0	1	0:00.54
118	TRUE	1	0	1	0:00.55
119	TRUE	1	0	1	0:00.54
120	TRUE	1	0	1	0:00.54
121	TRUE	1	0	1	0:00.54
122	TRUE	1	0	1	0:00.54
123	TRUE	1	0	1	0:00.53
124	TRUE	1	0	1	0:00.55
125	TRUE	1	0	1	0:00.54
126	TRUE	1	0	1	0:00.55
127	TRUE	1	0	1	0:00.54
128	TRUE	1	0	1	0:00.54
129	TRUE	1	0	1	0:00.53
130	TRUE	1	0	1	0:00.55
131	TRUE	1	0	1	0:00.54
132	TRUE	1	0	1	0:00.53
133	TRUE	1	0	1	0:00.55
134	TRUE	1	0	1	0:00.54
135	TRUE	1	0	1	0:00.53
136	TRUE	1	0	1	0:00.54
137	TRUE	1	0	1	0:00.54
138	TRUE	1	0	1	0:00.54
139	TRUE	1	0	1	0:00.54
140	TRUE	1	0	1	0:00.53
141	TRUE	1	0	1	0:00.54
142	TRUE	1	0	1	0:00.56
143	TRUE	1	0	1	0:00.55
144	TRUE	1	0	1	0:00.54
145	TRUE	1	0	1	0:00.54
146	TRUE	1	0	1	0:00.53
147	TRUE	1	0	1	0:00.54
148	TRUE	1	0	1	0:00.54
149	TRUE	1	0	1	0:00.53
150	TRUE	1	0	1	0:00.52
151	TRUE	1	0	1	0:00.54
152	TRUE	1	0	1	0:00.54
153	TRUE	1	0	1	0:00.53
154	TRUE	1	0	1	0:00.54
155	TRUE	1	0	1	0:00.54
156	TRUE	1	0	1	0:00.54
157	TRUE	1	0	1	0:00.54
158	TRUE	1	0	1	0:00.53
159	TRUE	1	0	1	0:00.55
160	TRUE	1	0	1	0:00.53
161	TRUE	1	0	1	0:00.55
162	TRUE	1	0	1	0:00.54
163	TRUE	1	0	1	0:00.54

Id	Result	#total	#true	#false	Execution time (min:sec)
164	TRUE	1	0	1	0:00.54
165	TRUE	1	0	1	0:00.53
166	TRUE	1	0	1	0:00.52
167	TRUE	1	0	1	0:00.54
168	TRUE	1	0	1	0:00.53
169	TRUE	1	0	1	0:00.54
170	TRUE	1	0	1	0:00.54
171	TRUE	1	0	1	0:00.54
172	TRUE	1	0	1	0:00.54
173	TRUE	1	0	1	0:00.53
174	TRUE	1	0	1	0:00.53
175	TRUE	1	0	1	0:00.54
176	TRUE	1	0	1	0:00.53
177	TRUE	1	0	1	0:00.54
178	TRUE	1	0	1	0:00.53
179	TRUE	1	0	1	0:00.53
180	TRUE	1	0	1	0:00.53
181	TRUE	1	0	1	0:00.54
182	TRUE	1	0	1	0:00.53
183	TRUE	1	0	1	0:00.53
184	TRUE	1	0	1	0:00.54
185	TRUE	1	0	1	0:00.53
186	TRUE	1	0	1	0:00.53
187	TRUE	1	0	1	0:00.54
188	TRUE	1	0	1	0:00.53
189	TRUE	1	0	1	0:00.54
190	TRUE	1	0	1	0:00.54
191	TRUE	1	0	1	0:00.53
192	TRUE	1	0	1	0:00.53
193	TRUE	1	0	1	0:00.55
194	TRUE	1	0	1	0:00.54
195	TRUE	1	0	1	0:00.54
196	TRUE	1	0	1	0:00.53
197	TRUE	1	0	1	0:00.54
198	TRUE	1	0	1	0:00.53
199	TRUE	1	0	1	0:00.54
200	TRUE	1	0	1	0:00.54
201	TRUE	1	0	1	0:00.53
202	TRUE	1	0	1	0:00.53
203	TRUE	1	0	1	0:00.53
204	TRUE	1	0	1	0:00.54
205	TRUE	1	0	1	0:00.54
206	TRUE	1	0	1	0:00.54
207	TRUE	1	0	1	0:00.54
208	TRUE	1	0	1	0:00.54
209	TRUE	1	0	1	0:00.54
210	TRUE	1	0	1	0:00.54
211	TRUE	1	0	1	0:00.54

Id	Result	#total	#true	#false	Execution time (min:sec)
212	TRUE	1	0	1	0:00.54
213	TRUE	1	0	1	0:00.54
214	TRUE	1	0	1	0:00.53
215	TRUE	1	0	1	0:00.54
216	TRUE	1	0	1	0:00.52
217	TRUE	1	0	1	0:00.55
218	TRUE	1	0	1	0:00.54
219	TRUE	1	0	1	0:00.54
220	TRUE	1	0	1	0:00.54
221	TRUE	1	0	1	0:00.54
222	TRUE	1	0	1	0:00.54
223	TRUE	1	0	1	0:00.54
224	TRUE	1	0	1	0:00.54
225	TRUE	1	0	1	0:00.54
226	TRUE	1	0	1	0:00.52
227	TRUE	1	0	1	0:00.54
228	TRUE	1	0	1	0:00.55
229	TRUE	1	0	1	0:00.55
230	TRUE	1	0	1	0:00.54
231	TRUE	1	0	1	0:00.55
232	TRUE	1	0	1	0:00.54
233	TRUE	1	0	1	0:00.54
234	TRUE	1	0	1	0:00.53
235	TRUE	1	0	1	0:00.54
236	TRUE	1	0	1	0:00.54
237	TRUE	1	0	1	0:00.54
238	TRUE	1	0	1	0:00.54
239	TRUE	1	0	1	0:00.53
240	TRUE	1	0	1	0:00.54
241	TRUE	1	0	1	0:00.53
242	TRUE	1	0	1	0:00.53
243	TRUE	1	0	1	0:00.53
244	TRUE	1	0	1	0:00.54
245	TRUE	1	0	1	0:00.55
246	TRUE	1	0	1	0:00.54
247	TRUE	1	0	1	0:00.54
248	TRUE	1	0	1	0:00.53
249	TRUE	1	0	1	0:00.54
250	TRUE	1	0	1	0:00.54
251	TRUE	1	0	1	0:00.54
252	TRUE	1	0	1	0:00.53
253	TRUE	1	0	1	0:00.53
254	TRUE	1	0	1	0:00.52
255	TRUE	1	0	1	0:00.54
256	TRUE	1	0	1	0:00.53
257	TRUE	1	0	1	0:00.53
258	TRUE	1	0	1	0:00.54
259	TRUE	1	0	1	0:00.53

Id	Result	#total	#true	#false	Execution time (min:sec)
260	TRUE	1	0	1	0:00.55
261	TRUE	1	0	1	0:00.54
262	TRUE	1	0	1	0:00.54
263	TRUE	1	0	1	0:00.54
264	TRUE	1	0	1	0:00.54
265	TRUE	1	0	1	0:00.52
266	TRUE	1	0	1	0:00.53
267	TRUE	1	0	1	0:00.54
268	TRUE	1	0	1	0:00.54
269	TRUE	1	0	1	0:00.54
270	TRUE	1	0	1	0:00.52
271	TRUE	1	0	1	0:00.54
272	TRUE	1	0	1	0:00.54
273	TRUE	1	0	1	0:00.54
274	TRUE	1	0	1	0:00.54
275	TRUE	1	0	1	0:00.54
276	TRUE	1	0	1	0:00.54
277	TRUE	1	0	1	0:00.54
278	TRUE	1	0	1	0:00.54
279	TRUE	1	0	1	0:00.54
280	TRUE	1	0	1	0:00.53
281	TRUE	1	0	1	0:00.55
282	TRUE	1	0	1	0:00.54
283	TRUE	1	0	1	0:00.54
284	TRUE	1	0	1	0:00.53
285	TRUE	1	0	1	0:00.53
286	TRUE	1	0	1	0:00.53
287	TRUE	1	0	1	0:00.53
288	TRUE	1	0	1	0:00.53
289	TRUE	1	0	1	0:00.54
290	TRUE	1	0	1	0:00.54
291	TRUE	1	0	1	0:00.54
292	TRUE	1	0	1	0:00.54
293	TRUE	1	0	1	0:00.54
294	TRUE	1	0	1	0:00.54
295	TRUE	1	0	1	0:00.54
296	TRUE	1	0	1	0:00.54
297	TRUE	1	0	1	0:00.53
298	TRUE	1	0	1	0:00.54
299	TRUE	1	0	1	0:00.53
300	TRUE	1	0	1	0:00.54
301	TRUE	1	0	1	0:00.53
302	TRUE	1	0	1	0:00.52
303	TRUE	1	0	1	0:00.54
304	TRUE	1	0	1	0:00.54
305	TRUE	1	0	1	0:00.54
306	TRUE	1	0	1	0:00.53
307	TRUE	1	0	1	0:00.54

Id	Result	#total	#true	#false	Execution time (min:sec)
308	TRUE	1	0	1	0:00.54
309	TRUE	1	0	1	0:00.53
310	TRUE	1	0	1	0:00.54
311	TRUE	1	0	1	0:00.54
312	TRUE	1	0	1	0:00.53
313	TRUE	1	0	1	0:00.52
314	TRUE	1	0	1	0:00.55
315	TRUE	1	0	1	0:00.54
316	TRUE	1	0	1	0:00.54
317	TRUE	1	0	1	0:00.53
318	TRUE	1	0	1	0:00.54
319	TRUE	1	0	1	0:00.53
320	TRUE	1	0	1	0:00.54
321	TRUE	1	0	1	0:00.53
322	TRUE	1	0	1	0:00.54
323	TRUE	1	0	1	0:00.54
324	TRUE	1	0	1	0:00.54
325	TRUE	1	0	1	0:00.55
326	TRUE	1	0	1	0:00.54
327	TRUE	1	0	1	0:00.55
328	TRUE	1	0	1	0:00.54
329	TRUE	1	0	1	0:00.53
330	TRUE	1	0	1	0:00.54
331	TRUE	1	0	1	0:00.54
332	TRUE	1	0	1	0:00.54
333	TRUE	1	0	1	0:00.54
334	TRUE	1	0	1	0:00.54
335	TRUE	1	0	1	0:00.54
336	TRUE	1	0	1	0:00.54
337	TRUE	1	0	1	0:00.54
338	TRUE	1	0	1	0:00.54
339	TRUE	1	0	1	0:00.53
340	TRUE	1	0	1	0:00.54
341	TRUE	1	0	1	0:00.54
342	TRUE	1	0	1	0:00.53
343	TRUE	1	0	1	0:00.52
344	TRUE	1	0	1	0:00.54
345	TRUE	1	0	1	0:00.55
346	TRUE	1	0	1	0:00.54
347	TRUE	1	0	1	0:00.54
348	TRUE	1	0	1	0:00.53
349	TRUE	1	0	1	0:00.53
350	TRUE	1	0	1	0:00.54
351	TRUE	1	0	1	0:00.55
352	TRUE	1	0	1	0:00.54
353	TRUE	1	0	1	0:00.53
354	TRUE	1	0	1	0:00.54
355	TRUE	1	0	1	0:00.54

Id	Result	#total	#true	#false	Execution time (min:sec)
356	TRUE	1	0	1	0:00.53
357	TRUE	1	0	1	0:00.54
358	TRUE	1	0	1	0:00.53
359	TRUE	1	0	1	0:00.53
360	TRUE	1	0	1	0:00.53
361	TRUE	1	0	1	0:00.53
362	TRUE	1	0	1	0:00.53
363	TRUE	1	0	1	0:00.54
364	TRUE	1	0	1	0:00.54
365	TRUE	1	0	1	0:00.55
366	TRUE	1	0	1	0:00.54
367	TRUE	1	0	1	0:00.54
368	TRUE	1	0	1	0:00.54
369	TRUE	1	0	1	0:00.52
370	TRUE	1	0	1	0:00.54
371	TRUE	1	0	1	0:00.54
372	TRUE	1	0	1	0:00.54
373	TRUE	1	0	1	0:00.53
374	TRUE	1	0	1	0:00.54
375	TRUE	1	0	1	0:00.54
376	TRUE	1	0	1	0:00.54
377	TRUE	1	0	1	0:00.54
378	TRUE	1	0	1	0:00.54
379	TRUE	1	0	1	0:00.54
380	TRUE	1	0	1	0:00.54
381	TRUE	1	0	1	0:00.54
382	TRUE	1	0	1	0:00.54
383	TRUE	1	0	1	0:00.54
384	TRUE	1	0	1	0:00.53
385	TRUE	1	0	1	0:00.54
386	TRUE	1	0	1	0:00.54
387	TRUE	1	0	1	0:00.53
388	TRUE	1	0	1	0:00.54
389	TRUE	1	0	1	0:00.53
390	TRUE	1	0	1	0:00.54
391	TRUE	1	0	1	0:00.55
392	TRUE	1	0	1	0:00.54
393	TRUE	1	0	1	0:00.54
394	TRUE	1	0	1	0:00.54
395	TRUE	1	0	1	0:00.54
396	TRUE	1	0	1	0:00.53
397	TRUE	1	0	1	0:00.54
398	TRUE	1	0	1	0:00.54
399	TRUE	1	0	1	0:00.53
400	TRUE	1	0	1	0:00.53
401	TRUE	1	0	1	0:00.54
402	TRUE	1	0	1	0:00.53
403	TRUE	1	0	1	0:00.54

Id	Result	#total	#true	#false	Execution time (min:sec)
404	TRUE	1	0	1	0:00.53
405	TRUE	1	0	1	0:00.54
406	TRUE	1	0	1	0:00.54
407	TRUE	1	0	1	0:00.54
408	TRUE	1	0	1	0:00.54
409	TRUE	1	0	1	0:00.53
410	TRUE	1	0	1	0:00.54
411	TRUE	1	0	1	0:00.54
412	TRUE	1	0	1	0:00.53
413	TRUE	1	0	1	0:00.53
414	TRUE	1	0	1	0:00.54
415	TRUE	1	0	1	0:00.55
416	TRUE	1	0	1	0:00.54
417	TRUE	1	0	1	0:00.54
418	TRUE	1	0	1	0:00.54
419	TRUE	1	0	1	0:00.54
420	TRUE	1	0	1	0:00.54
421	TRUE	1	0	1	0:00.54
422	TRUE	1	0	1	0:00.54
423	TRUE	1	0	1	0:00.54
424	TRUE	1	0	1	0:00.55
425	TRUE	1	0	1	0:00.54
426	TRUE	1	0	1	0:00.53
427	TRUE	1	0	1	0:00.53
428	TRUE	1	0	1	0:00.54
429	TRUE	1	0	1	0:00.54
430	TRUE	1	0	1	0:00.54
431	TRUE	1	0	1	0:00.53
432	TRUE	1	0	1	0:00.53
433	TRUE	1	0	1	0:00.53
434	TRUE	1	0	1	0:00.53
435	TRUE	1	0	1	0:00.54
436	TRUE	1	0	1	0:00.53
437	TRUE	1	0	1	0:00.54
438	TRUE	1	0	1	0:00.54
439	TRUE	1	0	1	0:00.54
440	TRUE	1	0	1	0:00.52
441	TRUE	1	0	1	0:00.53
442	TRUE	1	0	1	0:00.54
443	TRUE	1	0	1	0:00.54
444	TRUE	1	0	1	0:00.54
445	TRUE	1	0	1	0:00.54
446	TRUE	1	0	1	0:00.54
447	TRUE	1	0	1	0:00.54
448	TRUE	1	0	1	0:00.55
449	TRUE	1	0	1	0:00.54
450	TRUE	1	0	1	0:00.53
451	TRUE	1	0	1	0:00.52

Id	Result	#total	#true	#false	Execution time (min:sec)
452	TRUE	1	0	1	0:00.54
453	TRUE	1	0	1	0:00.53
454	TRUE	1	0	1	0:00.54
455	TRUE	1	0	1	0:00.52
456	TRUE	1	0	1	0:00.54
457	TRUE	1	0	1	0:00.54
458	TRUE	1	0	1	0:00.53
459	TRUE	1	0	1	0:00.53
460	TRUE	1	0	1	0:00.53
461	TRUE	1	0	1	0:00.54
462	TRUE	1	0	1	0:00.54
463	TRUE	1	0	1	0:00.54
464	TRUE	1	0	1	0:00.54
465	TRUE	1	0	1	0:00.54
466	TRUE	1	0	1	0:00.54
467	TRUE	1	0	1	0:00.53
468	TRUE	1	0	1	0:00.54
469	TRUE	1	0	1	0:00.54
470	TRUE	1	0	1	0:00.54
471	TRUE	1	0	1	0:00.54
472	TRUE	1	0	1	0:00.55
473	TRUE	1	0	1	0:00.55
474	TRUE	1	0	1	0:00.54
475	TRUE	1	0	1	0:00.54
476	TRUE	1	0	1	0:00.53
477	TRUE	1	0	1	0:00.53
478	TRUE	1	0	1	0:00.54
479	TRUE	1	0	1	0:00.54
480	TRUE	1	0	1	0:00.54
481	TRUE	1	0	1	0:00.55
482	TRUE	1	0	1	0:00.53
483	TRUE	1	0	1	0:00.53
484	TRUE	1	0	1	0:00.54
485	TRUE	1	0	1	0:00.54
486	TRUE	1	0	1	0:00.54
487	TRUE	1	0	1	0:00.53
488	TRUE	1	0	1	0:00.54
489	TRUE	1	0	1	0:00.53
490	TRUE	1	0	1	0:00.53
491	TRUE	1	0	1	0:00.54
492	TRUE	1	0	1	0:00.52
493	TRUE	1	0	1	0:00.54
494	TRUE	1	0	1	0:00.54
495	TRUE	1	0	1	0:00.55
496	TRUE	1	0	1	0:00.53
497	TRUE	1	0	1	0:00.54
498	TRUE	1	0	1	0:00.53
499	TRUE	1	0	1	0:00.54

Id	Result	#total	#true	#false	Execution time (min:sec)
500	TRUE	1	0	1	0:00.54

Table 3: Model checking results corresponding to PBLMSTL statement 6

Id	Result	#total	#true	#false	Execution time (min:sec)
1	TRUE	1	1	0	0:00.54
2	TRUE	1	1	0	0:00.55
3	TRUE	1	1	0	0:00.55
4	TRUE	1	1	0	0:00.55
5	TRUE	1	1	0	0:00.54
6	TRUE	1	1	0	0:00.55
7	TRUE	1	1	0	0:00.55
8	TRUE	1	1	0	0:00.55
9	TRUE	1	1	0	0:00.55
10	TRUE	1	1	0	0:00.55
11	TRUE	1	1	0	0:00.55
12	TRUE	1	1	0	0:00.54
13	TRUE	1	1	0	0:00.54
14	TRUE	1	1	0	0:00.55
15	TRUE	1	1	0	0:00.55
16	TRUE	1	1	0	0:00.55
17	TRUE	1	1	0	0:00.55
18	TRUE	1	1	0	0:00.55
19	TRUE	1	1	0	0:00.54
20	TRUE	1	1	0	0:00.55
21	TRUE	1	1	0	0:00.54
22	TRUE	1	1	0	0:00.55
23	TRUE	1	1	0	0:00.55
24	TRUE	1	1	0	0:00.54
25	TRUE	1	1	0	0:00.55
26	TRUE	1	1	0	0:00.54
27	TRUE	1	1	0	0:00.55
28	TRUE	1	1	0	0:00.55
29	TRUE	1	1	0	0:00.55
30	TRUE	1	1	0	0:00.55
31	TRUE	1	1	0	0:00.55
32	TRUE	1	1	0	0:00.54
33	TRUE	1	1	0	0:00.55
34	TRUE	1	1	0	0:00.55
35	TRUE	1	1	0	0:00.54
36	TRUE	1	1	0	0:00.55
37	TRUE	1	1	0	0:00.55
38	TRUE	1	1	0	0:00.55
39	TRUE	1	1	0	0:00.55
40	TRUE	1	1	0	0:00.55

Id	Result	#total	#true	#false	Execution time (min:sec)
41	TRUE	1	1	0	0:00.55
42	TRUE	1	1	0	0:00.55
43	TRUE	1	1	0	0:00.55
44	TRUE	1	1	0	0:00.55
45	TRUE	1	1	0	0:00.55
46	TRUE	1	1	0	0:00.55
47	TRUE	1	1	0	0:00.53
48	TRUE	1	1	0	0:00.55
49	TRUE	1	1	0	0:00.54
50	TRUE	1	1	0	0:00.55
51	TRUE	1	1	0	0:00.54
52	TRUE	1	1	0	0:00.55
53	TRUE	1	1	0	0:00.54
54	TRUE	1	1	0	0:00.55
55	TRUE	1	1	0	0:00.55
56	TRUE	1	1	0	0:00.55
57	TRUE	1	1	0	0:00.54
58	TRUE	1	1	0	0:00.54
59	TRUE	1	1	0	0:00.55
60	TRUE	1	1	0	0:00.55
61	TRUE	1	1	0	0:00.55
62	TRUE	1	1	0	0:00.55
63	TRUE	1	1	0	0:00.56
64	TRUE	1	1	0	0:00.55
65	TRUE	1	1	0	0:00.54
66	TRUE	1	1	0	0:00.56
67	TRUE	1	1	0	0:00.54
68	TRUE	1	1	0	0:00.55
69	TRUE	1	1	0	0:00.55
70	TRUE	1	1	0	0:00.53
71	TRUE	1	1	0	0:00.55
72	TRUE	1	1	0	0:00.54
73	TRUE	1	1	0	0:00.55
74	TRUE	1	1	0	0:00.54
75	TRUE	1	1	0	0:00.56
76	TRUE	1	1	0	0:00.54
77	TRUE	1	1	0	0:00.55
78	TRUE	1	1	0	0:00.56
79	TRUE	1	1	0	0:00.55
80	TRUE	1	1	0	0:00.55
81	TRUE	1	1	0	0:00.55
82	TRUE	1	1	0	0:00.55
83	TRUE	1	1	0	0:00.54
84	TRUE	1	1	0	0:00.55
85	TRUE	1	1	0	0:00.55
86	TRUE	1	1	0	0:00.55
87	TRUE	1	1	0	0:00.55
88	TRUE	1	1	0	0:00.54

Id	Result	#total	#true	#false	Execution time (min:sec)
89	TRUE	1	1	0	0:00.55
90	TRUE	1	1	0	0:00.55
91	TRUE	1	1	0	0:00.55
92	TRUE	1	1	0	0:00.55
93	TRUE	1	1	0	0:00.54
94	TRUE	1	1	0	0:00.55
95	TRUE	1	1	0	0:00.53
96	TRUE	1	1	0	0:00.54
97	TRUE	1	1	0	0:00.54
98	TRUE	1	1	0	0:00.55
99	TRUE	1	1	0	0:00.55
100	TRUE	1	1	0	0:00.54
101	TRUE	1	1	0	0:00.54
102	TRUE	1	1	0	0:00.55
103	TRUE	1	1	0	0:00.55
104	TRUE	1	1	0	0:00.55
105	TRUE	1	1	0	0:00.55
106	TRUE	1	1	0	0:00.54
107	TRUE	1	1	0	0:00.55
108	TRUE	1	1	0	0:00.54
109	TRUE	1	1	0	0:00.55
110	TRUE	1	1	0	0:00.54
111	TRUE	1	1	0	0:00.55
112	TRUE	1	1	0	0:00.55
113	TRUE	1	1	0	0:00.55
114	TRUE	1	1	0	0:00.55
115	TRUE	1	1	0	0:00.54
116	TRUE	1	1	0	0:00.55
117	TRUE	1	1	0	0:00.55
118	TRUE	1	1	0	0:00.55
119	TRUE	1	1	0	0:00.55
120	TRUE	1	1	0	0:00.55
121	TRUE	1	1	0	0:00.55
122	TRUE	1	1	0	0:00.55
123	TRUE	1	1	0	0:00.55
124	TRUE	1	1	0	0:00.55
125	TRUE	1	1	0	0:00.55
126	TRUE	1	1	0	0:00.54
127	TRUE	1	1	0	0:00.55
128	TRUE	1	1	0	0:00.55
129	TRUE	1	1	0	0:00.55
130	TRUE	1	1	0	0:00.55
131	TRUE	1	1	0	0:00.54
132	TRUE	1	1	0	0:00.56
133	TRUE	1	1	0	0:00.55
134	TRUE	1	1	0	0:00.55
135	TRUE	1	1	0	0:00.55
136	TRUE	1	1	0	0:00.55

Id	Result	#total	#true	#false	Execution time (min:sec)
137	TRUE	1	1	0	0:00.56
138	TRUE	1	1	0	0:00.54
139	TRUE	1	1	0	0:00.55
140	TRUE	1	1	0	0:00.55
141	TRUE	1	1	0	0:00.55
142	TRUE	1	1	0	0:00.55
143	TRUE	1	1	0	0:00.55
144	TRUE	1	1	0	0:00.56
145	TRUE	1	1	0	0:00.55
146	TRUE	1	1	0	0:00.55
147	TRUE	1	1	0	0:00.54
148	TRUE	1	1	0	0:00.55
149	TRUE	1	1	0	0:00.56
150	TRUE	1	1	0	0:00.55
151	TRUE	1	1	0	0:00.55
152	TRUE	1	1	0	0:00.55
153	TRUE	1	1	0	0:00.55
154	TRUE	1	1	0	0:00.55
155	TRUE	1	1	0	0:00.55
156	TRUE	1	1	0	0:00.55
157	TRUE	1	1	0	0:00.54
158	TRUE	1	1	0	0:00.55
159	TRUE	1	1	0	0:00.54
160	TRUE	1	1	0	0:00.55
161	TRUE	1	1	0	0:00.55
162	TRUE	1	1	0	0:00.56
163	TRUE	1	1	0	0:00.54
164	TRUE	1	1	0	0:00.54
165	TRUE	1	1	0	0:00.55
166	TRUE	1	1	0	0:00.55
167	TRUE	1	1	0	0:00.55
168	TRUE	1	1	0	0:00.56
169	TRUE	1	1	0	0:00.55
170	TRUE	1	1	0	0:00.55
171	TRUE	1	1	0	0:00.54
172	TRUE	1	1	0	0:00.54
173	TRUE	1	1	0	0:00.56
174	TRUE	1	1	0	0:00.55
175	TRUE	1	1	0	0:00.55
176	TRUE	1	1	0	0:00.54
177	TRUE	1	1	0	0:00.55
178	TRUE	1	1	0	0:00.56
179	TRUE	1	1	0	0:00.55
180	TRUE	1	1	0	0:00.55
181	TRUE	1	1	0	0:00.55
182	TRUE	1	1	0	0:00.55
183	TRUE	1	1	0	0:00.54
184	TRUE	1	1	0	0:00.54

Id	Result	#total	#true	#false	Execution time (min:sec)
185	TRUE	1	1	0	0:00.55
186	TRUE	1	1	0	0:00.55
187	TRUE	1	1	0	0:00.55
188	TRUE	1	1	0	0:00.55
189	TRUE	1	1	0	0:00.55
190	TRUE	1	1	0	0:00.54
191	TRUE	1	1	0	0:00.55
192	TRUE	1	1	0	0:00.54
193	TRUE	1	1	0	0:00.55
194	TRUE	1	1	0	0:00.53
195	TRUE	1	1	0	0:00.55
196	TRUE	1	1	0	0:00.54
197	TRUE	1	1	0	0:00.55
198	TRUE	1	1	0	0:00.54
199	TRUE	1	1	0	0:00.55
200	TRUE	1	1	0	0:00.55
201	TRUE	1	1	0	0:00.55
202	TRUE	1	1	0	0:00.54
203	TRUE	1	1	0	0:00.54
204	TRUE	1	1	0	0:00.55
205	TRUE	1	1	0	0:00.55
206	TRUE	1	1	0	0:00.55
207	TRUE	1	1	0	0:00.56
208	TRUE	1	1	0	0:00.55
209	TRUE	1	1	0	0:00.55
210	TRUE	1	1	0	0:00.55
211	TRUE	1	1	0	0:00.55
212	TRUE	1	1	0	0:00.54
213	TRUE	1	1	0	0:00.55
214	TRUE	1	1	0	0:00.54
215	TRUE	1	1	0	0:00.54
216	TRUE	1	1	0	0:00.54
217	TRUE	1	1	0	0:00.55
218	TRUE	1	1	0	0:00.55
219	TRUE	1	1	0	0:00.55
220	TRUE	1	1	0	0:00.56
221	TRUE	1	1	0	0:00.55
222	TRUE	1	1	0	0:00.54
223	TRUE	1	1	0	0:00.55
224	TRUE	1	1	0	0:00.56
225	TRUE	1	1	0	0:00.57
226	TRUE	1	1	0	0:00.55
227	TRUE	1	1	0	0:00.55
228	TRUE	1	1	0	0:00.55
229	TRUE	1	1	0	0:00.55
230	TRUE	1	1	0	0:00.55
231	TRUE	1	1	0	0:00.55
232	TRUE	1	1	0	0:00.55

Id	Result	#total	#true	#false	Execution time (min:sec)
233	TRUE	1	1	0	0:00.55
234	TRUE	1	1	0	0:00.56
235	TRUE	1	1	0	0:00.55
236	TRUE	1	1	0	0:00.55
237	TRUE	1	1	0	0:00.55
238	TRUE	1	1	0	0:00.54
239	TRUE	1	1	0	0:00.55
240	TRUE	1	1	0	0:00.54
241	TRUE	1	1	0	0:00.56
242	TRUE	1	1	0	0:00.55
243	TRUE	1	1	0	0:00.54
244	TRUE	1	1	0	0:00.55
245	TRUE	1	1	0	0:00.54
246	TRUE	1	1	0	0:00.55
247	TRUE	1	1	0	0:00.55
248	TRUE	1	1	0	0:00.55
249	TRUE	1	1	0	0:00.55
250	TRUE	1	1	0	0:00.55
251	TRUE	1	1	0	0:00.54
252	TRUE	1	1	0	0:00.56
253	TRUE	1	1	0	0:00.54
254	TRUE	1	1	0	0:00.55
255	TRUE	1	1	0	0:00.53
256	TRUE	1	1	0	0:00.55
257	TRUE	1	1	0	0:00.55
258	TRUE	1	1	0	0:00.54
259	TRUE	1	1	0	0:00.54
260	TRUE	1	1	0	0:00.54
261	TRUE	1	1	0	0:00.55
262	TRUE	1	1	0	0:00.54
263	TRUE	1	1	0	0:00.54
264	TRUE	1	1	0	0:00.54
265	TRUE	1	1	0	0:00.55
266	TRUE	1	1	0	0:00.56
267	TRUE	1	1	0	0:00.56
268	TRUE	1	1	0	0:00.55
269	TRUE	1	1	0	0:00.54
270	TRUE	1	1	0	0:00.55
271	TRUE	1	1	0	0:00.55
272	TRUE	1	1	0	0:00.55
273	TRUE	1	1	0	0:00.56
274	TRUE	1	1	0	0:00.55
275	TRUE	1	1	0	0:00.55
276	TRUE	1	1	0	0:00.55
277	TRUE	1	1	0	0:00.55
278	TRUE	1	1	0	0:00.55
279	TRUE	1	1	0	0:00.56
280	TRUE	1	1	0	0:00.55

Id	Result	#total	#true	#false	Execution time (min:sec)
281	TRUE	1	1	0	0:00.55
282	TRUE	1	1	0	0:00.55
283	TRUE	1	1	0	0:00.55
284	TRUE	1	1	0	0:00.55
285	TRUE	1	1	0	0:00.55
286	TRUE	1	1	0	0:00.54
287	TRUE	1	1	0	0:00.55
288	TRUE	1	1	0	0:00.55
289	TRUE	1	1	0	0:00.55
290	TRUE	1	1	0	0:00.55
291	TRUE	1	1	0	0:00.56
292	TRUE	1	1	0	0:00.55
293	TRUE	1	1	0	0:00.54
294	TRUE	1	1	0	0:00.55
295	TRUE	1	1	0	0:00.55
296	TRUE	1	1	0	0:00.55
297	TRUE	1	1	0	0:00.55
298	TRUE	1	1	0	0:00.55
299	TRUE	1	1	0	0:00.54
300	TRUE	1	1	0	0:00.54
301	TRUE	1	1	0	0:00.55
302	TRUE	1	1	0	0:00.54
303	TRUE	1	1	0	0:00.55
304	TRUE	1	1	0	0:00.55
305	TRUE	1	1	0	0:00.53
306	TRUE	1	1	0	0:00.55
307	TRUE	1	1	0	0:00.55
308	TRUE	1	1	0	0:00.55
309	TRUE	1	1	0	0:00.55
310	TRUE	1	1	0	0:00.54
311	TRUE	1	1	0	0:00.55
312	TRUE	1	1	0	0:00.54
313	TRUE	1	1	0	0:00.55
314	TRUE	1	1	0	0:00.54
315	TRUE	1	1	0	0:00.56
316	TRUE	1	1	0	0:00.55
317	TRUE	1	1	0	0:00.54
318	TRUE	1	1	0	0:00.54
319	TRUE	1	1	0	0:00.55
320	TRUE	1	1	0	0:00.55
321	TRUE	1	1	0	0:00.55
322	TRUE	1	1	0	0:00.55
323	TRUE	1	1	0	0:00.56
324	TRUE	1	1	0	0:00.55
325	TRUE	1	1	0	0:00.55
326	TRUE	1	1	0	0:00.55
327	TRUE	1	1	0	0:00.55
328	TRUE	1	1	0	0:00.54

Id	Result	#total	#true	#false	Execution time (min:sec)
329	TRUE	1	1	0	0:00.54
330	TRUE	1	1	0	0:00.54
331	TRUE	1	1	0	0:00.55
332	TRUE	1	1	0	0:00.54
333	TRUE	1	1	0	0:00.55
334	TRUE	1	1	0	0:00.55
335	TRUE	1	1	0	0:00.54
336	TRUE	1	1	0	0:00.55
337	TRUE	1	1	0	0:00.54
338	TRUE	1	1	0	0:00.55
339	TRUE	1	1	0	0:00.54
340	TRUE	1	1	0	0:00.55
341	TRUE	1	1	0	0:00.55
342	TRUE	1	1	0	0:00.54
343	TRUE	1	1	0	0:00.54
344	TRUE	1	1	0	0:00.53
345	TRUE	1	1	0	0:00.56
346	TRUE	1	1	0	0:00.55
347	TRUE	1	1	0	0:00.55
348	TRUE	1	1	0	0:00.54
349	TRUE	1	1	0	0:00.55
350	TRUE	1	1	0	0:00.54
351	TRUE	1	1	0	0:00.55
352	TRUE	1	1	0	0:00.55
353	TRUE	1	1	0	0:00.55
354	TRUE	1	1	0	0:00.55
355	TRUE	1	1	0	0:00.55
356	TRUE	1	1	0	0:00.55
357	TRUE	1	1	0	0:00.55
358	TRUE	1	1	0	0:00.54
359	TRUE	1	1	0	0:00.55
360	TRUE	1	1	0	0:00.56
361	TRUE	1	1	0	0:00.55
362	TRUE	1	1	0	0:00.55
363	TRUE	1	1	0	0:00.55
364	TRUE	1	1	0	0:00.56
365	TRUE	1	1	0	0:00.56
366	TRUE	1	1	0	0:00.56
367	TRUE	1	1	0	0:00.55
368	TRUE	1	1	0	0:00.55
369	TRUE	1	1	0	0:00.55
370	TRUE	1	1	0	0:00.55
371	TRUE	1	1	0	0:00.55
372	TRUE	1	1	0	0:00.54
373	TRUE	1	1	0	0:00.55
374	TRUE	1	1	0	0:00.54
375	TRUE	1	1	0	0:00.55
376	TRUE	1	1	0	0:00.55

Id	Result	#total	#true	#false	Execution time (min:sec)
377	TRUE	1	1	0	0:00.55
378	TRUE	1	1	0	0:00.55
379	TRUE	1	1	0	0:00.55
380	TRUE	1	1	0	0:00.54
381	TRUE	1	1	0	0:00.55
382	TRUE	1	1	0	0:00.55
383	TRUE	1	1	0	0:00.55
384	TRUE	1	1	0	0:00.55
385	TRUE	1	1	0	0:00.54
386	TRUE	1	1	0	0:00.55
387	TRUE	1	1	0	0:00.54
388	TRUE	1	1	0	0:00.54
389	TRUE	1	1	0	0:00.55
390	TRUE	1	1	0	0:00.54
391	TRUE	1	1	0	0:00.55
392	TRUE	1	1	0	0:00.53
393	TRUE	1	1	0	0:00.55
394	TRUE	1	1	0	0:00.55
395	TRUE	1	1	0	0:00.55
396	TRUE	1	1	0	0:00.53
397	TRUE	1	1	0	0:00.55
398	TRUE	1	1	0	0:00.54
399	TRUE	1	1	0	0:00.55
400	TRUE	1	1	0	0:00.55
401	TRUE	1	1	0	0:00.55
402	TRUE	1	1	0	0:00.55
403	TRUE	1	1	0	0:00.55
404	TRUE	1	1	0	0:00.53
405	TRUE	1	1	0	0:00.55
406	TRUE	1	1	0	0:00.55
407	TRUE	1	1	0	0:00.57
408	TRUE	1	1	0	0:00.55
409	TRUE	1	1	0	0:00.56
410	TRUE	1	1	0	0:00.55
411	TRUE	1	1	0	0:00.54
412	TRUE	1	1	0	0:00.55
413	TRUE	1	1	0	0:00.55
414	TRUE	1	1	0	0:00.56
415	TRUE	1	1	0	0:00.55
416	TRUE	1	1	0	0:00.55
417	TRUE	1	1	0	0:00.54
418	TRUE	1	1	0	0:00.54
419	TRUE	1	1	0	0:00.54
420	TRUE	1	1	0	0:00.55
421	TRUE	1	1	0	0:00.55
422	TRUE	1	1	0	0:00.55
423	TRUE	1	1	0	0:00.55
424	TRUE	1	1	0	0:00.55

Id	Result	#total	#true	#false	Execution time (min:sec)
425	TRUE	1	1	0	0:00.55
426	TRUE	1	1	0	0:00.55
427	TRUE	1	1	0	0:00.54
428	TRUE	1	1	0	0:00.54
429	TRUE	1	1	0	0:00.54
430	TRUE	1	1	0	0:00.55
431	TRUE	1	1	0	0:00.55
432	TRUE	1	1	0	0:00.56
433	TRUE	1	1	0	0:00.55
434	TRUE	1	1	0	0:00.55
435	TRUE	1	1	0	0:00.55
436	TRUE	1	1	0	0:00.55
437	TRUE	1	1	0	0:00.55
438	TRUE	1	1	0	0:00.55
439	TRUE	1	1	0	0:00.54
440	TRUE	1	1	0	0:00.55
441	TRUE	1	1	0	0:00.55
442	TRUE	1	1	0	0:00.54
443	TRUE	1	1	0	0:00.56
444	TRUE	1	1	0	0:00.55
445	TRUE	1	1	0	0:00.55
446	TRUE	1	1	0	0:00.55
447	TRUE	1	1	0	0:00.54
448	TRUE	1	1	0	0:00.56
449	TRUE	1	1	0	0:00.53
450	TRUE	1	1	0	0:00.53
451	TRUE	1	1	0	0:00.56
452	TRUE	1	1	0	0:00.54
453	TRUE	1	1	0	0:00.55
454	TRUE	1	1	0	0:00.54
455	TRUE	1	1	0	0:00.55
456	TRUE	1	1	0	0:00.55
457	TRUE	1	1	0	0:00.55
458	TRUE	1	1	0	0:00.55
459	TRUE	1	1	0	0:00.56
460	TRUE	1	1	0	0:00.56
461	TRUE	1	1	0	0:00.56
462	TRUE	1	1	0	0:00.55
463	TRUE	1	1	0	0:00.55
464	TRUE	1	1	0	0:00.55
465	TRUE	1	1	0	0:00.54
466	TRUE	1	1	0	0:00.54
467	TRUE	1	1	0	0:00.55
468	TRUE	1	1	0	0:00.54
469	TRUE	1	1	0	0:00.55
470	TRUE	1	1	0	0:00.55
471	TRUE	1	1	0	0:00.55
472	TRUE	1	1	0	0:00.55

Id	Result	#total	#true	#false	Execution time (min:sec)
473	TRUE	1	1	0	0:00.55
474	TRUE	1	1	0	0:00.55
475	TRUE	1	1	0	0:00.55
476	TRUE	1	1	0	0:00.56
477	TRUE	1	1	0	0:00.55
478	TRUE	1	1	0	0:00.56
479	TRUE	1	1	0	0:00.54
480	TRUE	1	1	0	0:00.54
481	TRUE	1	1	0	0:00.56
482	TRUE	1	1	0	0:00.55
483	TRUE	1	1	0	0:00.56
484	TRUE	1	1	0	0:00.55
485	TRUE	1	1	0	0:00.55
486	TRUE	1	1	0	0:00.55
487	TRUE	1	1	0	0:00.55
488	TRUE	1	1	0	0:00.53
489	TRUE	1	1	0	0:00.55
490	TRUE	1	1	0	0:00.55
491	TRUE	1	1	0	0:00.54
492	TRUE	1	1	0	0:00.55
493	TRUE	1	1	0	0:00.55
494	TRUE	1	1	0	0:00.55
495	TRUE	1	1	0	0:00.55
496	TRUE	1	1	0	0:00.55
497	TRUE	1	1	0	0:00.55
498	TRUE	1	1	0	0:00.56
499	TRUE	1	1	0	0:00.55
500	TRUE	1	1	0	0:00.54
