# Supplementary material for: A Novel Method to Verify Multilevel Computational Models of Biological Systems Using Multiscale Spatio-Temporal Meta Model Checking
Source: PLoS One. 2016 May 17;11(5):e0154847. doi: 10.1371/journal.pone.0154847 (PMC4871515; doi:10.1371/journal.pone.0154847)
Supplement: S8 Text — (PDF) [file pone.0154847.s008.pdf]

Model checking results for the *Xenopus laevis* cell cycle case study

For the convenience of the reader the set of PBLMSTL statements considered for the *Xenopus laevis* cell cycle case study will be restated below:

$$P > 0.9\ [G\ [0,100]\ (((count(density(filter(regions, scaleAndSubsystem = Intracellular.CDK1 \wedge density < 0.96))) = count(density(filter(regions, scaleAndSubsystem = Intracellular.CDK1)))) \wedge (X\ (count(density(filter(regions, scaleAndSubsystem = Intracellular.CDK1 \wedge density > 0.96))) = count(density(filter(regions, scaleAndSubsystem = Intracellular.CDK1)))))) \Rightarrow (d(sum(multiply(area(filter(regions, scaleAndSubsystem = Cellular.Embryo)), density(filter(regions, scaleAndSubsystem = Cellular.Embryo))))) > 0))] \tag{7}$$

$$P > 0.9\ [G\ [0,100]\ (((d(avg(density(filter(regions, scaleAndSubsystem = Intracellular.APC)))) > 0)\ \wedge\ (X\ (d(avg(density(filter(regions, scaleAndSubsystem = Intracellular.APC)))) < 0)))\ \Rightarrow\ (X\ (d(sum(multiply(area(filter(regions, scaleAndSubsystem = Cellular.Embryo)), density(filter(regions, scaleAndSubsystem = Cellular.Embryo)))))) = 0)))] \tag{8}$$

$$
\begin{aligned}
P > 0.9\ & [(F\ [0,100]\ ((d(avg(density(\mathit{filter}(regions,\\
& \mathit{scaleAndSubsystem} = \mathit{Intracellular.CDK1}\\
&)))) > 0)\ \wedge\ (F\ [0,100]\ ((d(avg(density\\
& (\mathit{filter}(regions, \mathit{scaleAndSubsystem} =\\
& \mathit{Intracellular.CDK1})))) < 0)\ \wedge\\
& (F\ [0,100]\ ((d(avg(density(\mathit{filter}(regions,\\
& \mathit{scaleAndSubsystem} = \mathit{Intracellular.CDK1}\\
&)))) > 0)\ \wedge\ (F\ [0,100]\ ((d(avg(density\\
& (\mathit{filter}(regions, \mathit{scaleAndSubsystem} =\\
& \mathit{Intracellular.CDK1})))) < 0)\ \wedge\\
& (F\ [0,100]\ ((d(avg(density(\mathit{filter}(regions,\\
& \mathit{scaleAndSubsystem} = \mathit{Intracellular.CDK1}\\
&)))) > 0)\ \wedge\ (F\ [0,100]\ ((d(avg(density\\
& (\mathit{filter}(regions, \mathit{scaleAndSubsystem} =\\
& \mathit{Intracellular.CDK1})))) < 0)\\
&))))))))))))\ \wedge\\
& (F\ [0,100]\ ((d(avg(density(\mathit{filter}(regions,\\
& \mathit{scaleAndSubsystem} = \mathit{Intracellular.Plk1}\\
&)))) > 0)\ \wedge\ (F\ [0,100]\ ((d(avg(density\\
& (\mathit{filter}(regions, \mathit{scaleAndSubsystem} =\\
& \mathit{Intracellular.Plk1})))) < 0)\ \wedge\\
& (F\ [0,100]\ ((d(avg(density(\mathit{filter}(regions,\\
& \mathit{scaleAndSubsystem} = \mathit{Intracellular.Plk1}\\
&)))) > 0)\ \wedge\ (F\ [0,100]\ ((d(avg(density\\
& (\mathit{filter}(regions, \mathit{scaleAndSubsystem} =\\
& \mathit{Intracellular.Plk1})))) < 0)\ \wedge\\
& (F\ [0,100]\ ((d(avg(density(\mathit{filter}(regions,\\
& \mathit{scaleAndSubsystem} = \mathit{Intracellular.Plk1}\\
&)))) > 0)\ \wedge
\end{aligned}
\tag{9}
$$

$$
\begin{array}{l}
(F\ [0,100]\ ((d(avg(density \\
(filter(regions, scaleAndSubsystem = \\
Intracellular.Plk1)))) < 0) \\
)))))))))))) \wedge \\
(F\ [0,100]\ ((d(avg(density(filter(regions, \\
scaleAndSubsystem = Intracellular.APC \\
)))) > 0)\ \wedge\ (F\ [0,100]\ ((d(avg(density \\
(filter(regions, scaleAndSubsystem = \\
Intracellular.APC)))) < 0) \wedge \\
(F\ [0,100]\ ((d(avg(density(filter(regions, \\
scaleAndSubsystem = Intracellular.APC \\
)))) > 0)\ \wedge\ (F\ [0,100]\ ((d(avg(density \\
(filter(regions, scaleAndSubsystem = \\
Intracellular.APC)))) < 0) \wedge \\
(F\ [0,100]\ ((d(avg(density(filter(regions, \\
scaleAndSubsystem = Intracellular.APC \\
)))) > 0)\ \wedge\ (F\ [0,100]\ ((d(avg(density \\
(filter(regions, scaleAndSubsystem = \\
Intracellular.APC)))) < 0) \\
))))))))))))]
\end{array}
$$

Each table describes the results corresponding to one of the PBLMSTL statements. The first column of each row represents the identifier of the model checking execution. The second column represents the evaluation result (T = true, F = false) of the PBLMSTL statement for that particular model checker execution. The number of MSTML files against which the PBLMSTL statement was executed, respectively how many of them evaluated true and how many evaluated false is provided in columns three, four and five. Finally column six presents the execution time (minutes:seconds format) corresponding to each model checker run. All executions of the model checker employed the frequentist statistical model checking approach with probability of both type I and type II errors equal to 5%.

Table 1: Model checking results corresponding to PBLMSTL statement 7

**Id**	**Result**	**#total**	**#true**	**#false**	**Execution time (min:sec)**
1	TRUE	28	28	0	0:35.35
2	TRUE	32	31	1	0:39.45
3	TRUE	28	28	0	0:34.74
4	TRUE	28	28	0	0:35.05
5	TRUE	28	28	0	0:34.54
6	TRUE	28	28	0	0:34.65
7	TRUE	28	28	0	0:34.71

Id	Result	#total	#true	#false	Execution time (min:sec)
8	TRUE	28	28	0	0:34.78
9	TRUE	28	28	0	0:34.79
10	TRUE	28	28	0	0:34.81
11	TRUE	28	28	0	0:34.54
12	TRUE	28	28	0	0:34.46
13	TRUE	32	31	1	0:40.12
14	TRUE	28	28	0	0:35.99
15	TRUE	28	28	0	0:35.82
16	TRUE	28	28	0	0:35.62
17	TRUE	28	28	0	0:34.66
18	TRUE	32	31	1	0:39.36
19	TRUE	32	31	1	0:39.49
20	TRUE	28	28	0	0:34.66
21	TRUE	28	28	0	0:34.77
22	TRUE	28	28	0	0:34.74
23	TRUE	28	28	0	0:34.67
24	TRUE	28	28	0	0:34.55
25	TRUE	28	28	0	0:34.59
26	TRUE	28	28	0	0:34.55
27	TRUE	28	28	0	0:34.44
28	TRUE	28	28	0	0:34.51
29	TRUE	39	37	2	0:47.65
30	TRUE	28	28	0	0:34.40
31	TRUE	28	28	0	0:34.56
32	TRUE	28	28	0	0:34.52
33	TRUE	28	28	0	0:34.63
34	TRUE	28	28	0	0:34.43
35	TRUE	28	28	0	0:34.54
36	TRUE	32	31	1	0:39.13
37	TRUE	32	31	1	0:39.36
38	TRUE	32	31	1	0:39.24
39	TRUE	28	28	0	0:34.43
40	TRUE	28	28	0	0:34.56
41	TRUE	28	28	0	0:34.45
42	TRUE	28	28	0	0:34.53
43	TRUE	28	28	0	0:34.65
44	TRUE	32	31	1	0:39.15
45	TRUE	28	28	0	0:34.50
46	TRUE	28	28	0	0:34.67
47	TRUE	28	28	0	0:34.55
48	TRUE	28	28	0	0:34.44
49	TRUE	28	28	0	0:34.42
50	TRUE	28	28	0	0:34.50
51	TRUE	28	28	0	0:34.49
52	TRUE	28	28	0	0:34.38
53	TRUE	28	28	0	0:34.57
54	TRUE	28	28	0	0:34.73
55	TRUE	28	28	0	0:34.48

Id	Result	#total	#true	#false	Execution time (min:sec)
56	TRUE	28	28	0	0:34.53
57	TRUE	32	31	1	0:39.26
58	TRUE	28	28	0	0:34.37
59	TRUE	28	28	0	0:34.42
60	TRUE	28	28	0	0:34.54
61	TRUE	28	28	0	0:34.39
62	TRUE	28	28	0	0:34.43
63	TRUE	28	28	0	0:34.57
64	TRUE	28	28	0	0:34.54
65	TRUE	28	28	0	0:34.40
66	TRUE	28	28	0	0:34.41
67	TRUE	28	28	0	0:34.59
68	TRUE	28	28	0	0:34.46
69	TRUE	28	28	0	0:34.46
70	TRUE	32	31	1	0:39.36
71	TRUE	28	28	0	0:34.54
72	TRUE	28	28	0	0:34.39
73	TRUE	28	28	0	0:34.35
74	TRUE	28	28	0	0:34.34
75	TRUE	28	28	0	0:34.50
76	TRUE	39	37	2	0:47.32
77	TRUE	28	28	0	0:34.73
78	TRUE	28	28	0	0:34.25
79	TRUE	28	28	0	0:34.38
80	TRUE	28	28	0	0:34.38
81	TRUE	28	28	0	0:34.32
82	TRUE	28	28	0	0:34.19
83	TRUE	28	28	0	0:34.46
84	TRUE	28	28	0	0:34.30
85	TRUE	28	28	0	0:34.33
86	TRUE	28	28	0	0:34.46
87	TRUE	28	28	0	0:34.26
88	TRUE	28	28	0	0:34.41
89	TRUE	28	28	0	0:34.34
90	TRUE	28	28	0	0:34.27
91	TRUE	28	28	0	0:34.42
92	TRUE	28	28	0	0:34.37
93	TRUE	28	28	0	0:34.28
94	TRUE	28	28	0	0:34.44
95	TRUE	28	28	0	0:34.17
96	TRUE	28	28	0	0:34.33
97	TRUE	28	28	0	0:34.42
98	TRUE	28	28	0	0:34.25
99	TRUE	28	28	0	0:34.29
100	TRUE	28	28	0	0:34.37
101	TRUE	28	28	0	0:34.25
102	TRUE	28	28	0	0:34.31
103	TRUE	28	28	0	0:34.39

Id	Result	#total	#true	#false	Execution time (min:sec)
104	TRUE	28	28	0	0:34.21
105	TRUE	28	28	0	0:34.28
106	TRUE	28	28	0	0:34.29
107	TRUE	32	31	1	0:39.00
108	TRUE	28	28	0	0:34.40
109	TRUE	39	37	2	0:47.56
110	TRUE	32	31	1	0:39.22
111	TRUE	28	28	0	0:34.38
112	TRUE	28	28	0	0:34.23
113	TRUE	28	28	0	0:34.43
114	TRUE	28	28	0	0:34.31
115	TRUE	28	28	0	0:34.21
116	TRUE	28	28	0	0:34.18
117	TRUE	28	28	0	0:34.23
118	TRUE	28	28	0	0:34.43
119	TRUE	28	28	0	0:34.30
120	TRUE	28	28	0	0:34.37
121	TRUE	28	28	0	0:34.60
122	TRUE	28	28	0	0:34.25
123	TRUE	28	28	0	0:34.28
124	TRUE	28	28	0	0:34.15
125	TRUE	28	28	0	0:34.44
126	TRUE	28	28	0	0:34.33
127	TRUE	28	28	0	0:34.34
128	TRUE	32	31	1	0:38.99
129	TRUE	28	28	0	0:34.31
130	TRUE	28	28	0	0:34.44
131	TRUE	32	31	1	0:39.18
132	TRUE	28	28	0	0:34.35
133	TRUE	28	28	0	0:34.34
134	TRUE	28	28	0	0:34.35
135	TRUE	28	28	0	0:34.30
136	TRUE	28	28	0	0:34.69
137	TRUE	28	28	0	0:34.46
138	TRUE	28	28	0	0:34.38
139	TRUE	28	28	0	0:34.32
140	TRUE	28	28	0	0:34.24
141	TRUE	28	28	0	0:34.30
142	TRUE	28	28	0	0:34.31
143	TRUE	32	31	1	0:39.02
144	TRUE	28	28	0	0:34.37
145	TRUE	28	28	0	0:34.26
146	TRUE	28	28	0	0:34.32
147	TRUE	28	28	0	0:34.28
148	TRUE	32	31	1	0:39.05
149	TRUE	28	28	0	0:34.12
150	TRUE	28	28	0	0:34.36
151	TRUE	28	28	0	0:34.22

Id	Result	#total	#true	#false	Execution time (min:sec)
152	TRUE	28	28	0	0:34.29
153	TRUE	32	31	1	0:39.02
154	TRUE	28	28	0	0:34.24
155	TRUE	28	28	0	0:34.35
156	TRUE	28	28	0	0:34.35
157	TRUE	32	31	1	0:39.12
158	TRUE	28	28	0	0:34.21
159	TRUE	28	28	0	0:34.13
160	TRUE	32	31	1	0:39.23
161	TRUE	28	28	0	0:34.19
162	TRUE	28	28	0	0:34.35
163	TRUE	28	28	0	0:34.26
164	TRUE	28	28	0	0:34.39
165	TRUE	28	28	0	0:34.38
166	TRUE	28	28	0	0:34.54
167	TRUE	28	28	0	0:34.46
168	TRUE	28	28	0	0:34.19
169	TRUE	28	28	0	0:34.26
170	TRUE	28	28	0	0:34.45
171	TRUE	28	28	0	0:34.35
172	TRUE	28	28	0	0:34.29
173	TRUE	32	31	1	0:38.99
174	TRUE	28	28	0	0:34.44
175	TRUE	28	28	0	0:34.28
176	TRUE	28	28	0	0:34.36
177	TRUE	28	28	0	0:34.33
178	TRUE	28	28	0	0:34.32
179	TRUE	28	28	0	0:34.28
180	TRUE	28	28	0	0:34.48
181	TRUE	28	28	0	0:34.21
182	TRUE	28	28	0	0:34.33
183	TRUE	28	28	0	0:34.34
184	TRUE	32	31	1	0:39.04
185	TRUE	28	28	0	0:34.42
186	TRUE	28	28	0	0:34.30
187	TRUE	28	28	0	0:34.23
188	TRUE	28	28	0	0:34.27
189	TRUE	28	28	0	0:34.26
190	TRUE	28	28	0	0:34.23
191	TRUE	39	37	2	0:47.21
192	TRUE	28	28	0	0:34.17
193	TRUE	32	31	1	0:39.27
194	TRUE	32	31	1	0:38.86
195	TRUE	28	28	0	0:34.24
196	TRUE	28	28	0	0:34.27
197	TRUE	28	28	0	0:34.38
198	TRUE	28	28	0	0:34.28
199	TRUE	28	28	0	0:34.44

Id	Result	#total	#true	#false	Execution time (min:sec)
200	TRUE	28	28	0	0:34.28
201	TRUE	28	28	0	0:34.22
202	TRUE	28	28	0	0:34.24
203	TRUE	28	28	0	0:34.21
204	TRUE	28	28	0	0:34.28
205	TRUE	28	28	0	0:34.29
206	TRUE	28	28	0	0:34.30
207	TRUE	28	28	0	0:34.22
208	TRUE	28	28	0	0:34.39
209	TRUE	28	28	0	0:34.23
210	TRUE	28	28	0	0:34.20
211	TRUE	32	31	1	0:38.90
212	TRUE	28	28	0	0:34.20
213	TRUE	28	28	0	0:34.29
214	TRUE	28	28	0	0:34.22
215	TRUE	28	28	0	0:34.23
216	TRUE	32	31	1	0:38.99
217	TRUE	28	28	0	0:34.45
218	TRUE	28	28	0	0:34.28
219	TRUE	28	28	0	0:34.32
220	TRUE	28	28	0	0:34.50
221	TRUE	28	28	0	0:34.26
222	TRUE	28	28	0	0:34.29
223	TRUE	28	28	0	0:34.22
224	TRUE	28	28	0	0:34.36
225	TRUE	28	28	0	0:34.20
226	TRUE	28	28	0	0:34.28
227	TRUE	28	28	0	0:34.31
228	TRUE	28	28	0	0:34.21
229	TRUE	28	28	0	0:34.46
230	TRUE	28	28	0	0:34.35
231	TRUE	32	31	1	0:38.93
232	TRUE	32	31	1	0:38.90
233	TRUE	28	28	0	0:34.18
234	TRUE	28	28	0	0:34.20
235	TRUE	28	28	0	0:34.29
236	TRUE	28	28	0	0:34.35
237	TRUE	28	28	0	0:34.21
238	TRUE	28	28	0	0:34.15
239	TRUE	28	28	0	0:34.31
240	TRUE	28	28	0	0:34.28
241	TRUE	28	28	0	0:34.33
242	TRUE	28	28	0	0:34.30
243	TRUE	28	28	0	0:34.18
244	TRUE	28	28	0	0:34.21
245	TRUE	28	28	0	0:34.17
246	TRUE	39	37	2	0:47.26
247	TRUE	28	28	0	0:34.23

Id	Result	#total	#true	#false	Execution time (min:sec)
248	TRUE	28	28	0	0:34.18
249	TRUE	32	31	1	0:39.07
250	TRUE	28	28	0	0:34.17
251	TRUE	28	28	0	0:34.22
252	TRUE	28	28	0	0:34.37
253	TRUE	32	31	1	0:38.86
254	TRUE	28	28	0	0:34.26
255	TRUE	28	28	0	0:34.14
256	TRUE	28	28	0	0:34.13
257	TRUE	28	28	0	0:34.21
258	TRUE	32	31	1	0:39.24
259	TRUE	28	28	0	0:34.32
260	TRUE	28	28	0	0:34.28
261	TRUE	28	28	0	0:34.27
262	TRUE	28	28	0	0:34.24
263	TRUE	28	28	0	0:34.25
264	TRUE	28	28	0	0:34.36
265	TRUE	28	28	0	0:34.25
266	TRUE	28	28	0	0:34.27
267	TRUE	28	28	0	0:34.28
268	TRUE	28	28	0	0:34.24
269	TRUE	32	31	1	0:39.05
270	TRUE	28	28	0	0:34.29
271	TRUE	28	28	0	0:34.28
272	TRUE	32	31	1	0:38.95
273	TRUE	28	28	0	0:34.28
274	TRUE	28	28	0	0:34.23
275	TRUE	28	28	0	0:34.33
276	TRUE	28	28	0	0:34.32
277	TRUE	28	28	0	0:34.19
278	TRUE	28	28	0	0:34.14
279	TRUE	28	28	0	0:34.29
280	TRUE	39	37	2	0:47.28
281	TRUE	28	28	0	0:34.29
282	TRUE	28	28	0	0:34.34
283	TRUE	28	28	0	0:34.38
284	TRUE	28	28	0	0:34.58
285	TRUE	28	28	0	0:34.21
286	TRUE	32	31	1	0:38.99
287	TRUE	39	37	2	0:47.36
288	TRUE	28	28	0	0:34.38
289	TRUE	28	28	0	0:34.29
290	TRUE	28	28	0	0:34.33
291	TRUE	28	28	0	0:34.18
292	TRUE	32	31	1	0:39.11
293	TRUE	28	28	0	0:34.09
294	TRUE	28	28	0	0:34.20
295	TRUE	28	28	0	0:34.35

Id	Result	#total	#true	#false	Execution time (min:sec)
296	TRUE	32	31	1	0:39.09
297	TRUE	28	28	0	0:34.37
298	TRUE	28	28	0	0:34.30
299	TRUE	28	28	0	0:34.38
300	TRUE	28	28	0	0:34.22
301	TRUE	28	28	0	0:34.28
302	TRUE	28	28	0	0:34.36
303	TRUE	28	28	0	0:34.31
304	TRUE	28	28	0	0:34.33
305	TRUE	28	28	0	0:34.34
306	TRUE	28	28	0	0:34.28
307	TRUE	28	28	0	0:34.39
308	TRUE	28	28	0	0:34.27
309	TRUE	32	31	1	0:38.95
310	TRUE	28	28	0	0:34.19
311	TRUE	28	28	0	0:34.31
312	TRUE	28	28	0	0:34.43
313	TRUE	28	28	0	0:34.31
314	TRUE	28	28	0	0:34.22
315	TRUE	32	31	1	0:38.91
316	TRUE	32	31	1	0:39.09
317	TRUE	28	28	0	0:34.26
318	TRUE	28	28	0	0:34.18
319	TRUE	28	28	0	0:34.34
320	TRUE	28	28	0	0:34.26
321	TRUE	28	28	0	0:34.25
322	TRUE	28	28	0	0:34.36
323	TRUE	28	28	0	0:34.31
324	TRUE	28	28	0	0:34.35
325	TRUE	28	28	0	0:34.16
326	TRUE	28	28	0	0:34.34
327	TRUE	28	28	0	0:34.32
328	TRUE	28	28	0	0:34.24
329	TRUE	28	28	0	0:34.16
330	TRUE	28	28	0	0:34.26
331	TRUE	28	28	0	0:34.27
332	TRUE	28	28	0	0:34.24
333	TRUE	28	28	0	0:34.20
334	TRUE	28	28	0	0:34.09
335	TRUE	28	28	0	0:34.22
336	TRUE	28	28	0	0:34.34
337	TRUE	28	28	0	0:34.31
338	TRUE	28	28	0	0:34.49
339	TRUE	32	31	1	0:39.07
340	TRUE	28	28	0	0:34.28
341	TRUE	28	28	0	0:34.26
342	TRUE	32	31	1	0:39.11
343	TRUE	28	28	0	0:34.31

Id	Result	#total	#true	#false	Execution time (min:sec)
344	TRUE	32	31	1	0:39.02
345	TRUE	28	28	0	0:34.19
346	TRUE	28	28	0	0:34.28
347	TRUE	28	28	0	0:34.32
348	TRUE	28	28	0	0:34.34
349	TRUE	28	28	0	0:34.12
350	TRUE	28	28	0	0:34.41
351	TRUE	28	28	0	0:34.28
352	TRUE	39	37	2	0:47.19
353	TRUE	28	28	0	0:34.33
354	TRUE	32	31	1	0:38.97
355	TRUE	28	28	0	0:34.60
356	TRUE	28	28	0	0:34.15
357	TRUE	32	31	1	0:38.93
358	TRUE	28	28	0	0:34.40
359	TRUE	32	31	1	0:38.84
360	TRUE	32	31	1	0:39.17
361	TRUE	28	28	0	0:34.32
362	TRUE	28	28	0	0:34.29
363	TRUE	28	28	0	0:34.23
364	TRUE	32	31	1	0:38.98
365	TRUE	28	28	0	0:34.47
366	TRUE	28	28	0	0:34.25
367	TRUE	28	28	0	0:34.23
368	TRUE	28	28	0	0:34.39
369	TRUE	32	31	1	0:38.87
370	TRUE	28	28	0	0:34.34
371	TRUE	28	28	0	0:34.20
372	TRUE	28	28	0	0:34.34
373	TRUE	28	28	0	0:34.38
374	TRUE	28	28	0	0:34.25
375	TRUE	28	28	0	0:34.17
376	TRUE	28	28	0	0:34.34
377	TRUE	32	31	1	0:39.03
378	TRUE	28	28	0	0:34.41
379	TRUE	39	37	2	0:47.24
380	TRUE	28	28	0	0:34.21
381	TRUE	28	28	0	0:34.21
382	TRUE	28	28	0	0:34.26
383	TRUE	28	28	0	0:34.17
384	TRUE	32	31	1	0:39.03
385	TRUE	28	28	0	0:34.78
386	TRUE	28	28	0	0:34.73
387	TRUE	28	28	0	0:34.30
388	TRUE	28	28	0	0:34.35
389	TRUE	28	28	0	0:34.25
390	TRUE	28	28	0	0:34.25
391	TRUE	28	28	0	0:34.38

Id	Result	#total	#true	#false	Execution time (min:sec)
392	TRUE	39	37	2	0:47.20
393	TRUE	28	28	0	0:34.20
394	TRUE	32	31	1	0:38.89
395	TRUE	32	31	1	0:38.86
396	TRUE	28	28	0	0:34.19
397	TRUE	28	28	0	0:34.21
398	TRUE	28	28	0	0:34.39
399	TRUE	28	28	0	0:34.73
400	TRUE	28	28	0	0:34.22
401	TRUE	28	28	0	0:34.32
402	TRUE	28	28	0	0:34.30
403	TRUE	28	28	0	0:34.25
404	TRUE	32	31	1	0:39.02
405	TRUE	28	28	0	0:34.30
406	TRUE	39	37	2	0:47.18
407	TRUE	28	28	0	0:34.42
408	TRUE	28	28	0	0:34.23
409	TRUE	28	28	0	0:34.28
410	TRUE	28	28	0	0:34.36
411	TRUE	28	28	0	0:34.38
412	TRUE	28	28	0	0:34.62
413	TRUE	32	31	1	0:39.73
414	TRUE	28	28	0	0:34.68
415	TRUE	28	28	0	0:34.44
416	TRUE	28	28	0	0:34.36
417	TRUE	28	28	0	0:34.21
418	TRUE	39	37	2	0:47.26
419	TRUE	28	28	0	0:34.25
420	TRUE	28	28	0	0:34.20
421	TRUE	28	28	0	0:34.25
422	TRUE	28	28	0	0:34.35
423	TRUE	28	28	0	0:34.28
424	TRUE	28	28	0	0:34.33
425	TRUE	28	28	0	0:34.27
426	TRUE	32	31	1	0:39.05
427	TRUE	32	31	1	0:39.14
428	TRUE	28	28	0	0:34.31
429	TRUE	28	28	0	0:34.43
430	TRUE	32	31	1	0:39.07
431	TRUE	28	28	0	0:34.34
432	TRUE	28	28	0	0:34.26
433	TRUE	28	28	0	0:34.28
434	TRUE	28	28	0	0:34.22
435	TRUE	28	28	0	0:34.35
436	TRUE	32	31	1	0:38.88
437	TRUE	28	28	0	0:34.48
438	TRUE	32	31	1	0:39.00
439	TRUE	28	28	0	0:34.17

Id	Result	#total	#true	#false	Execution time (min:sec)
440	TRUE	28	28	0	0:34.29
441	TRUE	28	28	0	0:34.24
442	TRUE	28	28	0	0:34.25
443	TRUE	28	28	0	0:34.30
444	TRUE	28	28	0	0:34.27
445	TRUE	28	28	0	0:34.29
446	TRUE	28	28	0	0:34.15
447	TRUE	28	28	0	0:34.29
448	TRUE	28	28	0	0:34.16
449	TRUE	28	28	0	0:34.21
450	TRUE	28	28	0	0:34.24
451	TRUE	28	28	0	0:34.16
452	TRUE	28	28	0	0:34.35
453	TRUE	28	28	0	0:34.28
454	TRUE	32	31	1	0:38.90
455	TRUE	28	28	0	0:34.16
456	TRUE	28	28	0	0:34.31
457	TRUE	28	28	0	0:34.23
458	TRUE	28	28	0	0:34.15
459	TRUE	28	28	0	0:34.31
460	TRUE	28	28	0	0:34.18
461	TRUE	28	28	0	0:34.15
462	TRUE	28	28	0	0:34.23
463	TRUE	28	28	0	0:34.33
464	TRUE	28	28	0	0:34.43
465	TRUE	28	28	0	0:34.29
466	TRUE	32	31	1	0:39.06
467	TRUE	28	28	0	0:34.20
468	TRUE	32	31	1	0:39.01
469	TRUE	28	28	0	0:34.36
470	TRUE	28	28	0	0:34.19
471	TRUE	28	28	0	0:34.24
472	TRUE	28	28	0	0:34.33
473	TRUE	32	31	1	0:38.96
474	TRUE	32	31	1	0:39.09
475	TRUE	28	28	0	0:34.29
476	TRUE	28	28	0	0:34.42
477	TRUE	32	31	1	0:38.98
478	TRUE	28	28	0	0:34.17
479	TRUE	28	28	0	0:34.37
480	TRUE	28	28	0	0:34.25
481	TRUE	28	28	0	0:34.10
482	TRUE	28	28	0	0:34.22
483	TRUE	28	28	0	0:34.32
484	TRUE	28	28	0	0:34.43
485	TRUE	28	28	0	0:34.31
486	TRUE	32	31	1	0:39.06
487	TRUE	32	31	1	0:39.02

Id	Result	#total	#true	#false	Execution time (min:sec)
488	TRUE	28	28	0	0:34.22
489	TRUE	28	28	0	0:34.12
490	TRUE	28	28	0	0:34.21
491	TRUE	28	28	0	0:34.20
492	TRUE	28	28	0	0:34.35
493	TRUE	28	28	0	0:34.32
494	TRUE	28	28	0	0:34.27
495	TRUE	28	28	0	0:34.21
496	TRUE	28	28	0	0:34.21
497	TRUE	28	28	0	0:34.14
498	TRUE	28	28	0	0:34.30
499	TRUE	28	28	0	0:34.23
500	TRUE	28	28	0	0:34.32

Table 2: Model checking results corresponding to PBLMSTL statement 8

Id	Result	#total	#true	#false	Execution time (min:sec)
1	TRUE	28	28	0	0:34.34
2	TRUE	28	28	0	0:34.39
3	TRUE	28	28	0	0:34.22
4	TRUE	28	28	0	0:34.27
5	TRUE	28	28	0	0:34.31
6	TRUE	28	28	0	0:34.41
7	TRUE	28	28	0	0:34.38
8	TRUE	28	28	0	0:34.26
9	TRUE	28	28	0	0:34.22
10	TRUE	28	28	0	0:34.21
11	TRUE	28	28	0	0:34.29
12	TRUE	28	28	0	0:34.53
13	TRUE	28	28	0	0:34.36
14	TRUE	28	28	0	0:34.41
15	TRUE	28	28	0	0:34.67
16	TRUE	28	28	0	0:34.30
17	TRUE	28	28	0	0:34.34
18	TRUE	28	28	0	0:34.42
19	TRUE	28	28	0	0:34.34
20	TRUE	28	28	0	0:34.38
21	TRUE	28	28	0	0:34.31
22	TRUE	28	28	0	0:34.29
23	TRUE	28	28	0	0:34.41
24	TRUE	28	28	0	0:34.23
25	TRUE	28	28	0	0:34.40
26	TRUE	28	28	0	0:34.27
27	TRUE	28	28	0	0:34.45
28	TRUE	28	28	0	0:34.45

Id	Result	#total	#true	#false	Execution time (min:sec)
29	TRUE	28	28	0	0:34.45
30	TRUE	28	28	0	0:34.40
31	TRUE	28	28	0	0:34.39
32	TRUE	28	28	0	0:34.39
33	TRUE	28	28	0	0:34.22
34	TRUE	28	28	0	0:34.35
35	TRUE	28	28	0	0:34.38
36	TRUE	28	28	0	0:34.31
37	TRUE	28	28	0	0:34.38
38	TRUE	28	28	0	0:34.48
39	TRUE	28	28	0	0:34.34
40	TRUE	28	28	0	0:34.44
41	TRUE	28	28	0	0:34.27
42	TRUE	28	28	0	0:34.47
43	TRUE	28	28	0	0:34.39
44	TRUE	28	28	0	0:34.40
45	TRUE	28	28	0	0:34.45
46	TRUE	28	28	0	0:34.41
47	TRUE	28	28	0	0:34.38
48	TRUE	28	28	0	0:34.28
49	TRUE	28	28	0	0:34.26
50	TRUE	28	28	0	0:34.46
51	TRUE	28	28	0	0:34.47
52	TRUE	28	28	0	0:34.37
53	TRUE	28	28	0	0:34.34
54	TRUE	28	28	0	0:34.40
55	TRUE	28	28	0	0:34.32
56	TRUE	28	28	0	0:34.42
57	TRUE	28	28	0	0:34.36
58	TRUE	28	28	0	0:34.37
59	TRUE	28	28	0	0:34.36
60	TRUE	28	28	0	0:34.45
61	TRUE	28	28	0	0:34.26
62	TRUE	28	28	0	0:34.27
63	TRUE	28	28	0	0:34.44
64	TRUE	28	28	0	0:34.33
65	TRUE	28	28	0	0:34.25
66	TRUE	28	28	0	0:34.44
67	TRUE	28	28	0	0:34.42
68	TRUE	28	28	0	0:34.41
69	TRUE	28	28	0	0:34.35
70	TRUE	28	28	0	0:34.29
71	TRUE	28	28	0	0:34.23
72	TRUE	28	28	0	0:34.41
73	TRUE	28	28	0	0:34.26
74	TRUE	28	28	0	0:34.17
75	TRUE	28	28	0	0:34.35
76	TRUE	28	28	0	0:34.57

Id	Result	#total	#true	#false	Execution time (min:sec)
77	TRUE	28	28	0	0:34.31
78	TRUE	28	28	0	0:34.18
79	TRUE	28	28	0	0:34.27
80	TRUE	28	28	0	0:34.32
81	TRUE	28	28	0	0:34.29
82	TRUE	28	28	0	0:34.41
83	TRUE	28	28	0	0:34.33
84	TRUE	28	28	0	0:34.54
85	TRUE	28	28	0	0:34.38
86	TRUE	28	28	0	0:34.36
87	TRUE	28	28	0	0:34.37
88	TRUE	28	28	0	0:34.37
89	TRUE	28	28	0	0:34.32
90	TRUE	28	28	0	0:34.25
91	TRUE	28	28	0	0:34.31
92	TRUE	28	28	0	0:34.25
93	TRUE	28	28	0	0:34.29
94	TRUE	28	28	0	0:34.21
95	TRUE	28	28	0	0:34.35
96	TRUE	28	28	0	0:34.47
97	TRUE	28	28	0	0:34.25
98	TRUE	28	28	0	0:34.22
99	TRUE	28	28	0	0:34.26
100	TRUE	28	28	0	0:34.25
101	TRUE	28	28	0	0:34.34
102	TRUE	28	28	0	0:34.39
103	TRUE	28	28	0	0:34.41
104	TRUE	28	28	0	0:34.34
105	TRUE	28	28	0	0:34.42
106	TRUE	28	28	0	0:34.29
107	TRUE	28	28	0	0:34.22
108	TRUE	28	28	0	0:34.36
109	TRUE	28	28	0	0:34.37
110	TRUE	28	28	0	0:34.20
111	TRUE	28	28	0	0:34.09
112	TRUE	28	28	0	0:34.23
113	TRUE	28	28	0	0:34.11
114	TRUE	28	28	0	0:34.08
115	TRUE	28	28	0	0:34.16
116	TRUE	28	28	0	0:34.27
117	TRUE	28	28	0	0:34.16
118	TRUE	28	28	0	0:34.21
119	TRUE	28	28	0	0:34.18
120	TRUE	28	28	0	0:34.27
121	TRUE	28	28	0	0:34.15
122	TRUE	28	28	0	0:34.30
123	TRUE	28	28	0	0:34.26
124	TRUE	28	28	0	0:34.19

Id	Result	#total	#true	#false	Execution time (min:sec)
125	TRUE	28	28	0	0:34.26
126	TRUE	28	28	0	0:34.29
127	TRUE	28	28	0	0:34.28
128	TRUE	28	28	0	0:34.13
129	TRUE	28	28	0	0:34.29
130	TRUE	28	28	0	0:34.11
131	TRUE	28	28	0	0:34.24
132	TRUE	28	28	0	0:34.24
133	TRUE	28	28	0	0:34.23
134	TRUE	28	28	0	0:34.14
135	TRUE	28	28	0	0:34.37
136	TRUE	28	28	0	0:34.18
137	TRUE	28	28	0	0:34.35
138	TRUE	28	28	0	0:34.21
139	TRUE	28	28	0	0:34.15
140	TRUE	28	28	0	0:34.13
141	TRUE	28	28	0	0:34.54
142	TRUE	28	28	0	0:35.43
143	TRUE	28	28	0	0:35.51
144	TRUE	28	28	0	0:35.44
145	TRUE	28	28	0	0:35.36
146	TRUE	28	28	0	0:36.01
147	TRUE	28	28	0	0:37.82
148	TRUE	28	28	0	0:37.12
149	TRUE	28	28	0	0:36.55
150	TRUE	28	28	0	0:36.58
151	TRUE	28	28	0	0:36.55
152	TRUE	28	28	0	0:36.50
153	TRUE	28	28	0	0:36.52
154	TRUE	28	28	0	0:36.51
155	TRUE	28	28	0	0:36.63
156	TRUE	28	28	0	0:36.55
157	TRUE	28	28	0	0:36.61
158	TRUE	28	28	0	0:36.75
159	TRUE	28	28	0	0:36.71
160	TRUE	28	28	0	0:36.60
161	TRUE	28	28	0	0:36.59
162	TRUE	28	28	0	0:36.75
163	TRUE	28	28	0	0:36.62
164	TRUE	28	28	0	0:36.54
165	TRUE	28	28	0	0:36.49
166	TRUE	28	28	0	0:36.55
167	TRUE	28	28	0	0:36.82
168	TRUE	28	28	0	0:36.62
169	TRUE	28	28	0	0:36.38
170	TRUE	28	28	0	0:36.56
171	TRUE	28	28	0	0:36.47
172	TRUE	28	28	0	0:36.69

Id	Result	#total	#true	#false	Execution time (min:sec)
173	TRUE	28	28	0	0:36.49
174	TRUE	28	28	0	0:36.45
175	TRUE	28	28	0	0:36.54
176	TRUE	28	28	0	0:36.47
177	TRUE	28	28	0	0:36.63
178	TRUE	28	28	0	0:36.55
179	TRUE	28	28	0	0:36.51
180	TRUE	28	28	0	0:36.34
181	TRUE	28	28	0	0:37.21
182	TRUE	28	28	0	0:37.25
183	TRUE	28	28	0	0:37.69
184	TRUE	28	28	0	0:37.38
185	TRUE	28	28	0	0:37.53
186	TRUE	28	28	0	0:37.68
187	TRUE	28	28	0	0:37.62
188	TRUE	28	28	0	0:37.38
189	TRUE	28	28	0	0:37.79
190	TRUE	28	28	0	0:37.55
191	TRUE	28	28	0	0:37.32
192	TRUE	28	28	0	0:37.40
193	TRUE	28	28	0	0:37.53
194	TRUE	28	28	0	0:38.29
195	TRUE	28	28	0	0:37.46
196	TRUE	28	28	0	0:37.69
197	TRUE	28	28	0	0:37.74
198	TRUE	28	28	0	0:38.36
199	TRUE	28	28	0	0:37.54
200	TRUE	28	28	0	0:37.61
201	TRUE	28	28	0	0:37.48
202	TRUE	28	28	0	0:37.68
203	TRUE	28	28	0	0:37.11
204	TRUE	28	28	0	0:37.86
205	TRUE	28	28	0	0:37.29
206	TRUE	28	28	0	0:37.64
207	TRUE	28	28	0	0:37.46
208	TRUE	28	28	0	0:35.43
209	TRUE	28	28	0	0:35.76
210	TRUE	28	28	0	0:35.62
211	TRUE	28	28	0	0:35.55
212	TRUE	28	28	0	0:35.53
213	TRUE	28	28	0	0:35.77
214	TRUE	28	28	0	0:35.74
215	TRUE	28	28	0	0:35.68
216	TRUE	28	28	0	0:35.87
217	TRUE	28	28	0	0:35.72
218	TRUE	28	28	0	0:35.67
219	TRUE	28	28	0	0:35.55
220	TRUE	28	28	0	0:35.71

Id	Result	#total	#true	#false	Execution time (min:sec)
221	TRUE	28	28	0	0:35.85
222	TRUE	28	28	0	0:35.76
223	TRUE	28	28	0	0:35.86
224	TRUE	28	28	0	0:35.82
225	TRUE	28	28	0	0:35.86
226	TRUE	28	28	0	0:35.70
227	TRUE	28	28	0	0:35.80
228	TRUE	28	28	0	0:35.53
229	TRUE	28	28	0	0:35.95
230	TRUE	28	28	0	0:35.89
231	TRUE	28	28	0	0:35.83
232	TRUE	28	28	0	0:35.71
233	TRUE	28	28	0	0:35.84
234	TRUE	28	28	0	0:35.60
235	TRUE	28	28	0	0:35.65
236	TRUE	28	28	0	0:35.83
237	TRUE	28	28	0	0:35.75
238	TRUE	28	28	0	0:35.93
239	TRUE	28	28	0	0:35.98
240	TRUE	28	28	0	0:36.01
241	TRUE	28	28	0	0:35.55
242	TRUE	28	28	0	0:35.90
243	TRUE	28	28	0	0:36.00
244	TRUE	28	28	0	0:36.30
245	TRUE	28	28	0	0:35.83
246	TRUE	28	28	0	0:35.69
247	TRUE	28	28	0	0:36.05
248	TRUE	28	28	0	0:35.68
249	TRUE	28	28	0	0:35.96
250	TRUE	28	28	0	0:35.76
251	TRUE	28	28	0	0:35.75
252	TRUE	28	28	0	0:35.89
253	TRUE	28	28	0	0:35.63
254	TRUE	28	28	0	0:35.76
255	TRUE	28	28	0	0:35.57
256	TRUE	28	28	0	0:35.77
257	TRUE	28	28	0	0:35.75
258	TRUE	28	28	0	0:36.13
259	TRUE	28	28	0	0:35.74
260	TRUE	28	28	0	0:35.67
261	TRUE	28	28	0	0:35.93
262	TRUE	28	28	0	0:35.95
263	TRUE	28	28	0	0:35.73
264	TRUE	28	28	0	0:35.77
265	TRUE	28	28	0	0:35.94
266	TRUE	28	28	0	0:35.49
267	TRUE	28	28	0	0:35.52
268	TRUE	28	28	0	0:35.64

Id	Result	#total	#true	#false	Execution time (min:sec)
269	TRUE	28	28	0	0:35.61
270	TRUE	28	28	0	0:36.25
271	TRUE	28	28	0	0:35.54
272	TRUE	28	28	0	0:36.06
273	TRUE	28	28	0	0:35.85
274	TRUE	28	28	0	0:35.83
275	TRUE	28	28	0	0:35.78
276	TRUE	28	28	0	0:35.81
277	TRUE	28	28	0	0:35.94
278	TRUE	28	28	0	0:35.77
279	TRUE	28	28	0	0:35.71
280	TRUE	28	28	0	0:35.86
281	TRUE	28	28	0	0:35.79
282	TRUE	28	28	0	0:35.80
283	TRUE	28	28	0	0:35.74
284	TRUE	28	28	0	0:35.86
285	TRUE	28	28	0	0:35.68
286	TRUE	28	28	0	0:35.78
287	TRUE	28	28	0	0:35.93
288	TRUE	28	28	0	0:35.85
289	TRUE	28	28	0	0:35.79
290	TRUE	28	28	0	0:35.83
291	TRUE	28	28	0	0:35.64
292	TRUE	28	28	0	0:35.87
293	TRUE	28	28	0	0:35.73
294	TRUE	28	28	0	0:35.76
295	TRUE	28	28	0	0:35.66
296	TRUE	28	28	0	0:35.81
297	TRUE	28	28	0	0:35.88
298	TRUE	28	28	0	0:35.50
299	TRUE	28	28	0	0:36.10
300	TRUE	28	28	0	0:35.70
301	TRUE	28	28	0	0:35.68
302	TRUE	28	28	0	0:35.61
303	TRUE	28	28	0	0:35.81
304	TRUE	28	28	0	0:35.54
305	TRUE	28	28	0	0:35.58
306	TRUE	28	28	0	0:35.47
307	TRUE	28	28	0	0:35.78
308	TRUE	28	28	0	0:35.75
309	TRUE	28	28	0	0:35.57
310	TRUE	28	28	0	0:35.69
311	TRUE	28	28	0	0:35.87
312	TRUE	28	28	0	0:35.58
313	TRUE	28	28	0	0:35.89
314	TRUE	28	28	0	0:35.59
315	TRUE	28	28	0	0:35.91
316	TRUE	28	28	0	0:35.69

Id	Result	#total	#true	#false	Execution time (min:sec)
317	TRUE	28	28	0	0:35.79
318	TRUE	28	28	0	0:35.73
319	TRUE	28	28	0	0:35.52
320	TRUE	28	28	0	0:35.72
321	TRUE	28	28	0	0:35.61
322	TRUE	28	28	0	0:35.64
323	TRUE	28	28	0	0:35.55
324	TRUE	28	28	0	0:35.90
325	TRUE	28	28	0	0:35.74
326	TRUE	28	28	0	0:35.73
327	TRUE	28	28	0	0:35.77
328	TRUE	28	28	0	0:35.58
329	TRUE	28	28	0	0:35.77
330	TRUE	28	28	0	0:35.56
331	TRUE	28	28	0	0:35.78
332	TRUE	28	28	0	0:35.60
333	TRUE	28	28	0	0:35.60
334	TRUE	28	28	0	0:35.80
335	TRUE	28	28	0	0:35.81
336	TRUE	28	28	0	0:35.75
337	TRUE	28	28	0	0:35.50
338	TRUE	28	28	0	0:35.87
339	TRUE	28	28	0	0:35.52
340	TRUE	28	28	0	0:35.57
341	TRUE	28	28	0	0:35.78
342	TRUE	28	28	0	0:35.67
343	TRUE	28	28	0	0:35.72
344	TRUE	28	28	0	0:35.67
345	TRUE	28	28	0	0:35.95
346	TRUE	28	28	0	0:35.57
347	TRUE	28	28	0	0:35.70
348	TRUE	28	28	0	0:35.75
349	TRUE	28	28	0	0:35.68
350	TRUE	28	28	0	0:35.52
351	TRUE	28	28	0	0:35.79
352	TRUE	28	28	0	0:35.75
353	TRUE	28	28	0	0:35.62
354	TRUE	28	28	0	0:35.68
355	TRUE	28	28	0	0:35.82
356	TRUE	28	28	0	0:35.71
357	TRUE	28	28	0	0:35.51
358	TRUE	28	28	0	0:35.47
359	TRUE	28	28	0	0:35.95
360	TRUE	28	28	0	0:35.64
361	TRUE	28	28	0	0:35.70
362	TRUE	28	28	0	0:35.65
363	TRUE	28	28	0	0:35.73
364	TRUE	28	28	0	0:35.71

Id	Result	#total	#true	#false	Execution time (min:sec)
365	TRUE	28	28	0	0:35.81
366	TRUE	28	28	0	0:35.90
367	TRUE	28	28	0	0:35.49
368	TRUE	28	28	0	0:35.57
369	TRUE	28	28	0	0:35.65
370	TRUE	28	28	0	0:36.27
371	TRUE	28	28	0	0:35.87
372	TRUE	28	28	0	0:35.66
373	TRUE	28	28	0	0:35.77
374	TRUE	28	28	0	0:36.11
375	TRUE	28	28	0	0:35.62
376	TRUE	28	28	0	0:35.47
377	TRUE	28	28	0	0:35.93
378	TRUE	28	28	0	0:35.44
379	TRUE	28	28	0	0:35.86
380	TRUE	28	28	0	0:35.80
381	TRUE	28	28	0	0:35.76
382	TRUE	28	28	0	0:35.92
383	TRUE	28	28	0	0:35.90
384	TRUE	28	28	0	0:35.64
385	TRUE	28	28	0	0:35.80
386	TRUE	28	28	0	0:35.71
387	TRUE	28	28	0	0:35.71
388	TRUE	28	28	0	0:35.66
389	TRUE	28	28	0	0:35.79
390	TRUE	28	28	0	0:35.91
391	TRUE	28	28	0	0:35.89
392	TRUE	28	28	0	0:35.53
393	TRUE	28	28	0	0:35.57
394	TRUE	28	28	0	0:35.78
395	TRUE	28	28	0	0:35.52
396	TRUE	28	28	0	0:35.65
397	TRUE	28	28	0	0:35.70
398	TRUE	28	28	0	0:35.93
399	TRUE	28	28	0	0:35.63
400	TRUE	28	28	0	0:35.86
401	TRUE	28	28	0	0:35.65
402	TRUE	28	28	0	0:35.82
403	TRUE	28	28	0	0:35.65
404	TRUE	28	28	0	0:35.76
405	TRUE	28	28	0	0:35.68
406	TRUE	28	28	0	0:35.58
407	TRUE	28	28	0	0:35.75
408	TRUE	28	28	0	0:35.47
409	TRUE	28	28	0	0:35.65
410	TRUE	28	28	0	0:35.61
411	TRUE	28	28	0	0:35.73
412	TRUE	28	28	0	0:35.56

Id	Result	#total	#true	#false	Execution time (min:sec)
413	TRUE	28	28	0	0:35.64
414	TRUE	28	28	0	0:35.50
415	TRUE	28	28	0	0:35.63
416	TRUE	28	28	0	0:35.69
417	TRUE	28	28	0	0:35.75
418	TRUE	28	28	0	0:35.55
419	TRUE	28	28	0	0:35.56
420	TRUE	28	28	0	0:35.35
421	TRUE	28	28	0	0:35.58
422	TRUE	28	28	0	0:35.84
423	TRUE	28	28	0	0:35.57
424	TRUE	28	28	0	0:35.64
425	TRUE	28	28	0	0:35.57
426	TRUE	28	28	0	0:35.67
427	TRUE	28	28	0	0:35.50
428	TRUE	28	28	0	0:35.51
429	TRUE	28	28	0	0:35.54
430	TRUE	28	28	0	0:35.54
431	TRUE	28	28	0	0:35.54
432	TRUE	28	28	0	0:35.35
433	TRUE	28	28	0	0:35.61
434	TRUE	28	28	0	0:35.63
435	TRUE	28	28	0	0:35.48
436	TRUE	28	28	0	0:35.52
437	TRUE	28	28	0	0:35.48
438	TRUE	28	28	0	0:35.69
439	TRUE	28	28	0	0:35.63
440	TRUE	28	28	0	0:35.52
441	TRUE	28	28	0	0:35.59
442	TRUE	28	28	0	0:35.52
443	TRUE	28	28	0	0:35.60
444	TRUE	28	28	0	0:35.64
445	TRUE	28	28	0	0:35.57
446	TRUE	28	28	0	0:35.73
447	TRUE	28	28	0	0:35.66
448	TRUE	28	28	0	0:35.53
449	TRUE	28	28	0	0:35.60
450	TRUE	28	28	0	0:35.64
451	TRUE	28	28	0	0:35.44
452	TRUE	28	28	0	0:35.44
453	TRUE	28	28	0	0:35.48
454	TRUE	28	28	0	0:35.56
455	TRUE	28	28	0	0:35.53
456	TRUE	28	28	0	0:35.76
457	TRUE	28	28	0	0:35.52
458	TRUE	28	28	0	0:35.59
459	TRUE	28	28	0	0:35.54
460	TRUE	28	28	0	0:35.55

Id	Result	#total	#true	#false	Execution time (min:sec)
461	TRUE	28	28	0	0:35.56
462	TRUE	28	28	0	0:35.44
463	TRUE	28	28	0	0:35.49
464	TRUE	28	28	0	0:35.51
465	TRUE	28	28	0	0:35.54
466	TRUE	28	28	0	0:35.48
467	TRUE	28	28	0	0:35.68
468	TRUE	28	28	0	0:35.29
469	TRUE	28	28	0	0:35.51
470	TRUE	28	28	0	0:35.74
471	TRUE	28	28	0	0:35.46
472	TRUE	28	28	0	0:35.56
473	TRUE	28	28	0	0:35.46
474	TRUE	28	28	0	0:35.54
475	TRUE	28	28	0	0:35.56
476	TRUE	28	28	0	0:35.58
477	TRUE	28	28	0	0:35.66
478	TRUE	28	28	0	0:35.43
479	TRUE	28	28	0	0:35.45
480	TRUE	28	28	0	0:35.55
481	TRUE	28	28	0	0:35.46
482	TRUE	28	28	0	0:35.45
483	TRUE	28	28	0	0:35.38
484	TRUE	28	28	0	0:35.49
485	TRUE	28	28	0	0:35.61
486	TRUE	28	28	0	0:35.34
487	TRUE	28	28	0	0:35.47
488	TRUE	28	28	0	0:35.54
489	TRUE	28	28	0	0:35.68
490	TRUE	28	28	0	0:35.60
491	TRUE	28	28	0	0:35.64
492	TRUE	28	28	0	0:35.44
493	TRUE	28	28	0	0:35.59
494	TRUE	28	28	0	0:35.92
495	TRUE	28	28	0	0:35.87
496	TRUE	28	28	0	0:35.49
497	TRUE	28	28	0	0:35.54
498	TRUE	28	28	0	0:35.50
499	TRUE	28	28	0	0:35.43
500	TRUE	28	28	0	0:35.49

Table 3: Model checking results corresponding to PBLMSTL statement 9

Id	Result	#total	#true	#false	Execution time (min:sec)
1	TRUE	28	28	0	0:35.32

Id	Result	#total	#true	#false	Execution time (min:sec)
2	TRUE	28	28	0	0:35.39
3	TRUE	28	28	0	0:35.51
4	TRUE	28	28	0	0:35.53
5	TRUE	28	28	0	0:35.55
6	TRUE	28	28	0	0:35.75
7	TRUE	28	28	0	0:35.31
8	TRUE	28	28	0	0:35.48
9	TRUE	28	28	0	0:35.62
10	TRUE	28	28	0	0:35.51
11	TRUE	28	28	0	0:35.50
12	TRUE	28	28	0	0:35.44
13	TRUE	28	28	0	0:35.31
14	TRUE	28	28	0	0:35.50
15	TRUE	28	28	0	0:35.46
16	TRUE	28	28	0	0:35.54
17	TRUE	28	28	0	0:35.48
18	TRUE	28	28	0	0:35.38
19	TRUE	28	28	0	0:35.59
20	TRUE	28	28	0	0:35.57
21	TRUE	28	28	0	0:35.50
22	TRUE	28	28	0	0:35.78
23	TRUE	28	28	0	0:36.28
24	TRUE	28	28	0	0:35.63
25	TRUE	28	28	0	0:36.07
26	TRUE	28	28	0	0:35.59
27	TRUE	28	28	0	0:35.50
28	TRUE	28	28	0	0:35.64
29	TRUE	28	28	0	0:35.53
30	TRUE	28	28	0	0:35.38
31	TRUE	28	28	0	0:35.41
32	TRUE	28	28	0	0:35.35
33	TRUE	28	28	0	0:35.89
34	TRUE	28	28	0	0:35.53
35	TRUE	28	28	0	0:35.79
36	TRUE	28	28	0	0:35.39
37	TRUE	28	28	0	0:35.48
38	TRUE	28	28	0	0:35.64
39	TRUE	28	28	0	0:35.46
40	TRUE	28	28	0	0:35.48
41	TRUE	28	28	0	0:35.49
42	TRUE	28	28	0	0:35.50
43	TRUE	28	28	0	0:35.65
44	TRUE	28	28	0	0:35.50
45	TRUE	28	28	0	0:35.47
46	TRUE	28	28	0	0:35.36
47	TRUE	28	28	0	0:35.46
48	TRUE	28	28	0	0:35.43
49	TRUE	28	28	0	0:35.63

Id	Result	#total	#true	#false	Execution time (min:sec)
50	TRUE	28	28	0	0:35.44
51	TRUE	28	28	0	0:35.55
52	TRUE	28	28	0	0:35.39
53	TRUE	28	28	0	0:35.42
54	TRUE	28	28	0	0:35.40
55	TRUE	28	28	0	0:35.55
56	TRUE	28	28	0	0:35.44
57	TRUE	28	28	0	0:35.41
58	TRUE	28	28	0	0:35.46
59	TRUE	28	28	0	0:35.50
60	TRUE	28	28	0	0:35.53
61	TRUE	28	28	0	0:35.51
62	TRUE	28	28	0	0:35.29
63	TRUE	28	28	0	0:35.32
64	TRUE	28	28	0	0:35.53
65	TRUE	28	28	0	0:35.44
66	TRUE	28	28	0	0:35.42
67	TRUE	28	28	0	0:35.36
68	TRUE	28	28	0	0:35.40
69	TRUE	28	28	0	0:35.46
70	TRUE	28	28	0	0:35.47
71	TRUE	28	28	0	0:35.44
72	TRUE	28	28	0	0:35.45
73	TRUE	28	28	0	0:35.52
74	TRUE	28	28	0	0:35.55
75	TRUE	28	28	0	0:35.33
76	TRUE	28	28	0	0:35.46
77	TRUE	28	28	0	0:35.25
78	TRUE	28	28	0	0:35.63
79	TRUE	28	28	0	0:35.21
80	TRUE	28	28	0	0:35.49
81	TRUE	28	28	0	0:35.20
82	TRUE	28	28	0	0:36.01
83	TRUE	28	28	0	0:35.60
84	TRUE	28	28	0	0:35.45
85	TRUE	28	28	0	0:35.55
86	TRUE	28	28	0	0:35.48
87	TRUE	28	28	0	0:35.43
88	TRUE	28	28	0	0:35.51
89	TRUE	28	28	0	0:35.81
90	TRUE	28	28	0	0:35.64
91	TRUE	28	28	0	0:35.49
92	TRUE	28	28	0	0:35.64
93	TRUE	28	28	0	0:35.63
94	TRUE	28	28	0	0:35.46
95	TRUE	28	28	0	0:35.47
96	TRUE	28	28	0	0:35.19
97	TRUE	28	28	0	0:35.24

Id	Result	#total	#true	#false	Execution time (min:sec)
98	TRUE	28	28	0	0:36.01
99	TRUE	28	28	0	0:36.60
100	TRUE	28	28	0	0:36.97
101	TRUE	28	28	0	0:40.44
102	TRUE	28	28	0	0:39.23
103	TRUE	28	28	0	0:37.37
104	TRUE	28	28	0	0:36.57
105	TRUE	28	28	0	0:36.55
106	TRUE	28	28	0	0:36.59
107	TRUE	28	28	0	0:36.34
108	TRUE	28	28	0	0:36.58
109	TRUE	28	28	0	0:36.48
110	TRUE	28	28	0	0:36.72
111	TRUE	28	28	0	0:36.40
112	TRUE	28	28	0	0:36.83
113	TRUE	28	28	0	0:36.33
114	TRUE	28	28	0	0:36.27
115	TRUE	28	28	0	0:36.40
116	TRUE	28	28	0	0:36.42
117	TRUE	28	28	0	0:36.26
118	TRUE	28	28	0	0:36.76
119	TRUE	28	28	0	0:36.46
120	TRUE	28	28	0	0:36.36
121	TRUE	28	28	0	0:36.48
122	TRUE	28	28	0	0:36.37
123	TRUE	28	28	0	0:36.51
124	TRUE	28	28	0	0:36.45
125	TRUE	28	28	0	0:36.61
126	TRUE	28	28	0	0:36.27
127	TRUE	28	28	0	0:36.46
128	TRUE	28	28	0	0:36.31
129	TRUE	28	28	0	0:36.27
130	TRUE	28	28	0	0:36.26
131	TRUE	28	28	0	0:36.39
132	TRUE	28	28	0	0:36.24
133	TRUE	28	28	0	0:36.31
134	TRUE	28	28	0	0:36.20
135	TRUE	28	28	0	0:36.28
136	TRUE	28	28	0	0:36.48
137	TRUE	28	28	0	0:36.21
138	TRUE	28	28	0	0:36.49
139	TRUE	28	28	0	0:36.28
140	TRUE	28	28	0	0:36.41
141	TRUE	28	28	0	0:36.33
142	TRUE	28	28	0	0:36.46
143	TRUE	28	28	0	0:36.29
144	TRUE	28	28	0	0:36.32
145	TRUE	28	28	0	0:36.26

Id	Result	#total	#true	#false	Execution time (min:sec)
146	TRUE	28	28	0	0:36.29
147	TRUE	28	28	0	0:36.19
148	TRUE	28	28	0	0:36.28
149	TRUE	28	28	0	0:36.33
150	TRUE	28	28	0	0:39.04
151	TRUE	28	28	0	0:39.25
152	TRUE	28	28	0	0:37.53
153	TRUE	28	28	0	0:38.76
154	TRUE	28	28	0	0:38.40
155	TRUE	28	28	0	0:38.39
156	TRUE	28	28	0	0:38.55
157	TRUE	28	28	0	0:38.31
158	TRUE	28	28	0	0:38.14
159	TRUE	28	28	0	0:38.97
160	TRUE	28	28	0	0:38.40
161	TRUE	28	28	0	0:38.82
162	TRUE	28	28	0	0:38.57
163	TRUE	28	28	0	0:38.98
164	TRUE	28	28	0	0:39.34
165	TRUE	28	28	0	0:39.03
166	TRUE	28	28	0	0:39.14
167	TRUE	28	28	0	0:38.83
168	TRUE	28	28	0	0:38.66
169	TRUE	28	28	0	0:39.10
170	TRUE	28	28	0	0:39.04
171	TRUE	28	28	0	0:38.60
172	TRUE	28	28	0	0:38.56
173	TRUE	28	28	0	0:38.52
174	TRUE	28	28	0	0:38.85
175	TRUE	28	28	0	0:38.70
176	TRUE	28	28	0	0:39.28
177	TRUE	28	28	0	0:39.13
178	TRUE	28	28	0	0:39.06
179	TRUE	28	28	0	0:38.72
180	TRUE	28	28	0	0:39.48
181	TRUE	28	28	0	0:38.89
182	TRUE	28	28	0	0:39.25
183	TRUE	28	28	0	0:40.03
184	TRUE	28	28	0	0:39.56
185	TRUE	28	28	0	0:39.00
186	TRUE	28	28	0	0:39.07
187	TRUE	28	28	0	0:38.54
188	TRUE	28	28	0	0:38.97
189	TRUE	28	28	0	0:39.52
190	TRUE	28	28	0	0:38.63
191	TRUE	28	28	0	0:38.41
192	TRUE	28	28	0	0:39.20
193	TRUE	28	28	0	0:38.97

Id	Result	#total	#true	#false	Execution time (min:sec)
194	TRUE	28	28	0	0:39.06
195	TRUE	28	28	0	0:38.50
196	TRUE	28	28	0	0:38.69
197	TRUE	28	28	0	0:39.26
198	TRUE	28	28	0	0:38.76
199	TRUE	28	28	0	0:38.57
200	TRUE	28	28	0	0:38.88
201	TRUE	28	28	0	0:38.54
202	TRUE	28	28	0	0:38.47
203	TRUE	28	28	0	0:38.72
204	TRUE	28	28	0	0:41.09
205	TRUE	28	28	0	0:38.92
206	TRUE	28	28	0	0:38.69
207	TRUE	28	28	0	0:38.65
208	TRUE	28	28	0	0:38.81
209	TRUE	28	28	0	0:39.07
210	TRUE	28	28	0	0:39.02
211	TRUE	28	28	0	0:39.19
212	TRUE	28	28	0	0:40.03
213	TRUE	28	28	0	0:39.29
214	TRUE	28	28	0	0:39.02
215	TRUE	28	28	0	0:38.83
216	TRUE	28	28	0	0:38.57
217	TRUE	28	28	0	0:39.04
218	TRUE	28	28	0	0:38.89
219	TRUE	28	28	0	0:38.86
220	TRUE	28	28	0	0:38.74
221	TRUE	28	28	0	0:39.04
222	TRUE	28	28	0	0:39.03
223	TRUE	28	28	0	0:38.89
224	TRUE	28	28	0	0:38.93
225	TRUE	28	28	0	0:38.89
226	TRUE	28	28	0	0:39.13
227	TRUE	28	28	0	0:38.92
228	TRUE	28	28	0	0:38.45
229	TRUE	28	28	0	0:39.10
230	TRUE	28	28	0	0:39.61
231	TRUE	28	28	0	0:39.00
232	TRUE	28	28	0	0:38.53
233	TRUE	28	28	0	0:38.18
234	TRUE	28	28	0	0:39.03
235	TRUE	28	28	0	0:39.54
236	TRUE	28	28	0	0:39.21
237	TRUE	28	28	0	0:38.63
238	TRUE	28	28	0	0:39.42
239	TRUE	28	28	0	0:38.94
240	TRUE	28	28	0	0:39.06
241	TRUE	28	28	0	0:38.97

Id	Result	#total	#true	#false	Execution time (min:sec)
242	TRUE	28	28	0	0:39.21
243	TRUE	28	28	0	0:38.86
244	TRUE	28	28	0	0:39.37
245	TRUE	28	28	0	0:39.28
246	TRUE	28	28	0	0:38.91
247	TRUE	28	28	0	0:39.26
248	TRUE	28	28	0	0:39.11
249	TRUE	28	28	0	0:39.13
250	TRUE	28	28	0	0:38.44
251	TRUE	28	28	0	0:38.52
252	TRUE	28	28	0	0:39.13
253	TRUE	28	28	0	0:39.10
254	TRUE	28	28	0	0:38.91
255	TRUE	28	28	0	0:38.72
256	TRUE	28	28	0	0:39.04
257	TRUE	28	28	0	0:39.07
258	TRUE	28	28	0	0:38.62
259	TRUE	28	28	0	0:38.86
260	TRUE	28	28	0	0:39.47
261	TRUE	28	28	0	0:39.49
262	TRUE	28	28	0	0:39.12
263	TRUE	28	28	0	0:39.33
264	TRUE	28	28	0	0:38.81
265	TRUE	28	28	0	0:38.98
266	TRUE	28	28	0	0:39.02
267	TRUE	28	28	0	0:38.83
268	TRUE	28	28	0	0:39.08
269	TRUE	28	28	0	0:39.62
270	TRUE	28	28	0	0:39.04
271	TRUE	28	28	0	0:39.24
272	TRUE	28	28	0	0:39.10
273	TRUE	28	28	0	0:39.34
274	TRUE	28	28	0	0:39.24
275	TRUE	28	28	0	0:38.87
276	TRUE	28	28	0	0:38.74
277	TRUE	28	28	0	0:38.44
278	TRUE	28	28	0	0:39.01
279	TRUE	28	28	0	0:39.02
280	TRUE	28	28	0	0:39.48
281	TRUE	28	28	0	0:38.96
282	TRUE	28	28	0	0:38.78
283	TRUE	28	28	0	0:39.02
284	TRUE	28	28	0	0:38.97
285	TRUE	28	28	0	0:38.34
286	TRUE	28	28	0	0:39.22
287	TRUE	28	28	0	0:38.88
288	TRUE	28	28	0	0:38.81
289	TRUE	28	28	0	0:39.01

Id	Result	#total	#true	#false	Execution time (min:sec)
290	TRUE	28	28	0	0:38.99
291	TRUE	28	28	0	0:38.91
292	TRUE	28	28	0	0:39.44
293	TRUE	28	28	0	0:39.19
294	TRUE	28	28	0	0:39.29
295	TRUE	28	28	0	0:38.57
296	TRUE	28	28	0	0:38.57
297	TRUE	28	28	0	0:39.30
298	TRUE	28	28	0	0:39.37
299	TRUE	28	28	0	0:38.85
300	TRUE	28	28	0	0:38.94
301	TRUE	28	28	0	0:38.92
302	TRUE	28	28	0	0:39.06
303	TRUE	28	28	0	0:39.15
304	TRUE	28	28	0	0:39.20
305	TRUE	28	28	0	0:38.63
306	TRUE	28	28	0	0:38.75
307	TRUE	28	28	0	0:39.32
308	TRUE	28	28	0	0:38.62
309	TRUE	28	28	0	0:38.60
310	TRUE	28	28	0	0:39.00
311	TRUE	28	28	0	0:39.40
312	TRUE	28	28	0	0:39.47
313	TRUE	28	28	0	0:38.92
314	TRUE	28	28	0	0:38.49
315	TRUE	28	28	0	0:38.88
316	TRUE	28	28	0	0:39.40
317	TRUE	28	28	0	0:39.09
318	TRUE	28	28	0	0:38.72
319	TRUE	28	28	0	0:38.57
320	TRUE	28	28	0	0:39.15
321	TRUE	28	28	0	0:39.00
322	TRUE	28	28	0	0:38.76
323	TRUE	28	28	0	0:38.83
324	TRUE	28	28	0	0:39.23
325	TRUE	28	28	0	0:39.32
326	TRUE	28	28	0	0:39.17
327	TRUE	28	28	0	0:38.82
328	TRUE	28	28	0	0:43.61
329	TRUE	28	28	0	0:46.31
330	TRUE	28	28	0	0:37.62
331	TRUE	28	28	0	0:38.58
332	TRUE	28	28	0	0:42.60
333	TRUE	28	28	0	0:37.73
334	TRUE	28	28	0	0:35.48
335	TRUE	28	28	0	0:35.45
336	TRUE	28	28	0	0:36.21
337	TRUE	28	28	0	0:35.94

Id	Result	#total	#true	#false	Execution time (min:sec)
338	TRUE	28	28	0	0:35.41
339	TRUE	28	28	0	0:35.85
340	TRUE	28	28	0	0:34.59
341	TRUE	28	28	0	0:34.55
342	TRUE	28	28	0	0:34.56
343	TRUE	28	28	0	0:34.60
344	TRUE	28	28	0	0:34.56
345	TRUE	28	28	0	0:34.57
346	TRUE	28	28	0	0:34.47
347	TRUE	28	28	0	0:34.46
348	TRUE	28	28	0	0:34.43
349	TRUE	28	28	0	0:34.43
350	TRUE	28	28	0	0:34.45
351	TRUE	28	28	0	0:34.63
352	TRUE	28	28	0	0:34.47
353	TRUE	28	28	0	0:34.43
354	TRUE	28	28	0	0:34.51
355	TRUE	28	28	0	0:34.57
356	TRUE	28	28	0	0:34.54
357	TRUE	28	28	0	0:34.31
358	TRUE	28	28	0	0:34.53
359	TRUE	28	28	0	0:34.82
360	TRUE	28	28	0	0:34.50
361	TRUE	28	28	0	0:34.44
362	TRUE	28	28	0	0:34.42
363	TRUE	28	28	0	0:34.46
364	TRUE	28	28	0	0:34.32
365	TRUE	28	28	0	0:34.42
366	TRUE	28	28	0	0:34.40
367	TRUE	28	28	0	0:34.43
368	TRUE	28	28	0	0:34.48
369	TRUE	28	28	0	0:34.39
370	TRUE	28	28	0	0:34.28
371	TRUE	28	28	0	0:34.62
372	TRUE	28	28	0	0:34.34
373	TRUE	28	28	0	0:34.33
374	TRUE	28	28	0	0:34.28
375	TRUE	28	28	0	0:34.46
376	TRUE	28	28	0	0:34.62
377	TRUE	28	28	0	0:34.40
378	TRUE	28	28	0	0:34.54
379	TRUE	28	28	0	0:34.41
380	TRUE	28	28	0	0:34.36
381	TRUE	28	28	0	0:34.35
382	TRUE	28	28	0	0:34.50
383	TRUE	28	28	0	0:34.48
384	TRUE	28	28	0	0:34.19
385	TRUE	28	28	0	0:34.49

Id	Result	#total	#true	#false	Execution time (min:sec)
386	TRUE	28	28	0	0:34.32
387	TRUE	28	28	0	0:34.35
388	TRUE	28	28	0	0:34.30
389	TRUE	28	28	0	0:34.40
390	TRUE	28	28	0	0:34.38
391	TRUE	28	28	0	0:34.27
392	TRUE	28	28	0	0:34.33
393	TRUE	28	28	0	0:34.17
394	TRUE	28	28	0	0:34.53
395	TRUE	28	28	0	0:34.27
396	TRUE	28	28	0	0:34.19
397	TRUE	28	28	0	0:34.61
398	TRUE	28	28	0	0:34.43
399	TRUE	28	28	0	0:34.20
400	TRUE	28	28	0	0:34.29
401	TRUE	28	28	0	0:34.30
402	TRUE	28	28	0	0:34.45
403	TRUE	28	28	0	0:34.36
404	TRUE	28	28	0	0:34.40
405	TRUE	28	28	0	0:34.40
406	TRUE	28	28	0	0:34.18
407	TRUE	28	28	0	0:34.30
408	TRUE	28	28	0	0:34.32
409	TRUE	28	28	0	0:34.38
410	TRUE	28	28	0	0:34.34
411	TRUE	28	28	0	0:34.29
412	TRUE	28	28	0	0:34.19
413	TRUE	28	28	0	0:34.34
414	TRUE	28	28	0	0:34.23
415	TRUE	28	28	0	0:34.17
416	TRUE	28	28	0	0:34.25
417	TRUE	28	28	0	0:34.23
418	TRUE	28	28	0	0:34.45
419	TRUE	28	28	0	0:34.21
420	TRUE	28	28	0	0:34.39
421	TRUE	28	28	0	0:34.32
422	TRUE	28	28	0	0:34.16
423	TRUE	28	28	0	0:34.51
424	TRUE	28	28	0	0:34.24
425	TRUE	28	28	0	0:34.35
426	TRUE	28	28	0	0:34.30
427	TRUE	28	28	0	0:34.32
428	TRUE	28	28	0	0:34.32
429	TRUE	28	28	0	0:34.26
430	TRUE	28	28	0	0:34.32
431	TRUE	28	28	0	0:34.46
432	TRUE	28	28	0	0:34.40
433	TRUE	28	28	0	0:34.65

Id	Result	#total	#true	#false	Execution time (min:sec)
434	TRUE	28	28	0	0:34.52
435	TRUE	28	28	0	0:34.42
436	TRUE	28	28	0	0:34.45
437	TRUE	28	28	0	0:34.52
438	TRUE	28	28	0	0:34.60
439	TRUE	28	28	0	0:34.71
440	TRUE	28	28	0	0:34.59
441	TRUE	28	28	0	0:34.45
442	TRUE	28	28	0	0:34.31
443	TRUE	28	28	0	0:34.36
444	TRUE	28	28	0	0:34.29
445	TRUE	28	28	0	0:34.41
446	TRUE	28	28	0	0:34.42
447	TRUE	28	28	0	0:34.18
448	TRUE	28	28	0	0:34.49
449	TRUE	28	28	0	0:34.50
450	TRUE	28	28	0	0:34.28
451	TRUE	28	28	0	0:34.30
452	TRUE	28	28	0	0:34.14
453	TRUE	28	28	0	0:34.20
454	TRUE	28	28	0	0:34.30
455	TRUE	28	28	0	0:34.23
456	TRUE	28	28	0	0:34.23
457	TRUE	28	28	0	0:34.28
458	TRUE	28	28	0	0:34.24
459	TRUE	28	28	0	0:34.22
460	TRUE	28	28	0	0:34.30
461	TRUE	28	28	0	0:34.33
462	TRUE	28	28	0	0:34.16
463	TRUE	28	28	0	0:34.20
464	TRUE	28	28	0	0:34.40
465	TRUE	28	28	0	0:34.31
466	TRUE	28	28	0	0:34.54
467	TRUE	28	28	0	0:34.36
468	TRUE	28	28	0	0:34.34
469	TRUE	28	28	0	0:34.21
470	TRUE	28	28	0	0:34.59
471	TRUE	28	28	0	0:34.48
472	TRUE	28	28	0	0:34.40
473	TRUE	28	28	0	0:34.31
474	TRUE	28	28	0	0:34.20
475	TRUE	28	28	0	0:34.04
476	TRUE	28	28	0	0:34.42
477	TRUE	28	28	0	0:34.34
478	TRUE	28	28	0	0:34.10
479	TRUE	28	28	0	0:34.17
480	TRUE	28	28	0	0:34.22
481	TRUE	28	28	0	0:34.25

Id	Result	#total	#true	#false	Execution time (min:sec)
482	TRUE	28	28	0	0:34.29
483	TRUE	28	28	0	0:34.50
484	TRUE	28	28	0	0:34.40
485	TRUE	28	28	0	0:34.21
486	TRUE	28	28	0	0:34.25
487	TRUE	28	28	0	0:34.22
488	TRUE	28	28	0	0:34.09
489	TRUE	28	28	0	0:34.25
490	TRUE	28	28	0	0:34.30
491	TRUE	28	28	0	0:34.30
492	TRUE	28	28	0	0:34.30
493	TRUE	28	28	0	0:34.11
494	TRUE	28	28	0	0:34.18
495	TRUE	28	28	0	0:34.26
496	TRUE	28	28	0	0:34.30
497	TRUE	28	28	0	0:34.25
498	TRUE	28	28	0	0:34.14
499	TRUE	28	28	0	0:34.14
500	TRUE	28	28	0	0:34.14
