# Supplementary material for: A Novel Method to Verify Multilevel Computational Models of Biological Systems Using Multiscale Spatio-Temporal Meta Model Checking
Source: PLoS One. 2016 May 17;11(5):e0154847. doi: 10.1371/journal.pone.0154847 (PMC4871515; doi:10.1371/journal.pone.0154847)
Supplement: S10 Text — (PDF) [file pone.0154847.s010.pdf]

Excerpts employed for writing the formal specification of the rat cardiovascular system dynamics case study

The excerpts from the referenced paper [1] employed for writing the formal specification of the rat cardiovascular system dynamics case study, together with the derived natural language and formal PBLMSTL statements are provided below.

Property 1

Excerpts

"During the interval marked Valsalva the thoracic pressure is increased from the baseline value of -4 mmHg to the value of 16 mmHg. After 10 s of elevated pressure, thoracic pressure is returned to the baseline value" [1].

Derived natural language statement

After initiating the Valsava manoeuvre (time = 5000 ms) the thoracic pressure increases from the baseline value -4 to 16 for 10 seconds (time interval [5001 ms, 14999 ms]), and then drops back to the baseline value -4, with probability greater than 0.9. The corresponding rephrased natural language statement is that the probability is greater than 0.9 that after initiating the Valsava manoeuvre (time = 5000 ms) the thoracic pressure {P_th} (corresponding to scale and subsystem OrganSystem.Cardiovascular) increases from the baseline value -4 to 16 for 10 seconds (time interval [5001 ms, 14999 ms]), and then drops back to the baseline value -4.

PBLMSTL statement

$$
\begin{aligned}
P > 0.9\ [&(G\ [0, 5000]\ (\{P\_th\}(\mathit{scaleAndSubsystem} = \\
&\mathit{OrganSystem.Cardiovascular}) = -4)) \wedge \\
&(G\ [5001, 14999]\ (\{P\_th\}(\mathit{scaleAndSubsystem} = \\
&\mathit{OrganSystem.Cardiovascular}) = 16)) \wedge \\
&(G\ [15000, 30000]\ (\{P\_th\}(\mathit{scaleAndSubsystem} = \\
&\mathit{OrganSystem.Cardiovascular}) = -4))]
\end{aligned}
$$

Property 2

Excerpts

"During the initial phase of the response ... the increase in aortic pressure causes a transient decrease in heart rate via the baroreflex" [1].

Derived natural language statement

The probability is greater than 0.9 that during the initial phase of the response (time interval [5001 ms, 6500 ms]) the aortic pressure increases and the heart rate decreases. The corresponding rephrased natural language statement is that the probability is greater than 0.9 that during the initial phase of the response (time interval [5001 ms, 6500 ms]) the aortic pressure {P_ao} (corresponding to scale and subsystem Cellular.Baroreceptor) increases and the heart rate {HR} (corresponding to scale and subsystem OrganSystem.Cardiovascular) decreases.

Since the values of {P_ao} and {HR} are continuously oscillating we check if the maximum {P_ao} value in time interval [5001 ms, 6500 ms] is larger than the maximum {P_ao} value in time interval [4800 ms, 5000 ms], respectively if the minimum {HR} value in time interval [5001 ms, 6500 ms] is smaller than the minimum {HR} value in time interval [4800 ms, 5000 ms].

PBLMSTL statement

$$
\begin{aligned}
P > 0.9\ [& (max([5001, 6500]\ \{P\_ao\}(scaleAndSubsystem = \\
& Cellular.Baroreceptor)) > \\
& max([4800, 5000]\ \{P\_ao\}(scaleAndSubsystem = \\
& Cellular.Baroreceptor))) \wedge \\
& (min([5001, 6500]\ \{HR\}(scaleAndSubsystem = \\
& OrganSystem.Cardiovascular)) < \\
& min([4800, 5000]\ \{HR\}(scaleAndSubsystem = \\
& OrganSystem.Cardiovascular)))]
\end{aligned}
$$

Property 3

Excerpts

"Following the initial response of increased" (aortic) "pressure, pressure begins to drop as a result of elevated thoracic pressure impeding venous flow to the heart. Heart rate increases in response to the reduction in pressure predicted over the second phase of the Valsalva interval" [1].

Derived natural language statement

The probability is less than 0.1 that after the initial response phase (time interval [5001 ms, 6500 ms]) the aortic pressure continues to increase or stay constant, respectively the heart rate continues to decrease or stay constant throughout the remainder of the Valsava interval (time interval [6501 ms, 14999 ms]). The corresponding rephrased natural language statement is that the probability is less than 0.1 that after the initial response phase (time interval [5001 ms, 6500 ms]) the aortic pressure {P_ao} (corresponding to scale and subsystem Cellular.Baroreceptor) continues to increase or stay constant, respectively the heart rate {HR} (corresponding to scale and subsystem OrganSystem.Cardiovascular) continues to decrease or stay constant throughout the remainder of the Valsava interval (time interval [6501 ms, 14999 ms]).

This statement can be rewritten as the minimum {P_ao} value in time interval [6501 ms, 14999 ms] is greater or equal to the maximum {P_ao} value in time interval [5001 ms, 6500 ms], respectively the maximum {HR} value in time interval [6501 ms, 14999 ms] is smaller or equal to the minimum {HR} value in time interval [5001 ms, 6500 ms] with probability less than 0.1.

Remark: The derived natural language statement is the negated version of the immediately above provided excerpt because the associated probability is expected to be less than 0.1. The only reason for employing the negated form of the excerpt is to illustrate the usage of the $P_{<\theta}$ probability specification.

PBLMSTL statement

$$
\begin{aligned}
P < 0.1\ [&(min([6501, 14999]\ \{P\_ao\}(scaleAndSubsystem = \\
&Cellular.Baroreceptor)) >= \\
&max([5001, 6500]\ \{P\_ao\}(scaleAndSubsystem = \\
&Cellular.Baroreceptor))) \wedge \\
&(max([6501, 14999]\ \{HR\}(scaleAndSubsystem = \\
&OrganSystem.Cardiovascular)) <= \\
&min([5001, 6500]\ \{HR\}(scaleAndSubsystem = \\
&OrganSystem.Cardiovascular)))]
\end{aligned}
$$

References

[1] Daniel A. Beard, Maxwell L. Neal, Nazanin Tabesh-Saleki, Christopher T. Thompson, James B. Bassingtwaighte, Mary Shimoyama, and Brian E. Carlson. Multiscale modeling and data integration in the virtual physiological rat project. *Annals of Biomedical Engineering*, 40(11):2365–2378, November 2012.
