# Supplementary material for: A Novel Method to Verify Multilevel Computational Models of Biological Systems Using Multiscale Spatio-Temporal Meta Model Checking
Source: PLoS One. 2016 May 17;11(5):e0154847. doi: 10.1371/journal.pone.0154847 (PMC4871515; doi:10.1371/journal.pone.0154847)
Supplement: S11 Text — (PDF) [file pone.0154847.s011.pdf]

Excerpts employed for writing the formal specification of the uterine contractions of labour case study

The excerpts from the referenced paper [1] employed for writing the formal specification of the uterine contractions of labour case study, together with the derived natural language and formal PBLMSTL statements are provided below.

Property 1

Excerpts

As indicated by the first rule in the "Formal presentation of the rules of the simulation" subsection of [1] the intrauterine pressure (*pressure*) is computed as the sum of all contractile activities (*act*) divided by the total number of regions (*#regions*) and their corresponding constant anatomic sensitivities (*anatomysens*):

$$pressure(t) = \sum act(i, j)/(\#regions * anatomysens(i, j)),$$

where t represents the current timepoint, respectively i and j encode Ox and Oy coordinates in the discretised 2D spatial domain.

Since both the total number of uterus regions and the associated anatomic sensitivities are constant throughout individual model simulations, the intrauterine pressure is directly proportional to the contractile activities of the uterus regions.

Derived natural language statement

The probability is greater than 0.9 that the intrauterine pressure increases/decreases with the contractile activity of uterine regions. The corresponding rephrased natural language statement is that the intrauterine pressure {Pressure} (corresponding to scale and subsystem Organ.Uterus) increases/decreases with the contractile activities (denoted in PBLMSTL as densities) of the uterus regions (corresponding to scale and subsystem Tissue.ContractileActivity) with probability greater than 0.9.

PBLMSTL statement

$$P > 0.9\ [G\ [1,329]\ (((d(\{Pressure\}(scaleAndSubsystem = Organ.Uterus)) > 0)\ \wedge ((d(sum(density(filter(regions, scaleAndSubsystem = Tissue.ContractileActivity)))) > 0))) \vee ((d(\{Pressure\}(scaleAndSubsystem = Organ.Uterus)) < 0)\ \wedge ((d(sum(density(filter(regions, scaleAndSubsystem = Tissue.ContractileActivity)))) < 0))) \vee (d(\{Pressure\}(scaleAndSubsystem = Organ.Uterus)) = 0))]$$

Property 2

Excerpts

"When a region is experiencing an action potential burst, the contractile activity is calculated by multiplying the passive tension by the action potential multiplier (a factor > 1)" [1].

"We assume that action potential bursts enhanced contractile activity via a multiplication factor (the action potential multiplier)" [1].

Derived natural language statement

The probability is less than 0.1 that the intrauterine pressure decreases when the entire uterus experiences an action potential burst. The corresponding rephrased natural language statement is that the probability is less than 0.1 that the intrauterine pressure {Pressure} (corresponding to scale and subsystem Organ.Uterus) decreases when the entire uterus comprising all 16 regions (corresponding to scale and subsystem Tissue.BurstActivity) experiences an action potential burst.

PBLMSTL statement

$$P < 0.1\ [F\ [1,329]\ ((d(\{Pressure\}(scaleAndSubsystem = Organ.Uterus)) < 0) \wedge (min(area(filter(regions, scaleAndSubsystem = Tissue.BurstActivity))) = 16))]$$

Property 3

Excerpts

"When a region is in the refractory period, the tension" (or pressure) "is decreased by multiplying the passive tension by a factor < 1 (refractory multi-

plier)" [1].

Derived natural language statement

The probability is greater than 0.9 that the intrauterine pressure decreases when the entire uterus is in the refractory period. The corresponding rephrased natural language statement is that the probability is greater than 0.9 that the intrauterine pressure {Pressure} (corresponding to scale and subsystem Organ.Uterus) decreases when the entire uterus comprising all 16 regions (corresponding to scale and subsystem Tissue.RefractoryActivity) is in the refractory period.

PBLMSTL statement

$$P > 0.9\ [G\ [1, 329]\ (((max(area(filter(regions, scaleAndSubsystem = Tissue.RefractoryActivity))) = 0)) \Rightarrow ((d(\{Pressure\}(scaleAndSubsystem = Organ.Uterus)) < 0) \vee ((d(\{Pressure\}(scaleAndSubsystem = Organ.Uterus)) = 0) \wedge (\{Pressure\}(scaleAndSubsystem = Organ.Uterus) = 0.5))))]$$

References

[1] Roger C. Young and Peter Barendse. Linking myometrial physiology to intrauterine pressure; how tissue-level contractions create uterine contractions of labor. *PLoS Comput Biol*, 10(10):e1003850, October 2014.
