# Supplementary material for: A Novel Method to Verify Multilevel Computational Models of Biological Systems Using Multiscale Spatio-Temporal Meta Model Checking
Source: PLoS One. 2016 May 17;11(5):e0154847. doi: 10.1371/journal.pone.0154847 (PMC4871515; doi:10.1371/journal.pone.0154847)
Supplement: S12 Text — (PDF) [file pone.0154847.s012.pdf]

Excerpts employed for writing the formal specification of the *Xenopus laevis* cell cycle case study

The excerpts from the referenced paper [1] employed for writing the formal specification of the *Xenopus laevis* cell cycle case study, together with the derived natural language and formal PBLMSTL statements are provided below.

Property 1

Excerpts

"The activation of CDK1 drives the cell into mitosis" [1].

Derived natural language statement

The probability is greater than 0.9 that whenever the concentration of CDK1 reaches very high levels (in our case >96% of its maximum value) all cells will divide. The corresponding rephrased natural language statement is that the probability is greater than 0.9 that if the concentration (denoted in PBLMSTL as density) of CDK1 (corresponding to scale and subsystem Intracellular.CDK1) increases above 0.96 then all cells will divide i.e. the sum of the (densities $\times$ areas) of all regions covered by cells (corresponding to scale and subsystem Cellular.Embryo) will increase.

The value of 96% corresponds to the normalized threshold concentration 0.5 for CDK1 ($[CDK1] \in [0, 0.515]$) chosen by the developers of the multiscale model [2] to trigger cellular division. Moreover the time interval considered in the PBLMSTL statements corresponds to the time interval considered in the model simulation.

PBLMSTL statement

$$
\begin{aligned}
P > 0.9\ [G\ [0,100]\ (((&count(density(filter(regions,\\
&scaleAndSubsystem = Intracellular.CDK1 \wedge\\
&density < 0.96))) = count(density(filter(regions,\\
&scaleAndSubsystem = Intracellular.CDK1)))) \wedge\\
&(X\ (count(density(filter(regions,\\
&scaleAndSubsystem = Intracellular.CDK1 \wedge\\
&density > 0.96))) = count(density(filter(regions,\\
&scaleAndSubsystem = Intracellular.CDK1))))))\\
&\Rightarrow (d(sum(multiply(area(filter(regions,\\
&scaleAndSubsystem = Cellular.Embryo)),\\
&density(filter(regions, scaleAndSubsystem =\\
&Cellular.Embryo))))) > 0))]
\end{aligned}
$$

Property 2

Excerpts

"the activation of APC, which generally lags behind CDK1, drives the cell back out of mitosis" [1].

Derived natural language statement

The probability is greater than 0.9 that whenever the average concentration of APC increases and reaches its local maximum value no cell will divide. The corresponding rephrased natural language statement is that the probability is greater than 0.9 that if the average concentration (represented in PBLMSTL as density) of APC (corresponding to scale and subsystem Intracellular.APC) reaches a local maximum value i.e. increases and then decreases, then no cell will divide i.e. the sum of the (densities × areas) of all regions covered by cells (corresponding to scale and subsystem Cellular.Embryo) will remain constant.

The time interval considered in the PBLMSTL statements corresponds to the time interval considered in the model simulation.

PBLMSTL statement

$$
\begin{aligned}
P > 0.9\ [G\ [0,100]\ (((&d(avg(density(filter(regions,\\
&scaleAndSubsystem = Intracellular.APC\\
&)))) > 0)\ \wedge\ (X\ (d(avg(density(filter(regions,\\
&scaleAndSubsystem = Intracellular.APC\\
&)))) < 0)))\ \Rightarrow\ (X\ (d(sum(multiply(\\
&area(filter(regions, scaleAndSubsystem =\\
&Cellular.Embryo)), density(filter(regions,\\
&scaleAndSubsystem = Cellular.Embryo))\\
&))) = 0)))]
\end{aligned}
$$

Property 3

Excerpts

Figure 5C in [1] illustrates the oscillatory evolution over time of the concentration of APC, CDK1 and Plk1. The oscillatory behaviour is additionally emphasized in the figure caption "Time course of the system, showing sustained limit cycle oscillations." [1].

Derived natural language statement

The probability is greater than 0.9 that the average concentrations of CDK1, Plk1 and APC increase and then decrease (i.e. oscillate) over time at least three times. The corresponding rephrased natural language statement is that the probability is greater than 0.9 that the average concentrations (represented

in PBLMSTL as densities) of CDK1, Plk1 and APC (corresponding to scale and subsystem Intracellular.CDK1, Intracellular.Plk1, respectively Intracellular.APC) increase and then decrease over time at least three times.

The minimum number of oscillations (in our case three) was chosen considering the number of oscillations displayed in [1, Figure 5C]. Moreover the time interval considered in the PBLMSTL statements corresponds to the time interval considered in the model simulation.

PBLMSTL statement

$$
\begin{aligned}
P > 0.9\ [(&F\ [0,100]\ ((d(avg(density(filter(regions,\\
&scaleAndSubsystem = Intracellular.CDK1\\
&)))) > 0)\ \wedge\ (F\ [0,100]\ ((d(avg(density\\
&(filter(regions, scaleAndSubsystem =\\
&Intracellular.CDK1)))) < 0)\ \wedge\\
&(F\ [0,100]\ ((d(avg(density(filter(regions,\\
&scaleAndSubsystem = Intracellular.CDK1\\
&)))) > 0)\ \wedge\ (F\ [0,100]\ ((d(avg(density\\
&(filter(regions, scaleAndSubsystem =\\
&Intracellular.CDK1)))) < 0)\ \wedge\\
&(F\ [0,100]\ ((d(avg(density(filter(regions,\\
&scaleAndSubsystem = Intracellular.CDK1\\
&)))) > 0)\ \wedge\ (F\ [0,100]\ ((d(avg(density\\
&(filter(regions, scaleAndSubsystem =\\
&Intracellular.CDK1)))) < 0)\\
&)))))))))))\ \wedge\\
&(F\ [0,100]\ ((d(avg(density(filter(regions,\\
&scaleAndSubsystem = Intracellular.Plk1\\
&)))) > 0)\ \wedge\ (F\ [0,100]\ ((d(avg(density\\
&(filter(regions, scaleAndSubsystem =\\
&Intracellular.Plk1)))) < 0)\ \wedge\\
&(F\ [0,100]\ ((d(avg(density(filter(regions,\\
&scaleAndSubsystem = Intracellular.Plk1\\
&)))) > 0)\ \wedge\ (F\ [0,100]\ ((d(avg(density\\
&(filter(regions, scaleAndSubsystem =\\
&Intracellular.Plk1)))) < 0)\ \wedge\\
&(F\ [0,100]\ ((d(avg(density(filter(regions,\\
&scaleAndSubsystem = Intracellular.Plk1\\
&)))) > 0)\ \wedge
\end{aligned}
$$

$$
\begin{aligned}
&(F\ [0,100]\ ((d(avg(density\\
&(filter(regions, scaleAndSubsystem =\\
&Intracellular.Plk1)))) < 0)\\
&)))))))))))) \wedge\\
&(F\ [0,100]\ ((d(avg(density(filter(regions,\\
&scaleAndSubsystem = Intracellular.APC\\
&)))) > 0)\ \wedge\ (F\ [0,100]\ ((d(avg(density\\
&(filter(regions, scaleAndSubsystem =\\
&Intracellular.APC)))) < 0) \wedge\\
&(F\ [0,100]\ ((d(avg(density(filter(regions,\\
&scaleAndSubsystem = Intracellular.APC\\
&)))) > 0)\ \wedge\ (F\ [0,100]\ ((d(avg(density\\
&(filter(regions, scaleAndSubsystem =\\
&Intracellular.APC)))) < 0) \wedge\\
&(F\ [0,100]\ ((d(avg(density(filter(regions,\\
&scaleAndSubsystem = Intracellular.APC\\
&)))) > 0)\ \wedge\ (F\ [0,100]\ ((d(avg(density\\
&(filter(regions, scaleAndSubsystem =\\
&Intracellular.APC)))) < 0)\\
&))))))))))))]
\end{aligned}
$$

References

[1] James E. Ferrell Jr., Tony Yu-Chen Tsai, and Qiong Yang. Modeling the cell cycle: Why do certain circuits oscillate? *Cell*, 144(6):874–885, March 2011.

[2] Jörn Starruß and Walter de Back. Morpheus examples. http://imc.zih.tu-dresden.de/wiki/morpheus/doku.php?id=examples:examples.
