# Supplementary material for: A Novel Method to Verify Multilevel Computational Models of Biological Systems Using Multiscale Spatio-Temporal Meta Model Checking
Source: PLoS One. 2016 May 17;11(5):e0154847. doi: 10.1371/journal.pone.0154847 (PMC4871515; doi:10.1371/journal.pone.0154847)
Supplement: S13 Text — (PDF) [file pone.0154847.s013.pdf]

Excerpts employed for writing the formal specification of the acute inflammation of the gut and lung case study

The excerpts from the referenced paper [1] employed for writing the formal specification of the acute inflammation of the gut and lung case study, together with the derived natural language and formal PBLMSTL statements are provided below.

Property 1

Excerpts

“tight junction (TJ) proteins are involved in the integrity of gut epithelial barrier function” ... “The TJ proteins that seem to be most affected in this situation are occludin ...” [1].

“pulmonary epithelial cells behave very similarly to gut epithelial cells with respect to tight junction metabolism and epithelial barrier function” [1].

“The impaired systemic oxygenation due to pulmonary leak arises from pulmonary epithelial barrier failure” [1].

“impaired oxygenation into the endothelial lumen, which is summed across the surface of the model to produce a measure of systemic arterial oxygen content. This value will now represent the baseline ”oxy” level for all other systemic endothelial agents” [1].

“ischemia was modeled as a percentage of the total endothelial surface rendered ”ischemic,” a state defined in the rules for the endothelial cell agents as an ”oxy” level < 60” [1].

Derived natural language statement

The probability is greater than 0.9 that if the level of cytoplasm occludin in the lung decreases then eventually the number of ischemic endothelial lung cells will increase. The corresponding rephrased natural language statement is that the probability is greater than 0.9 that if the value of {LungOccludinCytoplasm} (corresponding to scale and subsystem Tissue.LungEpithelium) decreases then eventually the total area of the regions defined by ischemic endothelial lung cells (corresponding to scale and subsystem Cellular.LungEndotheliumIschemia) will increase.

PBLMSTL statement

$$P > 0.9\ [F\ [1,999]\ ((d(\{LungOccludinCytoplasm\}(scaleAndSubsystem = Tissue.LungEpithelium)) < 0) \Rightarrow (F\ [1,999]\ (d(sum(area(filter(regions, scaleAndSubsystem = Cellular.LungEndotheliumIschemia)))) > 0)))]$$

Property 2

Excerpts

"this variable is termed "cell-damage-byproduct," and it is calculated as a function of total endothelial damage" [1].

"the levels of "cell-damage-byproduct" will be the proxy for the unidentified compound that is produced in the ischemic gut and circulated to the lung, leading to inflammation of pulmonary endothelium" [1].

Derived natural language statement

The probability is greater than 0.9 that always an increase of the cell damage by-product in the gut will lead to an increase of the cell damage by-product in the lung. The corresponding rephrased natural language statement is that the probability is greater than 0.9 that always if the value of {GutCellDamageByproduct} (corresponding to scale and subsystem Tissue.GutEndothelium) increases, then eventually the value of {LungCellDamageByproduct} (corresponding to scale and subsystem Tissue.LungEndothelium) increases.

PBLMSTL statement

$$P > 0.9\ [G\ [1,999]\ ((d(\{GutCellDamageByproduct\}(scaleAndSubsystem = Tissue.GutEndothelium)) > 0) \Rightarrow (F\ [1,999]\ (d(\{LungCellDamageByproduct\}(scaleAndSubsystem = Tissue.LungEndothelium)) > 0)))]$$

Property 3

Excerpts

"tight junction (TJ) proteins are involved in the integrity of gut epithelial barrier function" ... "The TJ proteins that seem to be most affected in this situation are occludin ..." [1].

“A luminal compound that diffuses in response to TJ barrier failure” ... “is represented by ”gut-leak,” which is equal to the ”solute”” ... “that penetrates the failed barrier” [1].

Derived natural language statement

The probability is greater than 0.9 that if the level of cell wall occludin in the gut decreases then eventually the amount of solute leaking in the gut lumen will increase. The corresponding rephrased natural language statement is that the probability is greater than 0.9 that if the value of {GutOccludinCellwall} (corresponding to scale and subsystem Tissue.GutEpithelium) decreases then eventually the value of {GutLeak} (corresponding to scale and subsystem Organ.Gut) will increase.

PBLMSTL statement

$$
\begin{aligned}
P > 0.9\ [F\ [1,999]\ ((d(\{GutOccludinCellwall\}(\\
&scaleAndSubsystem = Tissue.GutEpithelium))\\
&< 0)\ \Rightarrow\ (F\ [1,999]\ (d(\{GutLeak\}(\\
&scaleAndSubsystem = Organ.Gut)) > 0)))]
\end{aligned}
$$

References

[1] Gary An. Introduction of an agent-based multi-scale modular architecture for dynamic knowledge representation of acute inflammation. *Theoretical Biology and Medical Modelling*, 5(1):11, May 2008.
